# Supplementary figures and images for: Factors associated with self-reported diagnosed asthma in urban and rural Malawi: Observations from a population-based study of non-communicable diseases
Source: PLOS Glob Public Health. 2024 Jul 11;4(7):e0002952. doi: 10.1371/journal.pgph.0002952 (PMC11239063; doi:10.1371/journal.pgph.0002952)

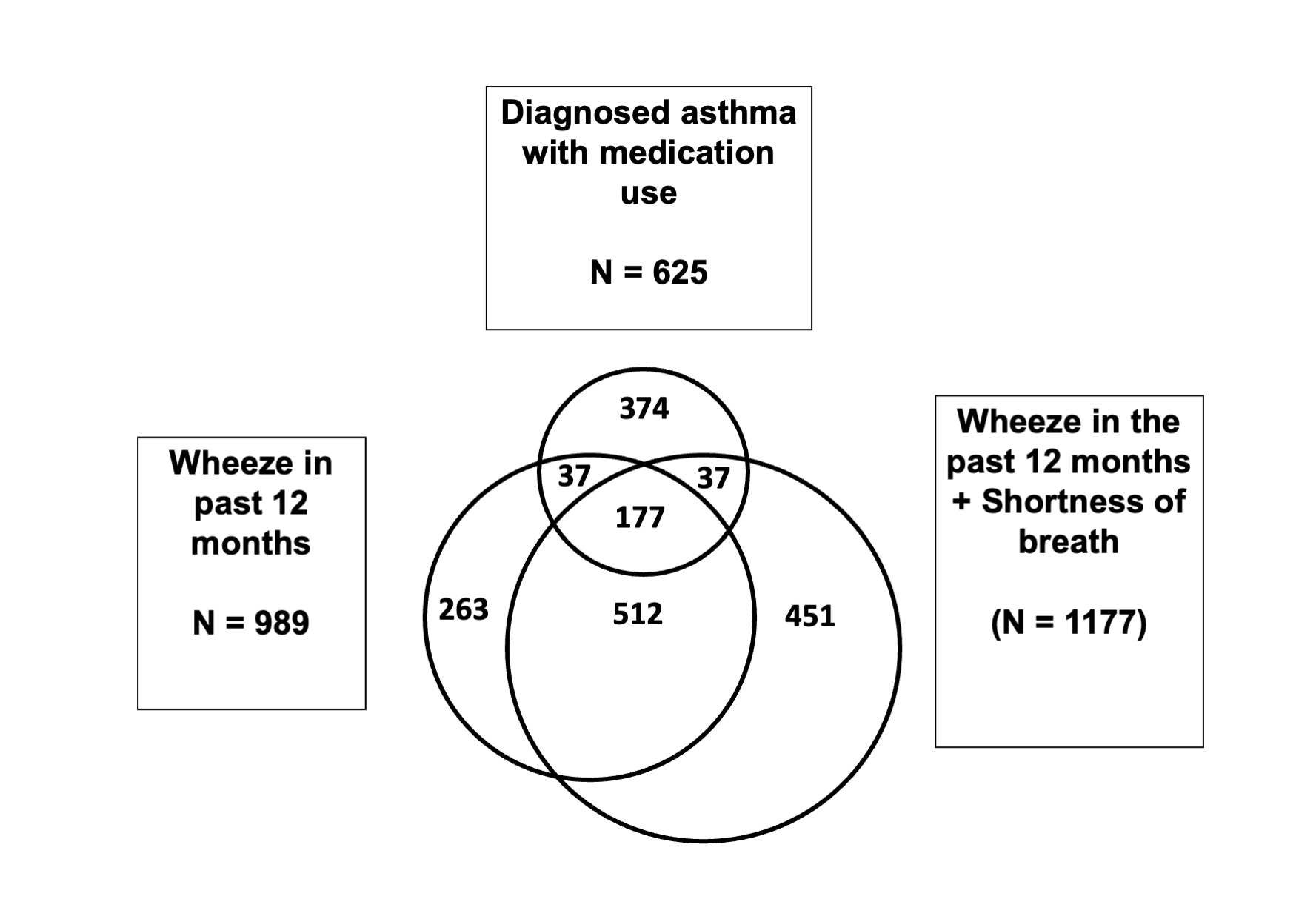

Supplement: S1 Fig — Figure is a proportional Venn diagram showing the overlap between the outcomes ‘Diagnosed asthma with medication use’, ‘Wheeze in the past 12 months’ and ‘Wheeze in the past 12 months plus shortness of breath’. The area representing each variable is proportional to the number of people reporting this outcome. (TIFF) [file pgph.0002952.s001.tiff]
