# Supplementary material for: Factors associated with self-reported diagnosed asthma in urban and rural Malawi: Observations from a population-based study of non-communicable diseases
Source: PLOS Glob Public Health. 2024 Jul 11;4(7):e0002952. doi: 10.1371/journal.pgph.0002952 (PMC11239063; doi:10.1371/journal.pgph.0002952)
Supplement: S1 Table — (DOCX) [file pgph.0002952.s002.docx]

Table S1: Univariable Analysis - factors associated with diagnosed asthma stratified by sex & area

| **Factors** | **Female** | | | | **Male** | | | |
| --- | --- | --- | --- | --- | --- | --- | --- | --- |
|  | **LILONGWE - URBAN** | | **KARONGA - RURAL** | | **LILONGWE - URBAN** | | **KARONGA - RURAL** | |
|  | **Crude Odds ratio (95%CI)** | **p-value** | **Crude Odds ratio (95%CI)** | **p-value** | **Crude Odds ratio (95%CI)** | **p-value** | **Crude Odds ratio (95%CI)** | **p-value** |
| **Age group** |  |  |  |  |  |  |  |  |
| 18-29 | 1.00 | - | 1.00 | - | 1.00 | - | 1.00 | - |
| 30-39 | **1.35 (1.12 - 1.63)** | **0.002** | **1.28 (0.98 - 1.67)** | **0.072** | 0.98 (0.72 - 1.34) | 0.911 | 0.97 (0.71 - 1.33) | 0.859 |
| 40-49 | 1.14 (0.86 - 1.51) | 0.371 | 1.13 (0.82 - 1.55) | 0.464 | 1.10 (0.75 - 1.63) | 0.614 | **0.64 (0.42 - 0.97)** | **0.038** |
| 50-59 | **1.59 (1.15 - 2.19)** | **0.005** | 0.88 (0.59 - 1.31) | 0.530 | 0.85 (0.48 - 1.49) | 0.569 | 0.84 (0.54 - 1.33) | 0.461 |
| 60-69 | 1.24 (0.77 - 2.00) | 0.380 | 1.39 (0.92 - 2.10) | 0.115 | 0.73 (0.36 - 1.52) | 0.404 | 0.70 (0.37 - 1.32) | 0.268 |
| 70+ | 1.47 (0.81 - 2.67) | 0.210 | 0.69 (0.40 - 1.22) | 0.202 | 0.67 (0.24 - 1.84) | 0.439 | 0.90 (0.53 - 1.52) | 0.689 |
|  |  |  |  |  |  |  |  |  |
| **Body Mass Index** |  |  |  |  |  |  |  |  |
| Underweight (<18.5) | 0.99 (0.62 - 1.60) | 0.983 | 1.20 (0.79 - 1.80) | 0.395 | 1.10 (0.68 - 1.78) | 0.713 | 1.35 (0.92 - 1.98) | 0.127 |
| Normal(18.5-24.9) | 1.00 | - | 1.00 | - | 1.00 | - | 1.00 | - |
| Overweight (25.0-29.9) | **1.42 (1.16 - 1.73)** | **0.001** | 1.00 (0.75 - 1.33) | 0.992 | 1.11 (0.78 - 1.57) | 0.573 | 1.01 (0.63 - 1.62) | 0.961 |
| Obese(>30.0) | **1.92 (1.55 - 2.36)** | **<0.001** | **1.40 (0.97 - 2.04)** | **0.074** | 1.16 (0.62 - 2.17) | 0.634 | 1.20 (0.37 - 3.87) | 0.759 |
|  |  |  |  |  |  |  |  |  |
| **Physical activity rating** |  |  |  |  |  |  |  |  |
| Low | 1.00 | - | 1.00 | - | 1.00 | - | 1.00 | - |
| Moderate | 0.82 (0.41 - 1.65) | 0.585 | 0.88 (0.31 - 2.48) | 0.813 | 1.10 (0.58 - 2.08) | 0.764 | **0.27 (0.12 - 0.60)** | **0.001** |
| High | **0.64 (0.37 - 1.09)** | **0.099** | 1.13 (0.50 - 2.58) | 0.765 | 0.74 (0.41 - 1.31) | 0.303 | **0.53 (0.29 - 0.97)** | **0.040** |
|  |  |  |  |  |  |  |  |  |
| **Household income** | **1.07 (1.04 - 1.11)** | **<0.001** | 1.03 (0.98 - 1.08) | 0.226 | **1.05 (1.00 - 1.11)** | **0.052** | **1.10 (1.04 - 1.16)** | **0.001** |
| **Household possessions** | **1.09 (1.06 - 1.13)** | **<0.001** | 1.03 (0.99 - 1.08) | 0.110 | **1.07 (1.02 - 1.13)** | **0.007** | **1.06 (1.01 - 1.12)** | **0.019** |
|  |  |  |  |  |  |  |  |  |
| **Education** |  |  |  |  |  |  |  |  |
| No formal | **0.57 (0.33 - 0.96)** | **0.036** | **1.67 (0.97 - 2.86)** | **0.063** | **3.62 (0.88 - 14.83)** | **0.074** | 0.39 (0.05 - 3.01) | 0.369 |
| Primary: Standard 1-5 | 1.00 | - | 1.00 | - | 1.00 | - | 1.00 | - |
| Primary: Standard 6-8 | **0.58 (0.42 - 0.81)** | **0.001** | **1.71 (1.20 - 2.45)** | **0.003** | **2.92 (1.03 - 8.31)** | **0.044** | **1.60 (0.94 - 2.74)** | **0.084** |
| Secondary | 0.91 (0.69 - 1.20) | 0.506 | **2.10 (1.44 - 3.07)** | **<0.001** | **2.81 (1.03 - 7.65)** | **0.044** | **2.14 (1.26 - 3.62)** | **0.005** |
| Tertiary | **1.58 (1.16 - 2.15)** | **0.004** | **4.11 (1.84 - 9.15)** | **0.001** | **4.21 (1.53 - 11.62)** | **0.005** | **3.10 (1.46 - 6.57)** | **0.003** |
|  |  |  |  |  |  |  |  |  |
| **Work Status** |  |  |  |  |  |  |  |  |
| Not working | 1.00 | - | 1.00 | - | 1.00 | - | 1.00 | - |
| Housework | 0.91 (0.72 - 1.13) | 0.380 | 0.76 (0.47 - 1.22) | 0.252 | 0.95 (0.55 - 1.64) | 0.860 | 1.86 (0.82 - 4.25) | 0.140 |
| Farming/fishing | 0.66 (0.16 - 2.75) | 0.573 | **0.73 (0.52 - 1.03)** | **0.075** | 0.71 (0.10 - 5.30) | 0.742 | **0.66 (0.48 - 0.90)** | **0.010** |
| Self-employed | **1.32 (1.03 - 1.70)** | **0.028** | 0.87 (0.57 - 1.32) | 0.506 | 1.11 (0.79 - 1.54) | 0.548 | **0.68 (0.44 - 1.05)** | **0.079** |
| Employed | **1.49 (1.16 - 1.91)** | **0.002** | 1.60 (0.89 - 2.88) | 0.120 | 0.81 (0.60 - 1.09) | 0.168 | 1.16 (0.75 - 1.79) | 0.518 |
|  |  |  |  |  |  |  |  |  |
| **Diagnosed Diabetes Mellitus** |  |  |  |  |  |  |  |  |
| No | 1.00 | - | 1.00 | - | 1.00 | - | 1.00 | - |
| Yes | 1.28 (0.69 - 2.37) | 0.438 | 1.30 (0.47 - 3.59) | 0.612 | 1.00 (0.36 - 2.74) | 0.994 | *no output* | - |
|  |  |  |  |  |  |  |  |  |
| **Diagnosed Heart disease** |  |  |  |  |  |  |  |  |
| No | 1.00 | - | 1.00 | - | 1.00 | - | 1.00 | - |
| Yes | **1.81 (1.21 - 2.72)** | **0.004** | **2.41 (1.44 - 4.02)** | **0.001** | 1.18 (0.37 - 3.78) | 0.785 | 0.98 (0.24 - 4.07) | 0.980 |
|  |  |  |  |  |  |  |  |  |
| **Diagnosed High Blood Pressure** |  |  |  |  |  |  |  |  |
| No | 1.00 | - | 1.00 | - | 1.00 | - | 1.00 | - |
| Yes | **1.35 (1.08 - 1.68)** | **0.008** | 1.21 (0.84 - 1.73) | 0.300 | 1.17 (0.75 - 1.81) | 0.494 | 1.23 (0.68 - 2.24) | 0.490 |
|  |  |  |  |  |  |  |  |  |
| **Diagnosed Stroke** |  |  |  |  |  |  |  |  |
| No | 1.00 | - | 1.00 | - | 1.00 | - | 1.00 | - |
| Yes | 1.71 (0.78 - 3.74) | 0.179 | 2.17 (0.86 - 5.47) | 0.102 | 2.01 (0.72 - 5.63) | 0.185 | 1.44 (0.45 - 4.68) | 0.539 |
|  |  |  |  |  |  |  |  |  |
| **Piped water at home** |  |  |  |  |  |  |  |  |
| No | 1.00 | - | 1.00 | - | 1.00 | - | 1.00 | - |
| Yes | **1.38 (1.17 - 1.63)** | **<0.001** | **1.50 (1.06 - 2.12)** | **0.022** | 1.17 (0.91 - 1.51) | 0.214 | **1.60 (1.09 - 2.35)** | **0.016** |
|  |  |  |  |  |  |  |  |  |
| **Smoking status** |  |  |  |  |  |  |  |  |
| Never smoked | 1.00 | - | 1.00 | - | 1.00 | - | 1.00 | - |
| Former smoker (stopped more than 6 months ago) | **4.49 (2.14 - 9.40)** | **<0.001** | *no output* | - | 1.02 (0.64 - 1.63) | 0.928 | 0.95 (0.52 - 1.72) | 0.863 |
| Current (in last 6 months) | 0.65 (0.09 - 4.81) | 0.675 | *no output* | - | 0.80 (0.49 - 1.30) | 0.359 | **0.41 (0.24 - 0.68)** | **0.001** |
|  |  |  |  |  |  |  |  |  |
| **Smoker in household** |  |  |  |  |  |  |  |  |
| No | 1.00 | - | 1.00 | - | 1.00 | - | 1.00 | - |
| Yes | 0.91 (0.69 - 1.20) | 0.495 | 1.04 (0.78 - 1.39) | 0.783 | 1.08 (0.70 - 1.67) | 0.732 | 0.84 (0.54 - 1.31) | 0.431 |
|  |  |  |  |  |  |  |  |  |
| **Firewood smoke exposure** |  |  |  |  |  |  |  |  |
| No/former/little exposure | 1.00 | - | 1.00 | - | 1.00 | - | 1.00 | - |
| Exposed, ventilated cooking area | 1.05 (0.79 - 1.39) | 0.741 | 0.72 (0.43 - 1.21) | 0.217 | **0.76 (0.57 - 1.01)** | **0.060** | 1.07 (0.83 - 1.39) | 0.602 |
| Exposed, non-ventilated cooking area | 0.83 (0.57 - 1.21) | 0.340 | 1.02 (0.29 - 3.64) | 0.977 | **0.56 (0.33 - 0.94)** | **0.030** | 0.95 (0.13 - 7.08) | 0.958 |

- Unadjusted ORs, 95% CIs and p-values in boldface indicate model results where p<0.100
- *no output*: models with no output for this level due to small numbers
