# Supplementary material for: Factors associated with self-reported diagnosed asthma in urban and rural Malawi: Observations from a population-based study of non-communicable diseases
Source: PLOS Glob Public Health. 2024 Jul 11;4(7):e0002952. doi: 10.1371/journal.pgph.0002952 (PMC11239063; doi:10.1371/journal.pgph.0002952)
Supplement: S2 Table — (DOCX) [file pgph.0002952.s003.docx]

Table S2: Univariable Analysis - factors associated with wheeze in the past 12 months stratified by sex & urban—rural area

| **Factors** | **Female** | | | | **Male** | | | |
| --- | --- | --- | --- | --- | --- | --- | --- | --- |
|  | **LILONGWE - URBAN** | | **KARONGA - RURAL** | | **LILONGWE - URBAN** | | **KARONGA - RURAL** | |
|  | **Crude Odds ratio (95%CI)** | **p-value** | **Crude Odds ratio (95%CI)** | **p-value** | **Crude Odds ratio (95%CI)** | **p-value** | **Crude Odds ratio (95%CI)** | **p-value** |
| **Age group** |  |  |  |  |  |  |  |  |
| 18-29 | 1.00 | - | 1.00 | - | 1.00 | - | 1.00 | - |
| 30-39 | 1.06 (0.82 - 1.36) | 0.659 | 1.20 (0.85 - 1.69) | 0.290 | 1.12 (0.76 - 1.65) | 0.551 | 1.00 (0.69 - 1.47) | 0.986 |
| 40-49 | 1.27 (0.91 - 1.78) | 0.165 | **1.50 (1.04 - 2.17)** | **0.031** | 1.18 (0.72 - 1.92) | 0.518 | 1.27 (0.85 - 1.92) | 0.245 |
| 50-59 | **2.00 (1.39 - 2.88)** | **<0.001** | **1.56 (1.03 - 2.36)** | **0.037** | 1.60 (0.91 - 2.82) | 0.101 | 0.77 (0.43 - 1.37) | 0.370 |
| 60-69 | 1.24 (0.69 - 2.26) | 0.472 | **2.06 (1.32 - 3.24)** | **0.002** | 1.46 (0.72 - 2.95) | 0.294 | 1.32 (0.72 - 2.42) | 0.367 |
| 70+ | 1.76 (0.88 - 3.50) | 0.108 | 1.07 (0.59 - 1.94) | 0.831 | 1.80 (0.77 - 4.20) | 0.177 | 1.25 (0.71 - 2.21) | 0.440 |
|  |  |  |  |  |  |  |  |  |
| **Body Mass Index** |  |  |  |  |  |  |  |  |
| Underweight (<18.5) | 1.07 (0.60 - 1.91) | 0.812 | 1.19 (0.72 - 1.96) | 0.497 | 1.36 (0.80 - 2.31) | 0.258 | 1.23 (0.77 - 1.96) | 0.382 |
| Normal(18.5-24.9) | 1.00 | - | 1.00 | - | 1.00 | - | 1.00 | - |
| Overweight (25.0-29.9) | **1.39 (1.08 - 1.79)** | **0.009** | 1.17 (0.84 - 1.63) | 0.357 | 0.88 (0.56 - 1.40) | 0.597 | **1.66 (1.06 - 2.61)** | **0.027** |
| Obese(>30.0) | **1.59 (1.21 - 2.09)** | **0.001** | **1.80 (1.20 - 2.71)** | **0.005** | 0.76 (0.31 - 1.89) | 0.559 | 2.32 (0.83 - 6.48) | 0.110 |
|  |  |  |  |  |  |  |  |  |
| **Physical activity rating** |  |  |  |  |  |  |  |  |
| Low | 1.00 | - | 1.00 | - | 1.00 | - | 1.00 | - |
| Moderate | 1.34 (0.64 - 2.82) | 0.443 | 0.72 (0.28 - 1.83) | 0.492 | 1.79 (0.74 - 4.32) | 0.195 | **0.35 (0.15 - 0.82)** | **0.016** |
| High | **0.51 (0.27 - 0.95)** | **0.032** | 0.57 (0.28 - 1.17) | 0.126 | 1.07 (0.47 - 2.46) | 0.869 | **0.53 (0.26 - 1.05)** | **0.068** |
|  |  |  |  |  |  |  |  |  |
| **Household income** | **1.08 (1.03 - 1.12)** | **<0.001** | **1.06 (1.00 - 1.11)** | **0.039** | **1.06 (1.00 - 1.13)** | **0.058** | **1.14 (1.07 - 1.21)** | **<0.001** |
| **Household possessions** | **1.12 (1.07 - 1.17)** | **<0.001** | **1.06 (1.01 - 1.11)** | **0.018** | 1.00 (0.95 - 1.06) | 0.877 | 1.02 (0.96 - 1.08) | 0.614 |
|  |  |  |  |  |  |  |  |  |
| **Education** |  |  |  |  |  |  |  |  |
| No formal | 0.64 (0.35 - 1.18) | 0.155 | 1.11 (0.63 - 1.93) | 0.724 | *no output* | - | 0.85 (0.19 - 3.78) | 0.832 |
| Primary: Standard 1-5 | 1.00 | - | 1.00 | - | 1.00 | - | 1.00 | - |
| Primary: Standard 6-8 | **0.62 (0.42 - 0.92)** | **0.016** | 0.92 (0.65 - 1.30) | 0.620 | 1.00 (0.48 - 2.08) | 0.995 | 1.20 (0.68 - 2.11) | 0.528 |
| Secondary | 0.77 (0.55 - 1.07) | 0.117 | 0.96 (0.65 - 1.42) | 0.836 | 0.70 (0.36 - 1.37) | 0.299 | **1.74 (1.01 - 3.02)** | **0.047** |
| Tertiary | 1.12 (0.77 - 1.65) | 0.553 | **3.35 (1.52 - 7.39)** | **0.003** | 0.85 (0.42 - 1.72) | 0.657 | 1.47 (0.56 - 3.83) | 0.436 |
|  |  |  |  |  |  |  |  |  |
| **Work Status** |  |  |  |  |  |  |  |  |
| Not working | 1.00 | - | 1.00 | - | 1.00 | - | 1.00 | - |
| Housework | **0.72 (0.55 - 0.94)** | **0.016** | **0.53 (0.29 - 0.95)** | **0.032** | **0.16 (0.04 - 0.67)** | **0.012** | 0.80 (0.19 - 3.38) | 0.759 |
| Farming/fishing | **2.94 (1.23 - 7.03)** | **0.015** | **0.65 (0.44 - 0.96)** | **0.032** | 1.02 (0.14 - 7.62) | 0.982 | 0.85 (0.58 - 1.25) | 0.398 |
| Self-employed | 1.00 (0.73 - 1.36) | 0.987 | 0.85 (0.53 - 1.37) | 0.504 | 1.11 (0.75 - 1.64) | 0.597 | 0.92 (0.56 - 1.52) | 0.749 |
| Employed | 0.98 (0.71 - 1.35) | 0.892 | **1.81 (0.96 - 3.41)** | **0.068** | 0.78 (0.54 - 1.11) | 0.169 | 1.35 (0.80 - 2.27) | 0.259 |
|  |  |  |  |  |  |  |  |  |
| **Diagnosed Diabetes Mellitus** |  |  |  |  |  |  |  |  |
| No | 1.00 | - | 1.00 | - | 1.00 | - | 1.00 | - |
| Yes | 0.74 (0.27 - 2.01) | 0.557 | 0.44 (0.06 - 3.19) | 0.417 | 0.74 (0.18 - 3.04) | 0.678 | 1.50 (0.36 - 6.27) | 0.576 |
|  |  |  |  |  |  |  |  |  |
| **Diagnosed Heart disease** |  |  |  |  |  |  |  |  |
| No | 1.00 | - | 1.00 | - | 1.00 | - | 1.00 | - |
| Yes | 5.80 (4.13 - 8.14) | <0.001 | 3.27 (1.92 - 5.56) | <0.001 | 5.47 (2.55 - 11.72) | <0.001 | 6.37 (2.93 - 13.85) | <0.001 |
|  |  |  |  |  |  |  |  |  |
| **Diagnosed High Blood Pressure** |  |  |  |  |  |  |  |  |
| No | 1.00 | - | 1.00 | - | 1.00 | - | 1.00 | - |
| Yes | **1.89 (1.47 - 2.44)** | **<0.001** | **2.55 (1.83 - 3.54)** | **<0.001** | 0.97 (0.55 - 1.72) | 0.918 | **1.86 (1.04 - 3.32)** | **0.036** |
|  |  |  |  |  |  |  |  |  |
| **Diagnosed Stroke** |  |  |  |  |  |  |  |  |
| No | 1.00 | - | 1.00 | - | 1.00 | - | 1.00 | - |
| Yes | 2.00 (0.80 - 4.99) | 0.135 | **2.43 (0.87 - 6.78)** | **0.090** | 1.46 (0.35 - 6.08) | 0.601 | 1.27 (0.31 - 5.28) | 0.740 |
|  |  |  |  |  |  |  |  |  |
| **Piped water at home** |  |  |  |  |  |  |  |  |
| No | 1.00 | - | 1.00 | - | 1.00 | - | 1.00 | - |
| Yes | **1.50 (1.22 - 1.85)** | **<0.001** | 1.12 (0.71 - 1.76) | 0.628 | 1.02 (0.74 - 1.39) | 0.920 | 1.21 (0.75 - 1.96) | 0.436 |
|  |  |  |  |  |  |  |  |  |
| **Smoking status** |  |  |  |  |  |  |  |  |
| Never smoked | 1.00 | - | 1.00 | - | 1.00 | - | 1.00 | - |
| Former smoker (stopped more than 6 months ago) | 1.39 (0.33 - 5.75) | 0.653 | *no output* | - | 1.23 (0.71 - 2.11) | 0.460 | 1.45 (0.80 - 2.65) | 0.225 |
| Current (in last 6 months) | 2.27 (0.54 - 9.63) | 0.265 | **5.04 (1.12 - 22.65)** | **0.035** | 1.29 (0.78 - 2.13) | 0.321 | 1.11 (0.74 - 1.67) | 0.621 |
|  |  |  |  |  |  |  |  |  |
| **Smoker in household** |  |  |  |  |  |  |  |  |
| No | 1.00 | - | 1.00 | - | 1.00 | - | 1.00 | - |
| Yes | 1.28 (0.94 - 1.75) | 0.116 | 1.04 (0.74 - 1.45) | 0.842 | 1.14 (0.67 - 1.92) | 0.630 | 1.17 (0.74 - 1.84) | 0.499 |
|  |  |  |  |  |  |  |  |  |
| **Firewood smoke exposure** |  |  |  |  |  |  |  |  |
| No/former/little exposure | 1.00 | - | 1.00 | - | 1.00 | - | 1.00 | - |
| Exposed, ventilated cooking area | **0.71 (0.52 - 0.98)** | **0.036** | **0.60 (0.34 - 1.04)** | **0.070** | **0.60 (0.43 - 0.86)** | **0.005** | **1.46 (1.08 - 1.97)** | **0.014** |
| Exposed, non-ventilated cooking area | **0.64 (0.41 - 0.99)** | **0.045** | *no output* | - | 0.97 (0.58 - 1.61) | 0.892 | *no output* | - |

- Unadjusted ORs, 95% CIs and p-values in boldface indicate model results where p<0.100
- *no output*: models with no output for this level due to small numbers
