# Supplementary material for: Factors associated with self-reported diagnosed asthma in urban and rural Malawi: Observations from a population-based study of non-communicable diseases
Source: PLOS Glob Public Health. 2024 Jul 11;4(7):e0002952. doi: 10.1371/journal.pgph.0002952 (PMC11239063; doi:10.1371/journal.pgph.0002952)
Supplement: Attachment — Submitted filename: PGPH-D-23-01533_R1.pdf [file pgph.0002952.s005.pdf]

PLOS Global Public Health

Factors associated with self-reported diagnosed asthma in urban and rural Malawi: observations from a population-based study of non-communicable diseases

--Manuscript Draft--

**Manuscript Number:**	PGPH-D-23-01533R1
**Article Type:**	Research Article
**Full Title:**	Factors associated with self-reported diagnosed asthma in urban and rural Malawi: observations from a population-based study of non-communicable diseases
**Short Title:**	Factors associated with self-reported diagnosed asthma in urban and rural Malawi
**Corresponding Author:**	Abena Amoah, PhD<br>Leiden University Medical Center: Leids Universitair Medisch Centrum<br>Leiden, NETHERLANDS
**Order of Authors:**	Abena S. Amoah
	Estelle McLean
	Alison J. Price
	Alemayehu Amberbir
	Amelia C. Crampin
**Keywords:**	Asthma; wheeze; Epidemiology; noncommunicable diseases; urbanization; obesity
**Abstract:**	Background: The growing burden of asthma in low- and middle-income countries has been linked to urbanisation and lifestyle changes. However, this burden has not been well characterised in adults. Therefore, we investigated the prevalence of self-reported diagnosed asthma and associated factors in urban and rural adults in Malawi, Southern Africa.<br>Methods: Within a cross-sectional population-based survey to determine the burden and risk factors for non-communicable diseases (NCDs) in the city of Lilongwe and rural Karonga district, we collected information on self-reported previously diagnosed asthma and asthma-related symptoms using an interviewer-led questionnaire. Other data collected included: demographic characteristics, socioeconomic status indicators, NCD comorbidities, environmental exposures, and anthropometric measurements. We used multivariable logistic regression models to explore factors associated with self-reported asthma adjusting for variables associated with the outcome in univariable analysis. Findings were corrected for multiple comparisons using the Bonferroni method.<br>Results: We analysed data from 30,483 adult participants (54.6% urban,45.4% rural and 61.9% female). A prior asthma diagnosis was reported in 5.1% of urban and 4.5% of rural participants. In urban females, being obese (>30 kg/m2) compared to normal weight (18.5–24.9 kg/m2) was associated with greater odds of asthma (OR=1.59, 95% CI [1.26– 2.01], p<0.001), after adjusting for confounders. We observed associations between previously diagnosed heart disease and asthma in female participants which remained significant in rural females after Bonferroni correction (OR=2.30,95%CI [1.32– 4.02], p=0.003). Among rural males, current smokers had reduced odds of diagnosed asthma (OR=0.46,95%CI [0.27–0.79], p=0.004) compared to those who had never smoked.<br>Conclusions: In Malawi the prevalence of self-reported diagnosed asthma was greatest in females and urban dwellers. Notably, our findings indicate relationships between excess body weight as well as comorbidities and diagnosed asthma in females. Future investigations using longitudinally collected data and clinical measurements of asthma are needed to better understand these associations.
**Additional Information:**	
**Question**	**Response**
**Financial Disclosure**	Funding for this study was from the Wellcome Trust (098610/Z/12/Z and 098610/B/12/A).

Powered by Editorial Manager® and ProduXion Manager® from Aries Systems Corporation

Enter a financial disclosure statement that describes the sources of funding for the work included in this submission and the role the funder(s) played. This includes grants and any commercial funding of the work or authors.<br><br>This statement will be typeset if the manuscript is accepted for publication.<br><br>*Please review the submission guidelines and the instructions link below for detailed requirements and guidance.*	The funder played no role in the study design, data collection and analysis, decision to publish, or preparation of the manuscript.
**Competing Interests**<br><br>On behalf of all authors, disclose any competing interests that could be perceived to bias this work.<br><br>This statement will be typeset if the manuscript is accepted for publication.<br><br>*Please review the instructions link below and PLOS Global Public Health's competing interests policy to determine what information must be disclosed at submission.*	Not applicable
**Data Availability**<br><br>Before publication, Authors are required to make fully available and without restriction all data underlying their findings. Please see our PLOS Data Policy page for detailed information on this policy.<br><br>A **Data Availability Statement**, detailing where the data can be accessed, is required at first submission. Insert your Data Availability Statement in the box below.	All data used in these analyses have been anonymised and made available for download via the Zenodo Repository (URL to be provided).

Please see the data reporting section of our submission guidelines for instructions on what you need to include in your Data Availability Statement.<br><br>This statement will be typeset if the manuscript is accepted for publication.<br><br>*PLOS allows rare exemptions to address legal and ethical concerns. If you have legal or ethical restrictions, please use the box below to detail these in full sentences for the Journal team to consider.*	

Powered by Editorial Manager® and ProduXion Manager® from Aries Systems Corporation

Manuscript

Click here to access/download;Manuscript;CLEAN Revised manuscript Amoah et al.docx

Factors associated with self-reported diagnosed asthma in urban and rural Malawi: observations from a population-based study of non-communicable diseases

Abena S. Amoah[1,2,#a*], Estelle McLean[1,2] Alison J. Price[1,2], Alemayehu Amberbir[3], Amelia C. Crampin[1,2,4]

1. Department of Population Health, Faculty of Epidemiology and Population Health, London School of Hygiene and Tropical Medicine, London, United Kingdom
2. Malawi Epidemiology and Intervention Research Unit, Malawi
3. University of Global Health Equity, Kigali, Rwanda
4. School of Health and Wellbeing, University of Glasgow, Glasgow, United Kingdom

#a. Current Address: Leiden University Center for Infectious Diseases, Leiden University Medical Center, Leiden, The Netherlands

*Corresponding Author: Abena S. Amoah

Email: a.s.amoah@lumc.nl

Key Words: Asthma, Epidemiology, Noncommunicable diseases, Obesity, Urbanization, Wheeze

Abstract

Background: The growing burden of asthma in low- and middle-income countries has been linked to urbanisation and lifestyle changes. However, this burden has not been well characterised in adults. Therefore, we investigated the prevalence of self-reported diagnosed asthma and associated factors in urban and rural adults in Malawi, Southern Africa.

Methods: Within a cross-sectional population-based survey to determine the burden and risk factors for non-communicable diseases (NCDs) in the city of Lilongwe and rural Karonga district, we collected information on self-reported previously diagnosed asthma and asthma-related symptoms using an interviewer-led questionnaire. Other data collected included: demographic characteristics, socioeconomic status indicators, NCD comorbidities, environmental exposures, and anthropometric measurements. We used multivariable logistic regression models to explore factors associated with self-reported asthma adjusting for variables associated with the outcome in univariable analysis. Findings were corrected for multiple comparisons using the Bonferroni method.

Results: We analysed data from 30,483 adult participants (54.6% urban,45.4% rural and 61.9% female). A prior asthma diagnosis was reported in 5.1% of urban and 4.5% of rural participants. In urban females, being obese (>30 kg/m^2) compared to normal weight (18.5–24.9 kg/m^2) was associated with greater odds of asthma (OR=1.59, 95% CI [1.26– 2.01], p<0.001), after adjusting for confounders. We observed associations between previously diagnosed heart disease and asthma in female participants which remained significant in rural females after Bonferroni correction (OR=2.30,95%CI [1.32– 4.02], p=0.003). Among rural males, current smokers had reduced odds of diagnosed asthma (OR=0.46,95%CI [0.27–0.79], p=0.004) compared to those who had never smoked.

Conclusions: In Malawi the prevalence of self-reported diagnosed asthma was greatest in females and urban dwellers. Notably, our findings indicate relationships between excess body weight as well as comorbidities and diagnosed asthma in females. Future investigations using longitudinally collected data and clinical measurements of asthma are needed to better understand these associations.

Introduction

Asthma is one of the most common chronic conditions [1] affecting an estimated 262 million people worldwide and in 2019, resulted in 455,000 premature deaths [2]. Most asthma-related deaths occur in low- and middle-income countries (LMICs) where health-care resources are constrained, and the condition remains under-diagnosed and under-treated [2].

Asthma is made up of multiple phenotypes and represents complex interactions between genetic and environmental determinants [3]. Factors associated with urbanisation such as greater exposure to environmental pollutants [4], changes in diet [5], a greater body mass index [6, 7] and indoor allergen exposure [8], have all been linked to the rising prevalence in asthma worldwide. In children, the global burden of asthma and associated risk factors have been relatively well-characterised [9-11]. Much less is known about asthma in adults, particularly in LMICs. From observations made primarily in high income countries (HICs), adult-onset asthma is largely non-allergic [12], more common in females [13], associated with an accelerated decline in lung function [12] and linked to obesity [14-16]. The few studies conducted on asthma in LMICs do indicate marked urban-rural variations in asthma burden [17-21]. Initiatives such as the Burden of Obstructive Lung Disease (BOLD) study which collected country-specific data to characterise lung disease in adults worldwide [22, 23] have been addressing the evidence gap between HICs and LMICs. However, diagnostic challenges exist and in many LMICs where the availability of diagnostic tools for asthma are limited, diagnosis and management of the condition rely largely on clinical observations by health-care professionals and reported symptoms thus resulting in under-diagnosis and under-treatment [24].

In Malawi, a low-income country in sub-Saharan Africa, asthma is commonly diagnosed in primary health-care settings [25]. Most published studies from Malawi have been hospital-based and focused on the diagnosis and management of multiple respiratory conditions including asthma [26-28]. Recently, several population-based studies conducted in the Southern region of Malawi investigated lung function, respiratory symptoms, and the burden of noncommunicable lung disease. [29-32]

Although not focused on asthma, some of these studies established associations between biomass smoke exposure, smoking, sex and respiratory symptoms in adults in Southern Malawi [29, 30].

The aim of our current study was to investigate the prevalence of self-reported diagnosed asthma in adults. Additionally, we sought to explore factors associated with asthma, including demographic characteristics, anthropometric measurements, socioeconomic status indicators, NCD comorbidities and environmental smoke exposures.

Methods

Study Design

Between May 2013 and April 2017, the Malawi Epidemiology and Intervention Research Unit (previously known as Karonga Prevention Study) conducted a large population-based cross-sectional investigation of the burden and risk factors for non-communicable diseases (NCDs) in urban and rural adults in Malawi. The design, population descriptions and detailed methodology for this study have been described elsewhere [33, 34]. Published findings from this investigation observed notable differences in the prevalence of NCD outcomes such as hypertension in urban versus rural area as well as in males versus females [34]. Within this large study, information was also collected on self-reported diagnosed asthma, asthma symptoms and factors potentially associated with asthma.

Study area and population

For our investigation, the rural study area was within the established Karonga Health and Demographic Surveillance Site (Karonga HDSS) [35] where approximately 50,000 individuals reside in a geographical area of 135km^2 in the southern part of Karonga District in the Northern Region of Malawi and are under continuous demographic surveillance. The Karonga HDSS population is predominantly rural with an economy based on subsistence farming, fishing and petty trading [35]. Our urban study area was in Lilongwe, the capital city of Malawi, which is in the Central Region of the country. The study area in Lilongwe was an enumerated zone in a high-density residential area named Area 25 which has an estimated population of 107,316 individuals [36] of mixed socioeconomic status [33]. Enumeration involved collecting data on household membership, location details, and information on individuals ≥18 years [33]. Data collection for our study was between 16 May 2013 and 25 April 2017 in both study areas.

Data collection

Study questionnaire

Questions from the International Union against Tuberculosis and Lung Disease (IUALTD) bronchial symptoms questionnaire [37] were administered to participants along with questions on demographic parameters, socioeconomic indicators, medical history as well as lifestyle factors. These measurements have been used elsewhere in Africa [38].

The specific questions related to asthma and its symptoms were:

1. *Have you ever been diagnosed with asthma, asthmatic bronchitis or allergic bronchitis by a doctor or other health professional? If yes, what age were you when first diagnosed?*
2. *Are you on regular medication for asthma? If yes, name of medication* ____________
3. *Have you had wheezing or whistling in your chest at any time in the past year?* `
4. *In the last year, have you ever had an attack of wheezing or whistling that has made you feel short of breath?*

The study questionnaire was developed in English, translated into Chitumbuka (for the rural area) and Chichewa (for the urban area). It was then independently back translated and tested to ensure understanding and acceptability before implementation. Questionnaires were administered by trained interviewers using electronic tablet computers.

Questions on potential risk factors for asthma such as age, area of residence (urban or rural), indicators of socio-economic position including education level, occupation, household income and ownership of certain possessions, physical activity levels, NCD comorbidities and environmental exposures such as cigarette smoke, firewood smoke exposure and water source were included.

For these analyses, we categorised education based on Malawi educational levels ranging from no formal education, primary school (Standard 1-5 and Standard 6-8), secondary school and tertiary education. Physical activity was based on minutes spent on physical activity according to the World

Health Organisation (WHO)'s guidelines [39] and three categories were generated (low, moderate and high physical activity). We generated a wealth score based on possessions (electricity, lamp, mobile phone etc.) and an income score based on reported income. The NCD comorbidities of interest were previously diagnosed diabetes mellitus, heart disease, high blood pressure and stroke as reported by study participants. For smoke exposures, 'smoking status' was based on individual smoking history while 'smoker in household' gauged second-hand smoke exposure at home. We combined information on firewood use for cooking and whether cooking areas were ventilated or non-ventilated to generate a firewood exposure variable.

Anthropometric measurements

For each study participant, we determined weight and height measurements using a calibrated scale and portable stadiometer respectively. Body Mass Index (BMI) was calculated by dividing weight (kg) by height (m) squared and was categorised according to the WHO's BMI classification for adults: underweight (less than 18.5kg/m^2), normal weight (18.5-24.9kg/m^2), overweight (25.0-29.9kg/m^2) and obese (>30.0kg/m^2) [40].

Data management and quality assurance

Data was predominantly captured electronically via protected tablet computers using forms pre-programmed in Open Data Kit (https://getodk.org/) which were uploaded each day to a database housed in secure central server at each research site. Data quality was assured through rigorous training of Interviewers and the use of an interviewer's guide to ensure uniformity of data collection. Pre-programmed data checks were performed in the MEIRU data offices to identify any discrepancies which were addressed immediately. Uploaded data were also regularly extracted and checked for scientific validity. Data management processes were strictly controlled through adherence to set protocols and a central data dictionary [41].

Informed consent and ethical approval

We provided a full explanation of the study objectives and procedures to potential participants prior to seeking individual written consent. Ethical approval for the study was granted by the Malawi National Health Sciences Research Committee (protocol number #1072) and the London School of Hygiene & Tropical Medicine Ethics Committee (protocol number #6303).

Statistical analysis

Our primary outcome was self-reported diagnosed asthma. A secondary outcome was reported wheeze in the past 12 months. We also considered other asthma-related outcomes for descriptive analysis. We investigated differences in baseline characteristics stratified by sex and urban-rural area using Pearson's $\chi 2$ tests (for categorical variables) and Mann-Whitney U tests (for continuous variables). In preliminary analysis, we evaluated the extent to which associations between our covariates and outcome of asthma varied by sex and urban-rural area. To this end, we generated models fitted with an interaction term between sex and each covariate as well as separate models with interaction terms for urban-rural area. Our exploration showed evidence that the association between some sociodemographic indicators (for example, age-group, BMI, education, and work status) varied by sex and urban-rural area ($p<0.05$). Therefore, our logistic regression models were generated stratified by sex and urban-rural area. In univariable logistic regression analysis, associations between factors of interest and study outcomes (asthma and wheeze in the past 12 months) were examined. A two-sided p-value threshold of <0.100 was used to determine factors for inclusion in multivariable analysis. Final models were adjusted for all factors found to be significant in univariable analysis. To account for the effect of multiple testing, we applied a Bonferroni correction where alpha of 5% was divided by the number variables in the final logistic regression model [42]. Therefore, a corrected alpha of 0.4% ($p=0.004$) was considered as the threshold for statistically significance for our multivariable regression analyses. All analyses were conducted using STATA version 17 software (StataCorp, College Station, Texas).

Results

Characteristics of the study population

A total of 30,483 participants (54.6% urban, 45.4% rural and 61.9% female) were included in the analyses. Table 1 shows characteristics of study participants stratified by sex and urban-rural area. According to WHO BMI classifications, 18.0 % of urban females were obese (30.0 kg/m^2) compared to 7.7% % of rural females. By contrast, 3.9% of urban males were classified as obese and only 1.0% of rural males. Most individuals in all groups reported a physical activity rating categorized as ‘high’ according to the WHO physical activity standards.

Table 1. Characteristics of study population stratified by sex and urban-rural area

FACTOR	Females (N=18,855)				Males (N=11,628)			
	LILONGWE – URBAN	KARONGA - RURAL	TOTAL	p-value	LILONGWE – URBAN	KARONGA - RURAL	TOTAL	p-value
**Age group (years)**								
18-29	5,581 (51.4)	2,952 (36.9)	8,533 (45.3)	**<0.001**	2,944 (50.8)	2,224 (38.2)	5,168 (44.4)	**<0.001**
30-39	3,027 (27.9)	1,980 (24.8)	5,007 (26.6)		1,417 (24.4)	1,425 (24.4)	2,842 (24.4)	
40-49	1,140 (10.5)	1,232 (15.4)	2,372 (12.6)		695 (12.0)	905 (15.5)	1,600 (13.8)	
50-59	623 (5.7)	835 (10.4)	1,458 (7.7)		368 (6.4)	576 (9.9)	944 (8.1)	
60-69	317 (2.9)	522 (6.5)	839 (4.5)		242 (4.2)	316 (5.4)	558 (4.8)	
70+	171 (1.6)	474 (5.9)	645 (3.4)		132 (2.3)	384 (6.6)	516 (4.4)	
**Body Mass Index (kg/m2)**								
Underweight (<18.5)	418 (4.1)	529 (7.1)	947 (5.3)	**<0.001**	410 (7.1)	564 (9.7)	974 (8.4)	**<0.001**
Normal (18.5-24.9)	5298 (51.5)	4900 (65.6)	10198 (57.5)		4307 (74.3)	4729 (81.3)	9036 (77.8)	
Overweight (25.0-29.9)	2723 (26.5)	1465 (19.6)	4188 (23.6)		855 (14.8)	463 (8.0)	1318 (11.4)	
Obese(>30.0)	1846 (18.0)	572 (7.7)	2418 (13.6)		224 (3.9)	59 (1.0)	283 (2.4)	
**Physical activity rating**								
Low	178 (1.6)	148 (1.9)	326 (1.7)	**0.001**	240 (4.1)	145 (2.5)	385 (3.3)	**<0.001**
Moderate	284 (2.6)	278 (3.5)	562 (3.0)		775 (13.4)	624 (10.7)	1,399 (12.0)	
High	10,398 (95.8)	7,569 (94.7)	17,967 (95.3)		4,783 (82.5)	5,061 (86.8)	9,844 (84.7)	
**Household income, median (IQR)**	7 (5 - 9)	4 (2 - 5)	5 (3 - 8)	**<0.001**	7 (5 - 9)	4 (3 - 6)	6 (4 - 8)	**<0.001**
**Household asset index, median (IQR)**	7 (5 - 9)	4 (2 - 6)	6 (3 - 8)	**<0.001**	7 (5 - 9)	4 (3 - 7)	6 (3 - 8)	**<0.001**
**Education**								
No formal	505 (4.7)	496 (6.2)	1,001 (5.3)	**<0.001**	78 (1.4)	99 (1.7)	177 (1.5)	**<0.001**
Primary: Standard 1-5	1,059 (9.8)	1,366 (17.1)	2,425 (12.9)		272 (4.7)	634 (10.9)	906 (7.8)	
Primary: Standard 6-8	2,486 (22.9)	4,065 (50.8)	6,551 (34.7)		814 (14.0)	2,407 (41.3)	3,221 (27.7)	
Secondary	5,357 (49.3)	1,990 (24.9)	7,347 (39.0)		3,333 (57.5)	2,515 (43.1)	5,848 (50.3)	
Tertiary	1,453 (13.4)	78 (1.0)	1,531 (8.1)		1,301 (22.4)	175 (3.0)	1,476 (12.7)	
**Work Status**								
Not working	2,525 (23.3)	730 (9.1)	3,255 (17.3)	**<0.001**	2,085 (36.0)	1,012 (17.4)	3,097 (26.6)	**<0.001**
Housework	4,619 (42.5)	763 (9.5)	5,382 (28.5)		364 (6.3)	70 (1.2)	434 (3.7)	
Farming/fishing	57 (0.5)	5,266 (65.9)	5,323 (28.2)		30 (0.5)	3,369 (57.8)	3,399 (29.2)	
Self-employed	1,957 (18.0)	1,040 (13.0)	2,997 (15.9)		1,163 (20.1)	852 (14.6)	2,015 (17.3)	
Employed	1,702 (15.7)	196 (2.5)	1,898 (10.1)		2,156 (37.2)	527 (9.0)	2,683 (23.1)	
**Diagnosed Diabetes Mellitus**								
No	10704 (98.6)	7926 (99.1)	18630 (98.8)	**<0.001**	5706 (98.4)	5789 (99.3)	11495 (98.9)	**<0.001**
Yes	156 (1.4)	69 (0.9)	225 (1.2)		92 (1.6)	41 (0.7)	133 (1.1)	
**Diagnosed Heart Disease**								
No	10578 (97.4)	7825 (97.9)	18403 (97.6)	**0.037**	5739 (99.0)	5784 (99.2)	11523 (99.1)	0.193
Yes	282 (2.6)	170 (2.1)	452 (2.4)		59 (1.0)	46 (0.8)	105 (0.9)	
**Diagnosed High Blood Pressure**								
No	9448 (87.0)	7339 (91.8)	16787 (89.0)	**<0.001**	5337 (92.1)	5606 (96.2)	10943 (94.1)	**<0.001**
Yes	1412 (13.0)	656 (8.2)	2068 (11.0)		461 (8.0)	224 (3.8)	685 (5.9)	
**Diagnosed Stroke**								
No	10784 (99.3)	7941 (99.3)	18725 (99.3)	0.841	5750 (99.2)	5782 (99.2)	11532 (99.2)	0.978
Yes	76 (0.7)	54 (0.7)	130 (0.7)		48 (0.8)	48 (0.8)	96 (0.8)	
**Piped water at home**								
No	6,906 (63.6)	7,404 (92.6)	14,310 (75.9)	**<0.001**	3,404 (58.7)	5,345 (91.7)	8,749 (75.2)	**<0.001**
Yes	3,954 (36.4)	591 (7.4)	4,545 (24.1)		2,394 (41.3)	485 (8.3)	2,879 (24.8)	
**Smoking Status**								
Never smoked	10,790 (99.4)	7,971 (99.7)	18,761 (99.5)	**0.001**	4,850 (83.7)	4,777 (81.9)	9,627 (82.8)	**0.001**
Former smoker (stopped more than 6 months ago)	43 (0.4)	10 (0.1)	53 (0.3)		442 (7.6)	262 (4.5)	704 (6.1)	
Current (in last 6 months)	27 (0.3)	14 (0.2)	41 (0.2)		506 (8.7)	791 (13.6)	1,297 (11.2)	

**Smoker in household**								
No	9,722 (89.5)	6,755 (84.5)	16,477 (87.4)	**<0.001**	5,305 (91.5)	5,249 (90.0)	10,554 (90.8)	**0.006**
Yes	1,138 (10.5)	1,240 (15.5)	2,378 (12.6)		493 (8.5)	581 (10.0)	1,074 (9.2)	
**Firewood smoke exposure**								
no/former/little exposure	1003 (9.4)	266 (3.4)	1269 (6.8)	**<0.001**	1240 (21.8)	2392 (43.6)	3632 (32.5)	**<0.001**
exposed, ventilated cooking area	8474 (79.3)	7541 (96.0)	16015 (86.3)		3874 (68.1)	3073 (56.0)	6947 (62.1)	
exposed non-ventilated cooking area	1216 (11.4)	49 (0.6)	1265 (6.8)		576 (10.1)	24 (0.4)	600 (5.4)	

- P-values were calculated by using Pearson χ2 test with 1 degree of freedom or Mann Whitney U test for continuous variables.
- Values in boldface indicate $P<0.05$
- **Missing data for:**
  - **Age group:** 1 urban female participant
  - **Body Mass Index:** 544 rural and 577 urban participants
  - **Firewood smoke exposure:** 490 rural and 275 urban participants

Compared to rural participants, urban males and females had significantly higher values when it came to markers of socio-economic position (median household income, median household possessions and proportion who completed tertiary education or were self-employed / employed).

With regards to NCD comorbidities, diagnosed high blood pressure was significantly greater in urban females (13.0%) compared to rural females (8.2%). The same pattern was observed for urban versus rural males. Overall, the proportion of participants reporting NCD comorbidities was relatively low (<3%).

Over 35% of urban participants of both sexes reported having piped water as a main source of household water compared to under 10% of rural participants. Current smoking in our study population was low overall but higher in males (11.2%) compared to females (0.2%). More rural females (15.5%) reported being exposed to household second-hand smoke compared to urban females (10.5%). When it came to firewood exposure, more urban females (11.4%) reported firewood smoke exposure in a non-ventilated cooking area compared to rural females (0.6%). The same pattern was observed for urban males (10.1%) compared to rural males (0.4%) for this factor.

Prevalence of asthma and related symptoms

Diagnosed asthma was reported in 5.1% of urban and 4.5% of rural participants. As shown in Table 2, a significantly higher proportion of urban females (5.6%) reported diagnosed asthma compared to rural females (4.5%) (p=0.001) but no difference was observed for males (p=0.871). Median age of asthma diagnosis was higher in rural females (17.0 years, IQR [6.5 - 28.0]) compared to urban females (14.0 years, IQR [5.0 - 23.0] and much lower in both male groups. Only 2.1% of participants reported regular use of asthma medication (for example, aminophylline, salbutamol, prednisone, or inhalers). As shown in Fig 1, 71.9% (1067/1484) of those with diagnosed asthma did not report having symptoms of 'wheeze in the past 12 months' or 'wheeze in the past 12 months plus shortness of breath'.

Table 2. Asthma outcomes and symptoms stratified by sex and urban-rural area

Asthma Outcomes	Females (N=18,855)				Males (N=11,628)			
	LILONGWE – URBAN	KARONGA - RURAL	TOTAL	p-value	LILONGWE – URBAN	KARONGA – RURAL	TOTAL	p-value
Diagnosed asthma (Yes)	611 (5.6)	362 (4.5)	973 (5.2)	**0.001**	253 (4.4)	258 (4.4)	511 (4.4)	0.871
Age of asthma diagnosis (years), median (IQR) subset	14.0 (5.0 – 23.0)	17.0 (6.5 – 28.0)	15.0 (5.0 – 25.0)	**<0.001**	11.0 (4.0 – 19.8)	12.0 (5.0 – 25.0)	12.0 (5.0 – 22.0)	0.073
Diagnosed asthma with medication (Yes)	256 (2.4)	186 (2.3)	442 (2.3)	0.890	82 (1.4)	101 (1.7)	183 (1.6)	0.168
Wheeze in the past 12 months (Yes)	371 (3.4)	257 (3.2)	628 (3.3)	0.446	168 (2.9)	193 (3.3)	361 (3.1)	0.199
Wheeze in the past 12 months plus shortness of breath (Yes)	501 (4.6)	262 (3.3)	763 (4.1)	**<0.001**	237 (4.1)	177 (3.0)	414 (3.6)	**0.002**
Asthma plus Wheeze in the past 12 months (Yes)	135 (1.2)	101 (1.3)	236 (1.3)	0.902	46 (0.8)	66 (1.1)	112 (1.0)	0.062
Asthma diagnosed < 18 years (Yes)	340 (59.9)	147 (50.2)	487 (56.6)	**0.007**	166 (71.6)	135 (61.6)	301 (66.7)	**0.026**

- P-values were calculated by using Pearson χ2 test with 1 degree of freedom or Mann Whitney U test for continuous variables.
- Values in boldface indicate P<0.05

Fig 1. Overlap between 'Diagnosed asthma'' and related symptoms

Fig 1. Figure is a proportional Venn diagram showing the overlap between the outcomes 'Diagnosed asthma', 'Wheeze in the past 12 months' and 'Wheeze in the past 12 months plus shortness of breath'. The area representing each variable is proportional to the number of people reporting this outcome.

Poor overlap was also seen with the outcome 'diagnosed asthma with medication use' where 59.8% (374/625) did not report having 'wheeze in the past 12 months' or 'wheeze in the past 12 months plus shortness of breath (S1 Fig).

Factors associated with a self-reported asthma diagnosis

S1 Table shows the results of univariable logistic regression analysis of potential factors associated with a self-reported diagnosed asthma stratified by sex and urban-rural area. The factors found to be associated with asthma (p<0.100) were age-group, BMI, physical activity rating, household income, household asset index, education, work status, diagnosed heart disease, diagnosed blood pressure, piped water at home, smoking status, and firewood smoke exposure.

Demographic, anthropometric, and socioeconomic factors

As shown in Table 3, after adjusting for other factors, and considering a Bonferroni corrected significance level of p=0.004, in urban females only, being obese was associated with increased odds of diagnosed asthma compared to being normal weight (OR = 1.59, 95%CI 1.26 – 2.01, p<0.001). In rural females, a secondary school education (OR = 2.10, 95%CI 1.35 – 3.27, p=0.001) was positively associated with diagnosed asthma compared to completing primary Standard 1-5. By contrast, urban females completing primary Standard 6-8 had reduced odds of reported diagnosed asthma (OR = 0.58, 95%CI 0.41 – 0.81, p=0.001) compared to those completing primary Standard 1-5. No significant associated were observed for education and asthma when it came to the male groups.

Table 3. Multivariable analysis - Factors associated with diagnosed asthma stratified by sex & urban-rural area

Factors	Females				Males			
	LILONGWE - URBAN		KARONGA - RURAL		LILONGWE - URBAN		KARONGA - RURAL	
	Adjusted Odds ratio (95%CI)	p-value	Adjusted Odds ratio (95%CI)	p-value	Adjusted Odds ratio (95%CI)	p-value	Adjusted Odds ratio (95%CI)	p-value
**Age group**								
18-29	1.00	-	1.00	-	1.00	-	1.00	-
30-39	1.14 (0.92 - 1.41)	0.225	1.43 (1.06 - 1.92)	0.020	0.99 (0.69 - 1.43)	0.958	1.14 (0.81 - 1.61)	0.439
40-49	0.94 (0.69 - 1.28)	0.685	1.34 (0.94 - 1.91)	0.102	1.03 (0.66 - 1.62)	0.883	0.63 (0.39 - 1.00)	0.051
50-59	1.42 (0.98 - 2.04)	0.063	1.05 (0.67 - 1.65)	0.843	0.75 (0.40 - 1.39)	0.364	1.02 (0.62 - 1.67)	0.945
60-69	1.17 (0.69 - 1.99)	0.556	1.92 (1.17 - 3.15)	0.010	0.59 (0.27 - 1.31)	0.199	0.85 (0.43 - 1.68)	0.637
70+	1.51 (0.74 - 3.08)	0.259	0.84 (0.39 - 1.83)	0.663	0.56 (0.19 - 1.69)	0.307	1.13 (0.58 - 2.19)	0.727
**BMI (WHO categories)**								
Underweight (<18.5)	0.93 (0.57 - 1.52)	0.775	1.26 (0.83 - 1.92)	0.276	1.08 (0.66 - 1.76)	0.750	1.52 (1.02 - 2.27)	0.040
Normal(18.5-24.9)	1.00	-	1.00	-	1.00	-	1.00	-
Overweight(25.0-29.9)	1.30 (1.05 - 1.60)	0.014	0.91 (0.68 - 1.24)	0.561	0.91 (0.62 - 1.34)	0.647	0.93 (0.57 - 1.53)	0.786
Obese(>30)	**1.59 (1.26 - 2.01)**	**<0.001**	1.20 (0.81 - 1.80)	0.367	0.71 (0.35 - 1.47)	0.359	1.05 (0.32 - 3.49)	0.933
**Physical activity rating**								
Low	1.00	-	1.00	-	1.00	-	1.00	-
Moderate	0.71 (0.33 - 1.51)	0.367	1.01 (0.29 - 3.51)	0.987	1.10 (0.56 - 2.15)	0.783	0.31 (0.14 - 0.72)	0.007
High	0.73 (0.40 - 1.36)	0.321	1.56 (0.52 - 4.73)	0.430	0.74 (0.39 - 1.41)	0.368	0.56 (0.27 - 1.12)	0.102
**Household income**	1.00 (0.97 - 1.04)	0.800	1.00 (0.95 - 1.06)	0.966	1.03 (0.97 - 1.09)	0.322	1.08 (1.01 - 1.15)	0.017
**Household asset index**	1.04 (0.99 - 1.08)	0.094	0.99 (0.94 - 1.04)	0.667	1.05 (0.98 - 1.12)	0.149	1.02 (0.96 - 1.08)	0.528
**Education**								
No formal	0.52 (0.30 - 0.91)	0.022	1.19 (0.64 - 2.22)	0.584	4.07 (0.98 - 16.82)	0.053	0.50 (0.07 - 3.88)	0.510
Primary: Standard 1-5	1.00	-	1.00	-	1.00	-	1.00	-
Primary: Standard 6-8	**0.58 (0.41 - 0.81)**	**0.001**	1.68 (1.14 - 2.48)	0.009	2.73 (0.96 - 7.82)	0.061	1.48 (0.84 - 2.60)	0.170
Secondary	0.93 (0.69 - 1.26)	0.648	**2.10 (1.35 - 3.27)**	**0.001**	2.43 (0.87 - 6.75)	0.089	1.74 (0.98 - 3.07)	0.058
Tertiary	1.44 (1.00 - 2.08)	0.048	2.69 (0.99 - 7.32)	0.053	3.39 (1.19 - 9.67)	0.023	1.86 (0.80 - 4.32)	0.150
**Work Status**								
Not working	1.00	-	1.00	-	1.00	-	1.00	-
Housework	1.09 (0.85 - 1.40)	0.486	0.76 (0.45 - 1.28)	0.294	1.08 (0.62 - 1.89)	0.790	2.41 (1.03 - 5.66)	0.043
Farming/fishing	0.75 (0.18 - 3.20)	0.703	0.77 (0.51 - 1.17)	0.225	0.86 (0.11 - 6.57)	0.885	0.93 (0.64 - 1.36)	0.703
Self-employed	1.32 (1.00 - 1.76)	0.054	0.85 (0.53 - 1.38)	0.518	1.13 (0.75 - 1.70)	0.548	0.77 (0.47 - 1.25)	0.285
Employed	1.19 (0.90 - 1.58)	0.228	1.23 (0.60 - 2.52)	0.572	0.76 (0.52 - 1.10)	0.145	1.16 (0.69 - 1.95)	0.573
**Diagnosed Heart Disease**								
No	1.00	-	1.00	-	1.00	-	1.00	-
Yes	1.76 (1.16 - 2.68)	0.008	**2.30 (1.32 - 4.02)**	**0.003**	1.17 (0.36 - 3.84)	0.792	0.96 (0.23 - 4.04)	0.952
**Diagnosed High Blood Pressure**								
No	1.00	-	1.00	-	1.00	-	1.00	-
Yes	0.93 (0.72 - 1.21)	0.611	1.22 (0.80 - 1.85)	0.357	1.16 (0.70 - 1.92)	0.567	0.98 (0.49 - 1.95)	0.951
**Piped water at home**								
No	1.00	-	1.00	-	1.00	-	1.00	-
Yes	1.05 (0.85 - 1.29)	0.658	1.23 (0.84 - 1.82)	0.286	0.92 (0.67 - 1.26)	0.601	1.18 (0.78 - 1.81)	0.431
**Smoking Status**								
Never smoked	1.00		1.00		1.00		1.00	
Former smoker (stopped more than 6 months ago)	4.17 (1.94 - 8.94)	<0.001	*no output*	-	1.06 (0.66 - 1.70)	0.822	1.09 (0.59 - 2.00)	0.787
Current (in last 6 months)	0.81 (0.11 - 6.09)	0.839	*no output*	-	0.85 (0.52 - 1.41)	0.534	**0.46 (0.27 - 0.79)**	**0.004**
**Firewood smoke exposure**								
No/former/little exposure	1.00	-	1.00	-	1.00	-	1.00	-
Exposed, ventilated cooking area	1.41 (1.04 - 1.90)	0.027	0.65 (0.35 - 1.23)	0.189	0.81 (0.60 - 1.11)	0.189	1.00 (0.76 - 1.32)	0.993
Exposed, non-ventilated cooking area	1.07 (0.72 - 1.60)	0.733	0.72 (0.15 - 3.43)	0.684	0.64 (0.37 - 1.11)	0.113	0.96 (0.13 - 7.27)	0.966

- **Final model adjusted for**: age-group, BMI, physical activity rating, household income, household asset index, education, work status, diagnosed heart disease, diagnosed high blood pressure, piped water at home, smoking status, and firewood smoke exposure
- Adjusted ORs, 95% CIs and p-values in boldface indicate model results where $p \leq 0.004$ (significance level threshold after Bonferroni correction)
- *no output*: models with no output for this level due to small numbers

Other NCD comorbidities

We observed that previously diagnosed heart disease (yes versus no) was positively associated diagnosed asthma in rural females (OR=2.30, 95%CI 1.32 – 4.02, p=0.003). A similar observation was made in urban females, but this was not significant if we considered multiple testing. No association was seen in either male group with regards to any NCD comorbidity.

Environmental factors – water source, smoke exposure

In urban females, being a former smoker compared to having never smoked, was associated with diagnosed asthma (OR=4.17, 95%CI [1.94 – 8.94], p<0.001). However, the number of females who were smokers/former smokers in this population was relatively small. In rural males only, being a current smoker (in the last 6 months) compared to having never smoked had an inverse association with asthma diagnosis (OR = 0.46, 95% CI [0.27 – 0.79], p=0.004).

Factors associated with wheeze in the past 12 months

S2 Table shows the results of univariable logistic regression analysis of potential factors associated with reported wheeze in the past 12 months stratified by sex and urban-rural area. All factors found to be associated with wheeze (p<0.100) were explored further in multivariable analysis. The factors included in the final multivariable model were age-group, BMI, physical activity rating, household income, household asset index, education, work status, diagnosed heart disease, diagnosed blood pressure, diagnosed stroke, piped water at home, smoking status, and firewood smoke exposure.

Demographic, anthropometric, and socioeconomic factors

No association was observed between being obese (compared to normal weight) and 'wheeze in the past 12 months' in urban females (Table 4). Increasing household income as a linear variable was significantly associated with wheeze in the past 12 months among rural males (OR=1.13, 95%CI 1.06 – 1.22, p<0.001). When it came to household possessions, increasing 'household asset index' was positively associated with 'wheeze in the past 12 months' in urban females only (OR=1.09, 95%CI 1.03 – 1.15, p=0.003). Unlike with self-diagnosed asthma, educational status was not associated with wheeze in the past 12 months.

Table 4. Multivariable analysis -Factors associated with 'Wheeze in the past 12 months' stratified by sex & urban-rural area

Factors	Female				Male			
	LILONGWE – URBAN		KARONGA - RURAL		LILONGWE - URBAN		KARONGA - RURAL	
	Adjusted Odds ratio (95%CI)	p-value	Adjusted Odds ratio (95%CI)	p-value	Adjusted Odds ratio (95%CI)	p-value	Adjusted Odds ratio (95%CI)	p-value
**Age group**								
18-29	1.00	-	1.00	-	1.00	-	1.00	-
30-39	0.98 (0.74 - 1.29)	0.874	1.32 (0.90 - 1.93)	0.157	1.14 (0.72 - 1.79)	0.575	0.98 (0.65 - 1.48)	0.933
40-49	1.00 (0.68 - 1.47)	0.992	1.56 (1.03 - 2.36)	0.037	1.17 (0.67 - 2.05)	0.581	1.22 (0.77 - 1.92)	0.398
50-59	1.35 (0.87 - 2.08)	0.180	1.41 (0.87 - 2.30)	0.165	1.56 (0.82 - 2.96)	0.175	0.83 (0.44 - 1.56)	0.567
60-69	0.61 (0.30 - 1.22)	0.160	1.71 (0.98 - 3.00)	0.060	1.56 (0.71 - 3.43)	0.264	1.55 (0.79 - 3.01)	0.200
70+	1.01 (0.43 - 2.36)	0.982	0.63 (0.28 - 1.42)	0.262	1.57 (0.55 - 4.53)	0.401	1.34 (0.64 - 2.83)	0.439
**BMI (WHO categories)**								
Underweight (<18.5)	1.06 (0.60 - 1.90)	0.832	1.18 (0.71 - 1.96)	0.528	1.25 (0.72 - 2.18)	0.422	1.25 (0.77 - 2.03)	0.358
Normal(18.5-24.9)	1.00	-	1.00	-	1.00	-	1.00	-
Overweight(25.0-29.9)	1.25 (0.96 - 1.62)	0.094	1.00 (0.71 - 1.40)	0.991	0.65 (0.39 - 1.09)	0.104	1.45 (0.89 - 2.34)	0.134
Obese(>30)	1.15 (0.85 - 1.57)	0.358	1.19 (0.76 - 1.85)	0.445	0.58 (0.22 - 1.51)	0.266	2.11 (0.73 - 6.15)	0.169
**Physical activity rating**								
Low	1.00	-	1.00	-	1.00	-	1.00	-
Moderate	1.77 (0.76 - 4.12)	0.189	0.80 (0.27 - 2.34)	0.680	2.61 (0.97 - 7.01)	0.058	0.42 (0.17 - 1.07)	0.068
High	0.81 (0.37 - 1.75)	0.589	0.82 (0.31 - 2.18)	0.695	1.76 (0.66 - 4.65)	0.257	0.64 (0.29 - 1.44)	0.284
**Household income**	1.02 (0.98 - 1.07)	0.303	1.03 (0.96 - 1.09)	0.434	1.08 (1.00 - 1.16)	0.045	**1.13 (1.06 - 1.22)**	**<0.001**
**Household asset index**	**1.09 (1.03 - 1.15)**	**0.003**	1.02 (0.97 - 1.08)	0.392	1.01 (0.94 - 1.09)	0.792	0.96 (0.89 - 1.02)	0.184
**Education**								
No formal	0.69 (0.37 - 1.30)	0.256	1.14 (0.62 - 2.06)	0.677	*no output*	-	1.11 (0.25 - 5.03)	0.892
Primary: Standard 1-5	1.00	-	1.00	-	1.00	-	1.00	-
Primary: Standard 6-8	0.64 (0.43 - 0.96)	0.030	0.98 (0.66 - 1.44)	0.905	1.26 (0.57 - 2.76)	0.570	1.25 (0.68 - 2.29)	0.472
Secondary	0.73 (0.50 - 1.06)	0.101	1.07 (0.66 - 1.72)	0.782	0.85 (0.40 - 1.82)	0.680	1.59 (0.86 - 2.93)	0.138
Tertiary	0.85 (0.54 - 1.35)	0.489	1.67 (0.57 - 4.93)	0.349	0.98 (0.43 - 2.23)	0.957	0.88 (0.30 - 2.58)	0.821
**Work Status**								
Not working								
Housework	0.79 (0.58 - 1.08)	0.143	0.47 (0.24 - 0.91)	0.025	0.16 (0.04 - 0.65)	0.011	0.85 (0.20 - 3.69)	0.829
Farming/fishing	3.12 (1.23 - 7.90)	0.016	0.66 (0.41 - 1.06)	0.086	0.96 (0.12 - 7.40)	0.965	0.96 (0.61 - 1.49)	0.841
Self-employed	0.98 (0.69 - 1.39)	0.917	0.81 (0.46 - 1.41)	0.455	0.91 (0.56 - 1.47)	0.690	0.85 (0.49 - 1.47)	0.559
Employed	0.84 (0.59 - 1.20)	0.338	1.26 (0.56 - 2.82)	0.579	0.65 (0.42 - 1.01)	0.055	1.10 (0.60 - 2.01)	0.762
**Diagnosed heart disease**								
No	1.00	-	1.00	-	1.00	-	1.00	-
Yes	**4.84 (3.34 - 7.00)**	**<0.001**	**2.37 (1.32 - 4.26)**	**0.004**	**4.76 (2.15 - 10.56)**	**<0.001**	**5.59 (2.49 - 12.57)**	**<0.001**
**Diagnosed High Blood Pressure**								
No	1.00	-	1.00	-	1.00	-	1.00	-
Yes	1.30 (0.96 - 1.76)	0.096	**2.24 (1.50 - 3.33)**	**<0.001**	0.71 (0.37 - 1.36)	0.300	1.45 (0.73 - 2.90)	0.287
**Diagnosed Stroke**								
No	1.00	-	1.00	-	1.00	-	1.00	-
Yes	0.82 (0.28 - 2.39)	0.710	1.36 (0.40 - 4.66)	0.621	1.51 (0.34 - 6.68)	0.590	1.06 (0.24 - 4.72)	0.938
**Piped water at home**								
No	1.00	-	1.00	-	1.00	-	1.00	-
Yes	0.99 (0.77 - 1.29)	0.955	0.76 (0.45 - 1.27)	0.291	0.92 (0.63 - 1.35)	0.661	0.97 (0.58 - 1.64)	0.915
**Smoking status**								
Never smoked	1.00	-	1.00	-				
Former smoker (stopped more than 6 months ago)	1.06 (0.25 - 4.59)	0.933	*no output*	-	1.16 (0.67 - 2.01)	0.605	1.27 (0.67 - 2.43)	0.463
Current (in last 6 months)	1.43 (0.19 - 10.72)	0.731	8.00 (1.57 - 40.90)	0.012	1.23 (0.73 - 2.08)	0.428	1.21 (0.78 - 1.86)	0.401
**Firewood smoke exposure**								
No/former/little exposure	1.00	-	1.00	-	1.00	-	1.00	-

Exposed, ventilated cooking area	1.02 (0.73 - 1.44)	0.900	0.90 (0.44 - 1.84)	0.772	0.67 (0.46 - 0.97)	0.033	1.55 (1.12 - 2.14)	0.008
Exposed, non-ventilated cooking area	0.88 (0.55 - 1.39)	0.575	*no output*	-	1.06 (0.62 - 1.82)	0.820	No output	-

- **Final model adjusted for**: age-group, BMI, physical activity rating, household income, household asset index, education, work status, diagnosed heart disease, diagnosed high blood pressure, piped water at home, smoking status, and firewood smoke exposure.
- Adjusted ORs, 95% CIs and p-values in boldface indicate model results where $p \leq 0.004$ (significance level threshold after Bonferroni correction)
- *no output*: models with no output for this level due to small numbers

Other NCD comorbidities

In all groups, reported diagnosed heart disease was significantly associated with wheeze in the past 12 months. This was observed for rural females (OR=2.37, 95%CI 1.32 – 4.26, p=0.004), urban females (OR=4.84, 95%CI 3.34 – 7.00, p<0.001), urban males (OR=5.59, 95%CI 2.49 – 12.57, p<0.001) and rural males (OR=4.76, 95%CI 2.15 – 10.56, p<0.001). We also observed that a prior diagnosis of high blood pressure was associated with reported wheeze in the past 12 months in rural females only (OR=2.24, 95%CI 1.50 – 3.33, p<0.001).

Environmental factors – water source, smoke exposure

There was no strong evidence for an association between smoking status and wheeze outcomes. Exposure to firewood in ventilated cooking area was associated with reported wheeze in the past 12 months in rural males only but this did not reach significance after correcting for multiple testing (OR=1.55, 95%CI 1.12 – 2.14, p=0.008).

Discussion

In this large community-based survey of Malawian adults, we observed only a slightly higher prevalence of diagnosed asthma in urban compared to rural dwelling adults which is in contrasts to findings from elsewhere in Sub-Saharan Africa where asthma has been strongly linked to urbanisation and the adoption of a so-called western lifestyle [17-19, 21]. It is important to note that differences in underlying socioeconomic structures between African countries make direct comparisons challenging. For example, in Malawi, universally constrained access to healthcare and diagnostics may contribute to the lack of distinct urban-rural differences. In addition, the socioeconomic distribution of our study population may not be comparable to other studies that may have included urban dwellers of higher socioeconomic status with greater access to health-care [43, 44].

In our analysis we stratified our findings by both sex and urban-rural study area. The rationale for this was that published findings from our population cohort observed differences in the prevalence of disease outcomes such as hypertension according to urban-rural study area as well as sex [34]. Additionally, our preliminary analysis indicated that some sociodemographic indicators varied by both urban-rural area and sex.

We observed poor overlap between diagnosed asthma and asthma symptoms (reported wheeze in the past 12 months and wheeze in the past 12 months plus shortness of breath). This could indicate that most participants who had previously diagnosed with asthma were asymptomatic within the year prior to the questionnaire interview. It is also possible that reported wheeze may not be related to diagnosed asthma in our population. Additionally, other studies from Malawi have found a notable burden of abnormal lung function due to other chronic respiratory conditions such as chronic obstructive pulmonary disorder (COPD) [29, 30, 32].

The older age of asthma diagnosis in rural compared to urban dwellers that we noted could reflect a true higher prevalence of adult-onset asthma in the rural area. Nonetheless, it may also reflect some differences in health-seeking behaviours and improved access to healthcare in our rural area over

time. We also observed an association between self-reported diagnosed heart disease and asthma which is consistent with other investigations [45-47]. Similar to our own investigation, other studies report that the association is stronger among adult females compared to males [47]. Although the underlying mechanisms linking asthma and heart disease, particularly in females, remain unclear, an inflammatory cytokine milieu and oestrogen-modulated inflammation may play roles [48]. We also found an association between wheeze in the past 12 months and diagnosed heart disease in all groups. Due to lack of access to diagnostics within health centres in Malawi, an asthma and heart disease diagnoses are based mainly on clinical observations by health-care professionals and symptoms that may be similar. Hence some misclassification of these conditions is inevitable [49].

We did not observe a link between diagnosed high blood pressure and reported asthma in any group although a link between these two conditions has been established [50]. Interestingly, we did observe an association between diagnosed high blood pressure and reported wheeze in the past 12 months in rural females. However, this observation may not be linked to asthma given the lack of overlap between asthma and wheeze in our study.

Our observed strong association between obesity (BMI>30kg/m^2), and diagnosed asthma in urban females has been seen in other parts of the world [51, 52]. Although obesity is thought to affect the metabolic and immunological pathways that lead to asthma, specific mechanisms involved remain unclear [53]. One possible pathway is through the action of adipokines (cytokines produced by adipose tissue in obese individuals), which may affect the inflammatory process in the lungs and therefore asthma pathogenesis [53]. Nonetheless, the association between excess adiposity and asthma was not observed in urban males. This might reflect a female predisposition to adult-onset asthma, as observed in other parts of the world [54, 55] . However, we cannot rule out a potential misdiagnosis of asthma in obese individuals since both conditions are characterised by breathlessness [56].

In our study population, the relationship between socioeconomic status (SES) and diagnosed asthma was not consistent. This could reflect the limitations of our SES assessment tools or an indication that

a clear relationship did not exist. We did however observe that higher education appeared to be associated with asthma in urban females. It is possible that educational level was a better proxy for some of the socioeconomic factors associated with increased risk of asthma and this was apparent in urban females because of the high numbers of this group in our study. It could also reflect more health-seeking behaviour in urban females compared to their rural counterparts or better access to health-care facilities in an urban setting compared to a rural environment.

When we examined physical activity, we observed a negative association between moderate physical activity (compared to low activity) on diagnosed asthma in rural males but this did not reach significance after taking into account multiple testing.

Regarding smoking in our study, few females, especially in the rural area, were current smokers / former smokers which reflects the cultural norms in Malawi and has also been observed in other studies [29, 57]. However, we did observe that being a former smoker was associated with diagnosed asthma in urban females which may indicate smoking cessation at the time of diagnosis and therefore be an example of reverse causation [58]. At the same time, this finding could also reflect social desirability effects since individuals with asthma may underreport some behaviours such as smoking due to perceived or actual social disapproval [59]. However, our observations regarding smoking in female participants should be interpreted with caution given the small numbers. Our study findings also pointed to a 'protective effect' of current smoking on diagnosed asthma in rural males which again may indicate that diagnosed asthmatics were less likely to smoke or were potentially underreporting their smoking habits.

We did not observe any links between smoking and wheeze in our study. Other studies conducted in Malawi have looked at the effect of smoking on respiratory symptoms (cough, phlegm, wheeze or dyspnoea) and have observed a positive associations between being a current or ex-smoker and cough (without a cold) in urban adults in Blantyre, Malawi [29] as well as between 'smoking ever' and cough (but not wheeze) in rural adults in Chikwawa District [60].

Indoor solid biomass (crop waste, dung, firewood, charcoal etc) fuel smoke exposure has been associated with increased risk of respiratory conditions in LMICs especially [61, 62]. Although indoor biomass fuel exposure has been linked to COPD risk [63], the relationship with asthma is unclear [64]. In our investigation we found no significant association between exposure to firewood in a non-ventilated cooking area and diagnosed asthma. We indications of a positive relationship between this exposure and wheeze in the past 12 months among rural males. Given the poor overlap between asthma and wheeze in our investigation, this observation may not be related to asthma.

The strengths of our study include the use of a standard well-validated questionnaire and having a large sample size that enabled multivariable analysis as well as urban versus rural comparisons. Our study had some limitations including our reliance on self-reported diagnosis of asthma, wheeze and disease onset which may have resulted in some misclassification and potential information bias. Additionally, clinical assessment of asthma using spirometry was not available and we were not able to differentiate between asthma, COPD or even heart failure [49]. Therefore, there will be some attenuation of results due to misclassification. Moreover, there are likely to be some unmeasured confounders which may explain our findings. Lastly, the cross-sectional nature of the study means that we cannot mitigate the effects of reverse-causality. Hence, observed associations should be interpreted with caution.

Conclusions

Despite limitations, our findings highlight relationships between excess body weight as well as comorbidities such as reported heart disease and diagnosed asthma in Malawi among females. Future investigations using longitudinally collected data and clinical measures for asthma diagnosis are needed to better understand these observations and to inform healthcare delivery services for asthma in urban and rural Malawi.

Acknowledgements

We are very grateful to study participants and community leaders for taking part in our investigation.

We are also thankful to Malawi Epidemiology and Intervention Research Unit (previously known as Karonga Prevention Study) research clinicians, interviewers, research assistants and all other staff who were part of gathering data for the study.

References

1. Beasley R, Semprini A, Mitchell EA. Risk factors for asthma: is prevention possible? Lancet (London, England). 2015;386(9998):1075-85. Epub 2015/09/19. doi: 10.1016/s0140-6736(15)00156-7. PubMed PMID: 26382999.

2. WHO. Asthma Geneva: World Health Organisation; 2023 [updated 4 May 2023; cited 2023 21 June 2023]. Available from: https://www.who.int/news-room/fact-sheets/detail/asthma.

3. von Mutius E. Gene-environment interactions in asthma. The Journal of allergy and clinical immunology. 2009;123(1):3-11; quiz 2-3. Epub 2009/01/10. doi: 10.1016/j.jaci.2008.10.046. PubMed PMID: 19130922.

4. Johansson H, Mersha TB, Brandt EB, Khurana Hershey GK. Interactions between environmental pollutants and genetic susceptibility in asthma risk. Current opinion in immunology. 2019;60:156-62. Epub 2019/08/31. doi: 10.1016/j.coi.2019.07.010. PubMed PMID: 31470287.

5. Guilleminault L, Williams EJ, Scott HA, Berthon BS, Jensen M, Wood LG. Diet and Asthma: Is It Time to Adapt Our Message? Nutrients. 2017;9(11). Epub 2017/11/09. doi: 10.3390/nu9111227. PubMed PMID: 29117118; PubMed Central PMCID: PMCPMC5707699.

6. Peters U, Dixon AE, Forno E. Obesity and asthma. The Journal of allergy and clinical immunology. 2018;141(4):1169-79. Epub 2018/04/09. doi: 10.1016/j.jaci.2018.02.004. PubMed PMID: 29627041; PubMed Central PMCID: PMCPMC5973542.

7. Collaborators GRF. Global, regional, and national comparative risk assessment of 84 behavioural, environmental and occupational, and metabolic risks or clusters of risks, 1990-2016: a systematic analysis for the Global Burden of Disease Study 2016. Lancet (London, England). 2017;390(10100):1345-422. Epub 2017/09/19. doi: 10.1016/s0140-6736(17)32366-8. PubMed PMID: 28919119; PubMed Central PMCID: PMCPMC5614451.

8. Sheehan WJ, Phipatanakul W. Indoor allergen exposure and asthma outcomes. Current opinion in pediatrics. 2016;28(6):772-7. Epub 2016/11/03. doi: 10.1097/mop.0000000000000421. PubMed PMID: 27653703; PubMed Central PMCID: PMCPMC5127595.

9. Asher MI, Keil U, Anderson HR, Beasley R, Crane J, Martinez F, et al. International Study of Asthma and Allergies in Childhood (ISAAC): rationale and methods. The European respiratory journal. 1995;8(3):483-91. Epub 1995/03/01. PubMed PMID: 7789502.

10. Weiland SK, Bjorksten B, Brunekreef B, Cookson WO, von Mutius E, Strachan DP. Phase II of the International Study of Asthma and Allergies in Childhood (ISAAC II): rationale and methods. The European respiratory journal. 2004;24(3):406-12. Epub 2004/09/11. doi: 10.1183/09031936.04.00090303. PubMed PMID: 15358699.

11. Ellwood P, Asher MI, Beasley R, Clayton TO, Stewart AW. The international study of asthma and allergies in childhood (ISAAC): phase three rationale and methods. The international journal of tuberculosis and lung disease : the official journal of the International Union against Tuberculosis and Lung Disease. 2005;9(1):10-6. Epub 2005/01/29. PubMed PMID: 15675544.

12. de Nijs SB, Venekamp LN, Bel EH. Adult-onset asthma: is it really different? European Respiratory Review. 2013;22(127):44-52. doi: 10.1183/09059180.00007112.

13. Chowdhury NU, Guntur VP, Newcomb DC, Wechsler ME. Sex and gender in asthma. Eur Respir Rev. 2021;30(162). Epub 20211117. doi: 10.1183/16000617.0067-2021. PubMed PMID: 34789462; PubMed Central PMCID: PMCPMC8783601.

14. Rasmussen F, Hancox RJ. Mechanisms of obesity in asthma. Current opinion in allergy and clinical immunology. 2014;14(1):35-43. Epub 2013/12/05. doi: 10.1097/aci.0000000000000024. PubMed PMID: 24300417.

15. Wang L, Wang K, Gao X, Paul TK, Cai J, Wang Y. Sex difference in the association between obesity and asthma in U.S. adults: Findings from a national study. Respiratory Medicine. 2015;109(8):955-62. doi: https://doi.org/10.1016/j.rmed.2015.06.001.

16. Brumpton B, Langhammer A, Romundstad P, Chen Y, Mai X-M. General and abdominal obesity and incident asthma in adults: the HUNT study. European Respiratory Journal. 2013;41(2):323-9. doi: 10.1183/09031936.00012112.

17. Morgan BW, Siddharthan T, Grigsby MR, Pollard SL, Kalyesubula R, Wise RA, et al. Asthma and Allergic Disorders in Uganda: A Population-Based Study Across Urban and Rural Settings. J Allergy Clin Immunol Pract. 2018;6(5):1580-7.e2. Epub 20180201. doi: 10.1016/j.jaip.2017.11.032. PubMed PMID: 29361510; PubMed Central PMCID: PMCPMC6050146.

18. Siddharthan T, Grigsby M, Morgan B, Kalyesubula R, Wise RA, Kirenga B, et al. Prevalence of chronic respiratory disease in urban and rural Uganda. Bull World Health Organ. 2019;97(5):318-27. Epub 20190326. doi: 10.2471/blt.18.216523. PubMed PMID: 31551628; PubMed Central PMCID: PMCPMC6747035.

19. Kirenga BJ, de Jong C, Katagira W, Kasozi S, Mugenyi L, Boezen M, et al. Prevalence and factors associated with asthma among adolescents and adults in Uganda: a general population based survey. BMC Public Health. 2019;19(1):227. doi: 10.1186/s12889-019-6562-2.

20. Miranda JJ, Bernabe-Ortiz A, Gilman RH, Smeeth L, Malaga G, Wise RA, et al. Multimorbidity at sea level and high-altitude urban and rural settings: The CRONICAS Cohort Study. J Comorb. 2019;9:2235042x19875297. Epub 20191106. doi: 10.1177/2235042x19875297. PubMed PMID: 34249770; PubMed Central PMCID: PMCPMC8240099.

21. Desalu OO, Adeoti AO, Ojuawo OB, Aladesanmi AO, Oguntoye MS, Afolayan OJ, et al. Urban-Rural Differences in the Epidemiology of Asthma and Allergies in Nigeria: A Population-Based Study. J Asthma Allergy. 2021;14:1389-97. Epub 20211110. doi: 10.2147/jaa.S333133. PubMed PMID: 34866916; PubMed Central PMCID: PMCPMC8637762.

22. Buist AS, Vollmer WM, Sullivan SD, Weiss KB, Lee TA, Menezes AM, et al. The Burden of Obstructive Lung Disease Initiative (BOLD): rationale and design. Copd. 2005;2(2):277-83. Epub 2006/12/02. PubMed PMID: 17136954.

23. Buist AS, McBurnie MA, Vollmer WM, Gillespie S, Burney P, Mannino DM, et al. International variation in the prevalence of COPD (the BOLD Study): a population-based prevalence study. Lancet (London, England). 2007;370(9589):741-50. Epub 2007/09/04. doi: 10.1016/s0140-6736(07)61377-4. PubMed PMID: 17765523.

24. Mortimer K, Reddel HK, Pitrez PM, Bateman ED. Asthma management in low- and middle-income countries: case for change. European Respiratory Journal. 2022:2103179. doi: 10.1183/13993003.03179-2021.

25. Coleman M. Teaching corner: Adult asthma in Malawi. Malawi medical journal : the journal of Medical Association of Malawi. 2011;23(1):22-4. Epub 2011/03/01. PubMed PMID: 23638253; PubMed Central PMCID: PMCPmc3615331.

26. Cook J, Jefferis O, Matchere P, Mbale E, Rylance J. Sickle-cell disease in Malawian children is associated with restrictive spirometry: a cross-sectional survey. The international journal of tuberculosis and lung disease : the official journal of the International Union against Tuberculosis and Lung Disease. 2013;17(9):1235-8. Epub 2013/07/06. doi: 10.5588/ijtld.12.0965. PubMed PMID: 23827770; PubMed Central PMCID: PMCPmc3826120.

27. Wroe EB, Kalanga N, Mailosi B, Mwalwanda S, Kachimanga C, Nyangulu K, et al. Leveraging HIV platforms to work toward comprehensive primary care in rural Malawi: the Integrated Chronic Care Clinic. Healthcare (Amsterdam, Netherlands). 2015;3(4):270-6. Epub 2015/12/25. doi: 10.1016/j.hjdsi.2015.08.002. PubMed PMID: 26699356.

28. Manjomo RC, Mwagomba B, Ade S, Ali E, Ben-Smith A, Khomani P, et al. Managing and monitoring chronic non-communicable diseases in a primary health care clinic, Lilongwe, Malawi. Public health action. 2016;6(2):60-5. Epub 2016/07/01. doi: 10.5588/pha.16.0003. PubMed PMID: 27358797; PubMed Central PMCID: PMCPmc4913686.

29. Meghji J, Nadeau G, Davis KJ, Wang D, Nyirenda MJ, Gordon SB, et al. Noncommunicable Lung Disease in Sub-Saharan Africa. A Community-based Cross-Sectional Study of Adults in Urban Malawi. American journal of respiratory and critical care medicine. 2016;194(1):67-76. Epub 2016/01/21. doi: 10.1164/rccm.201509-1807OC. PubMed PMID: 26788760; PubMed Central PMCID: PMCPMC4960629.

30. Nightingale R, Lesosky M, Flitz G, Rylance SJ, Meghji J, Burney P, et al. Noncommunicable Respiratory Disease and Air Pollution Exposure in Malawi (CAPS). A Cross-Sectional Study. American journal of respiratory and critical care medicine. 2019;199(5):613-21. Epub 2018/08/25. doi: 10.1164/rccm.201805-0936OC. PubMed PMID: 30141966; PubMed Central PMCID: PMCPMC6396863.

31. Rylance S, Jewell C, Naunje A, Mbalume F, Chetwood JD, Nightingale R, et al. Non-communicable respiratory disease and air pollution exposure in Malawi: a prospective cohort study. Thorax. 2020;75(3):220-6. Epub 2020/02/23. doi: 10.1136/thoraxjnl-2019-213941. PubMed PMID: 32079666; PubMed Central PMCID: PMCPMC7063402.

32. Njoroge MW, Rylance S, Nightingale R, Gordon S, Mortimer K, Burney P, et al. Cohort profile: The Chikwawa lung health cohort; a population-based observational non-communicable respiratory disease study of adults in Malawi. PLoS One. 2020;15(11):e0242226. Epub 2020/11/13. doi: 10.1371/journal.pone.0242226. PubMed PMID: 33180873; PubMed Central PMCID: PMCPMC7660567.

33. Crampin AC, Kayuni N, Amberbir A, Musicha C, Koole O, Tafatatha T, et al. Hypertension and diabetes in Africa: design and implementation of a large population-based study of burden and risk factors in rural and urban Malawi. Emerging themes in epidemiology. 2016;13:3. Epub 2016/02/04. doi: 10.1186/s12982-015-0039-2. PubMed PMID: 26839575; PubMed Central PMCID: PMCPmc4736489.

34. Price AJ, Crampin AC, Amberbir A, Kayuni-Chihana N, Musicha C, Tafatatha T, et al. Prevalence of obesity, hypertension, and diabetes, and cascade of care in sub-Saharan Africa: a cross-sectional, population-based study in rural and urban Malawi. The lancet Diabetes & endocrinology. 2018;6(3):208-22. Epub 2018/01/27. doi: 10.1016/s2213-8587(17)30432-1. PubMed PMID: 29371076; PubMed Central PMCID: PMCPMC5835666.

35. Crampin AC, Dube A, Mboma S, Price A, Chihana M, Jahn A, et al. Profile: the Karonga Health and Demographic Surveillance System. International journal of epidemiology. 2012;41(3):676-85. Epub 2012/06/26. doi: 10.1093/ije/dys088. PubMed PMID: 22729235; PubMed Central PMCID: PMCPmc3396313.

36. National Statistical Office. 2018 Malawi Population and Housing: Main Report. Lilongwe: National Statistical Office, 2019.

37. Burney PG, Laitinen LA, Perdrizet S, Huckauf H, Tattersfield AE, Chinn S, et al. Validity and repeatability of the IUATLD (1984) Bronchial Symptoms Questionnaire: an international comparison. The European respiratory journal. 1989;2(10):940-5. Epub 1989/11/01. PubMed PMID: 2606194.

38. Amberbir A, Medhin G, Hanlon C, Britton J, Venn A, Davey G. Frequent use of paracetamol and risk of allergic disease among women in an Ethiopian population. PLoS One. 2011;6(7):e22551. Epub 2011/08/04. doi: 10.1371/journal.pone.0022551. PubMed PMID: 21811632; PubMed Central PMCID: PMCPMC3141069.

39. WHO. Physical activity Geneva, Switzerland World Health Organisation 2022 [updated 5 October 2022; cited 2022 12 November 2022]. Available from: https://www.who.int/news-room/fact-sheets/detail/physical-activity.

40. WHO. Global Database on Body Mass Index: BMI classification Geneva: World Health Organisation; 2006 [cited 2016]. Available from: http://apps.who.int/bmi/index.jsp?introPage=intro_3.html.

41. McLean E, Dube A, Saul J, Branson K, Luhanga M, Mwiba O, et al. Implementing electronic data capture at a well-established health and demographic surveillance site in rural northern Malawi. Glob Health Action. 2017;10(1):1367162. doi: 10.1080/16549716.2017.1367162. PubMed PMID: 28922071; PubMed Central PMCID: PMCPMC5645702.

42. Katz MH. Multivariable Analysis: A Practical Guide for Clinicians Cambridge: Cambridge University Press; 2007.

43. Addo-Yobo EO, Woodcock A, Allotey A, Baffoe-Bonnie B, Strachan D, Custovic A. Exercise-induced bronchospasm and atopy in Ghana: two surveys ten years apart. PLoS medicine. 2007;4(2):e70. Epub 2007/03/01. doi: 10.1371/journal.pmed.0040070. PubMed PMID: 17326711; PubMed Central PMCID: PMCPMC1808098.

44. Poyser MA, Nelson H, Ehrlich RI, Bateman ED, Parnell S, Puterman A, et al. Socioeconomic deprivation and asthma prevalence and severity in young adolescents. European Respiratory Journal. 2002;19(5):892-8. doi: 10.1183/09031936.02.00238402.

45. Iribarren C, Tolstykh IV, Eisner MD. Are patients with asthma at increased risk of coronary heart disease? International journal of epidemiology. 2004;33(4):743-8. doi: 10.1093/ije/dyh081.

46. Iribarren C, Tolstykh IV, Miller MK, Sobel E, Eisner MD. Adult Asthma and Risk of Coronary Heart Disease, Cerebrovascular Disease, and Heart Failure: A Prospective Study of 2 Matched Cohorts. American Journal of Epidemiology. 2012;176(11):1014-24. doi: 10.1093/aje/kws181.

47. Wang L, Gao S, Yu M, Sheng Z, Tan W. Association of asthma with coronary heart disease: A meta analysis of 11 trials. PLoS One. 2017;12(6):e0179335. Epub 20170613. doi: 10.1371/journal.pone.0179335. PubMed PMID: 28609456; PubMed Central PMCID: PMCPMC5469478.

48. Gurgone D, McShane L, McSharry C, Guzik TJ, Maffia P. Cytokines at the Interplay Between Asthma and Atherosclerosis? Front Pharmacol. 2020;11:166. Epub 20200304. doi: 10.3389/fphar.2020.00166. PubMed PMID: 32194407; PubMed Central PMCID: PMCPMC7064545.

49. Wong CW, Tafuro J, Azam Z, Satchithananda D, Duckett S, Barker D, et al. Misdiagnosis of Heart Failure: A Systematic Review of the Literature. Journal of Cardiac Failure. 2021;27(9):925-33. doi: https://doi.org/10.1016/j.cardfail.2021.05.014.

50. Zolotareva O, Saik OV, Königs C, Bragina EY, Goncharova IA, Freidin MB, et al. Comorbidity of asthma and hypertension may be mediated by shared genetic dysregulation and drug side effects. Sci Rep. 2019;9(1):16302. Epub 20191108. doi: 10.1038/s41598-019-52762-w. PubMed PMID: 31705029; PubMed Central PMCID: PMCPMC6841742.

51. Koebnick C, Fischer H, Daley MF, Ferrara A, Horberg MA, Waitzfelder B, et al. Interacting effects of obesity, race, ethnicity and sex on the incidence and control of adult-onset asthma. Allergy, asthma, and clinical immunology : official journal of the Canadian Society of Allergy and Clinical Immunology. 2016;12:50. Epub 2016/10/26. doi: 10.1186/s13223-016-0155-8. PubMed PMID: 27777591; PubMed Central PMCID: PMCPMC5069790.

52. Sood A, Qualls C, Schuyler M, Arynchyn A, Alvarado JH, Smith LJ, et al. Adult-onset asthma becomes the dominant phenotype among women by age 40 years. the longitudinal CARDIA study. Annals of the American Thoracic Society. 2013;10(3):188-97. Epub 2013/06/28. doi: 10.1513/AnnalsATS.201212-115OC. PubMed PMID: 23802814; PubMed Central PMCID: PMCPMC3960903.

53. Umetsu DT. Mechanisms by which obesity impacts asthma. Thorax. 2016. Epub 2016/09/28. doi: 10.1136/thoraxjnl-2016-209130. PubMed PMID: 27672120.

54. Leynaert B, Sunyer J, Garcia-Esteban R, Svanes C, Jarvis D, Cerveri I, et al. Gender differences in prevalence, diagnosis and incidence of allergic and non-allergic asthma: a population-based cohort. Thorax. 2012;67(7):625-31. doi: 10.1136/thoraxjnl-2011-201249.

55. Hansen S, Probst-Hensch N, Keidel D, Dratva J, Bettschart R, Pons M, et al. Gender differences in adult-onset asthma: results from the Swiss SAPALDIA cohort study. European Respiratory Journal. 2015;46(4):1011-20. doi: 10.1183/13993003.02278-2014.

56. Currow DC, Dal Grande E, Sidhu C, Ekström M, Johnson MJ. The independent association of overweight and obesity with breathlessness in adults: a cross-sectional, population-based study. The European respiratory journal. 2017;50(3). Epub 20170927. doi: 10.1183/13993003.00558-2017. PubMed PMID: 28954771.

57. Banda HT, Thomson R, Mortimer K, Bello GAF, Mbera GB, Malmborg R, et al. Community prevalence of chronic respiratory symptoms in rural Malawi: Implications for policy. PLoS One. 2017;12(12):e0188437. Epub 2017/12/08. doi: 10.1371/journal.pone.0188437. PubMed PMID: 29216193; PubMed Central PMCID: PMCPMC5720679.

58. Verlato G, Nguyen G, Marchetti P, Accordini S, Marcon A, Marconcini R, et al. Smoking and New-Onset Asthma in a Prospective Study on Italian Adults. Int Arch Allergy Immunol. 2016;170(3):149-57. Epub 20160818. doi: 10.1159/000446509. PubMed PMID: 27532893.

59. Messeri P, Cantrell J, Mowery P, Bennett M, Hair E, Vallone D. Examining differences in cigarette smoking prevalence among young adults across national surveillance surveys. PLOS ONE. 2019;14(12):e0225312. doi: 10.1371/journal.pone.0225312.

60. Nightingale R, Lesosky M, Flitz G, Rylance SJ, Meghji J, Burney P, et al. Non-Communicable Respiratory Disease and Air Pollution Exposure in Malawi (CAPS): A Cross-Sectional Study. American journal of respiratory and critical care medicine. 2018. Epub 2018/08/25. doi: 10.1164/rccm.201805-0936OC. PubMed PMID: 30141966.

61. Gordon SB, Bruce NG, Grigg J, Hibberd PL, Kurmi OP, Lam KB, et al. Respiratory risks from household air pollution in low and middle income countries. Lancet Respir Med. 2014;2(10):823-60. Epub 20140902. doi: 10.1016/s2213-2600(14)70168-7. PubMed PMID: 25193349; PubMed Central PMCID: PMCPMC5068561.

62. Po JYT, FitzGerald JM, Carlsten C. Respiratory disease associated with solid biomass fuel exposure in rural women and children: systematic review and meta-analysis. Thorax. 2011;66(3):232-9. doi: 10.1136/thx.2010.147884.

63. Holtjer JCS, Bloemsma LD, Beijers R, Cornelissen MEB, Hilvering B, Houweling L, et al. Identifying risk factors for COPD and adult-onset asthma: an umbrella review. Eur Respir Rev. 2023;32(168). Epub 20230503. doi: 10.1183/16000617.0009-2023. PubMed PMID: 37137510; PubMed Central PMCID: PMCPMC10155046.

64. Rylance S, Masekela R, Banda NPK, Mortimer K. Determinants of lung health across the life course in sub-Saharan Africa. The international journal of tuberculosis and lung disease : the official journal of the International Union against Tuberculosis and Lung Disease. 2020;24(9):892-901. doi: 10.5588/ijtld.20.0083. PubMed PMID: 33156755.

Supporting Information

- **S1 Fig. Overlap between 'Diagnosed asthma with medication use' and related symptoms**
  Figure is a proportional Venn diagram showing the overlap between the outcomes 'Diagnosed asthma with medication use', 'Wheeze in the past 12 months' and 'Wheeze in the past 12 months plus shortness of breath'. The area representing each variable is proportional to the number of people reporting this outcome.

- **S1 Table - Univariable Analysis - Factors associated with diagnosed asthma stratified by sex & urban-rural area**

- **S2 Table - Univariable Analysis - Factors associated with wheeze in the past 12 months stratified by sex & urban-rural area**

Figure 1

Click here to access/download;Figure;Figure 1 .tiff

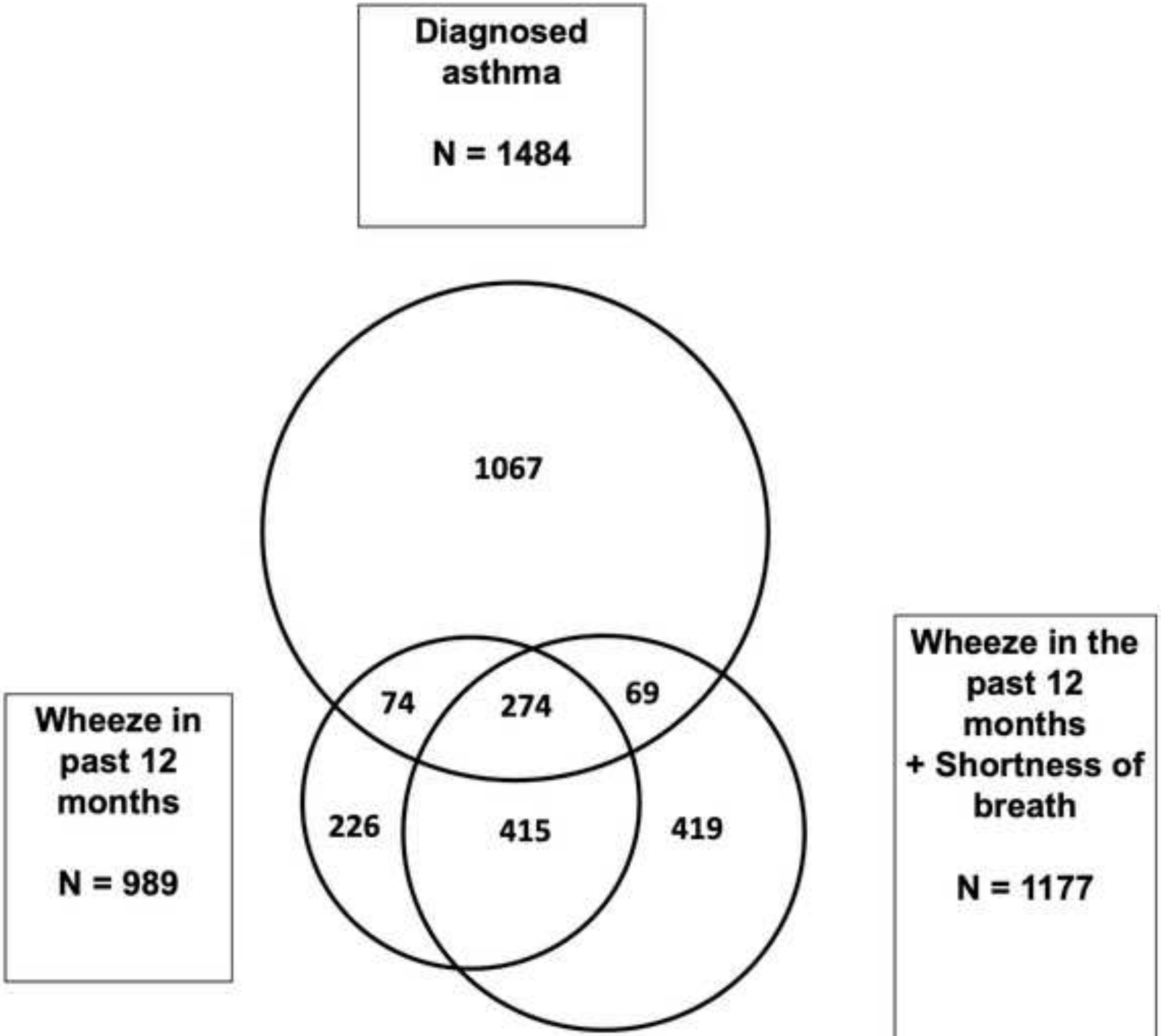

Figure S1

Click here to access/download
Supporting Information
Figure S1 .tiff

S1 Table

Click here to access/download
Supporting Information
S1 Table.docx

S2 Table

Click here to access/download
Supporting Information
S2 Table.docx

Factors associated with self-reported diagnosed asthma in urban and rural Malawi: observations from a population-based study of non-communicable diseases

Abena S. Amoah[1,2,#a*], Estelle McLean[1,2] Alison J. Price[1,2], Alemayehu Amberbir[3], Amelia C. Crampin[1,2,4]

1. Department of Population Health, Faculty of Epidemiology and Population Health, London School of Hygiene and Tropical Medicine, London, United Kingdom
2. Malawi Epidemiology and Intervention Research Unit, Malawi
3. University of Global Health Equity, Kigali, Rwanda
4. School of Health and Wellbeing, University of Glasgow, Glasgow, United Kingdom

#a. Current Address: Leiden University Center for Infectious

Diseases, Leiden University Medical Center, Leiden, The Netherlands

*Corresponding Author: Abena S. Amoah

Email: a.s.amoah@lumc.nl

Key Words: Asthma, Epidemiology, Noncommunicable diseases, Obesity, Urbanization, Wheeze

Abstract

Background: The growing burden of asthma in low- and middle-income countries has been linked to urbanisation and lifestyle changes. However, this burden has not been well ~~been less~~ characterised in adults~~compared to children~~. Therefore, we investigated the prevalence of self-reported diagnosed asthma and associated ~~risk~~ factors in urban and rural adults in Malawi, Southern Africa.

Methods: Within a cross-sectional population-based survey to determine the burden and risk factors for non-communicable diseases (NCDs) in the city of Lilongwe and ~~in~~ rural Karonga district, we collected information on self-reported previously diagnosed ~~prior~~ asthma ~~diagnosis~~ and asthma-related symptoms using an interviewer-led questionnaire. Other data collected included: demographic characteristics, socioeconomic status indicators, NCD comorbidities, environmental exposures, and anthropometric measurements. We used multivariable logistic regression models to explore ~~investigate~~ factors associated with self-reported asthma adjusting for variables associated with the outcome in univariable analysis. Findings were corrected for multiple comparisons using the Bonferroni method.

Results: We analysed data from 30,483 adult participants (54.6% urban,~~–~~45.4% rural and 61.9% female). A prior asthma diagnosis was reported in 5.1% ~~(95%CI 4.9%-5.6%)~~ of urban and 4.5% ~~(95% CI 4.1%-4.8%)~~ of rural participants. In urban females, being obese (>30 kg/m^2) compared to normal weight (18.5–24.9 kg/m^2) was associated with greater odds of asthma (OR=1.59, 95% CI [1.26– 2.01], p<0.001), after adjusting for confounders. We observed associations between previously~~ior~~ diagnosed heart disease and asthma in female participants which remained significant in rural females after Bonferroni correction ~~urban- (OR=1.76, 95%CI [1.16– 2.68], p=0.008) and rural- females~~ (OR=2.30,95%CI [1.32– 4.02], p=0.003). Among rural males, current smokers had reduced odds of diagnosed asthma (OR=0.46,95%CI [0.27–0.79], p=0.004) compared to those who had never smoked.

Conclusions: In Malawi the prevalence of self-reported diagnosed asthma was greatest in females and urban dwellers. Notably, our findings indicate relationships between excess body weight as well as comorbidities and diagnosed asthma in females. Future investigations using longitudinally collected data and clinical measurements of asthma are needed to better understand ~~the nature of~~ these associations.

Introduction

Asthma is ~~the~~ one of the most common chronic conditions [1] affecting an estimated 262 million people worldwide and in 2019, resulted in 455,000 premature deaths ~~in 2019~~ [2]. Most asthma-related deaths occur in low- and middle-income countries (LMICs) where health-care resources are constrained, and the condition~~nd asthma~~ remains under-diagnosed and under-treated [2].

Asthma is made up of multiple phenotypes and represents complex interactions between genetic and environmental determinants [3]. Factors associated with urbanisation such as greater exposure to environmental pollutants [4], changes in diet ~~(for example - increased intake of saturated fatty acids, cholesterol, refined grains, highly processed foods and red meats)~~ [5], a greater body mass index [6, 7] and indoor allergen exposure [8], have all been linked to the rising prevalence in asthma worldwide ~~asthma prevalence~~. In children, the global burden of asthma and associated risk factors have been relatively well-characterised [9-11]. ~~However, much~~Much less is known about asthma in adults, particularly in LMICs. From observations made primarily in high income countries (HICs), adult-onset asthma is largely non-allergic [12], more common in females [13], ~~and is~~ associated with an accelerated decline in lung function [12] and linked to~~as well as~~ obesity [14-16]. The few studies conducted on asthma in LMICs do indicate marked urban-rural variations in asthma burden [17-21]. Initiatives such as the Burden of Obstructive Lung Disease (BOLD) study which collected country-specific data to characterise lung disease in adults worldwide [22, 23] have been addressing the evidence gap between HICs and LMICs. However, ~~-~~ diagnostic challenges exist and in many LMICs where the availability of diagnostic tools for asthma are limited, diagnosis and management of the condition rely largely on clinical observations by health-care professionals and reported symptoms thus resulting in~~leading to~~ under-diagnosis and under-treatment [24].

In Malawi, a low-income country in sub-Saharan Africa, asthma is commonly diagnosed in primary health-care settings [25]. Most published studies from Malawi have been hospital-based and focused on the diagnosis and management of multiple respiratory conditions including asthma [26-28].

Recently, ~~a number of~~several population-based studies conducted in the Southern region of Malawi ~~have~~ investigated lung function, respiratory ~~symptoms~~symptoms, and the burden of noncommunicable lung disease. [29-32]~~.~~ Although not focused on asthma, some of these studies ~~have~~ established associations between biomass smoke exposure, smoking, sex and respiratory symptoms in adults in Southern Malawi ~~.~~[29, 30].

~~Between May 2013 and April 2017, the Malawi Epidemiology and Intervention Research Unit (previously known as Karonga Prevention Study) conducted a large population-based investigation of the burden and risk factors for non-communicable diseases (NCDs) in urban and rural adults in Malawi, for which the objectives, design, populations and methodology are described elsewhere [33, 34]. Published findings from this investigation observed notable differences in the prevalence of NCD outcomes such as hypertension in urban versus rural area as well as in males versus females [34, 35]. Within this large study, information was also collected on self-reported diagnosed asthma, asthma symptoms and risk factors for asthma.~~

The aim of our current study was to investigate the prevalence of self-reported diagnosed asthma in adults. Additionally, we sought to explore ~~(aged ≥18 years) as well as~~ factors associated with asthma ~~asthma~~, including demographic characteristics, anthropometric measurements, socioeconomic status indicators, NCD comorbidities and environmental smoke exposures.

Field Code Changed

Field Code Changed

Methods

Study Design

Between May 2013 and April 2017, the Malawi Epidemiology and Intervention Research Unit (previously known as Karonga Prevention Study) conducted a large population-based cross-sectional investigation of the burden and risk factors for non-communicable diseases (NCDs) in urban and rural adults in Malawi.~~,~~ The~~for which the objectives,~~ design, population descriptions~~s~~ and detailed methodology for this study have been ~~are~~ described elsewhere [33, 34]. Published findings from this investigation observed notable differences in the prevalence of NCD outcomes such as hypertension in urban versus rural area as well as in males versus females [34]. ~~[34, 35].~~ Within this large study, information was also collected on self-reported diagnosed asthma, asthma symptoms and factors potentially associated with ~~risk factors for~~ asthma.

Field Code Changed

Field Code Changed

Formatted: Normal, Justified, Space After: 8 pt, Line spacing: Double

Study area and population

For our investigation, the~~The~~ rural study area was within the established Karonga Health and Demographic Surveillance Site (Karonga HDSS) [35] where approximately 50,000 individuals reside ~~ing~~ in a geographical area of 135km^2 in the southern part of Karonga District in the Northern Region of Malawi and are under continuous demographic surveillance. The Karonga HDSS population is predominantly rural with an economy based on subsistence farming, fishing and petty trading [35]. Our urban study area was in Lilongwe, the capital city of Malawi, which is in the Central Region of the country. The study area in Lilongwe wa~~i~~s an enumerated zone ~~of Area 25,~~ in a~~a~~ high-density residential area named Area 25 which has ~~with~~ an estimated population of 107,316 individuals [36] of mixed socioeconomic status [33]. Enumeration ~~-~~involved collecting data on household membership, location details, and information on individuals ≥18 years [33]. Data collection for our study was between 16 May 2013 and 25 April 2017 in both study areas.

Data collection

Study questionnaire

Questions from the International Union against Tuberculosis and Lung Disease (IUALTD) bronchial symptoms questionnaire [37] were administered to ~~study~~ participants along with questions on demographic parameters, socioeconomic indicators, medical history as well as lifestyle factors. These measurements have been used elsewhere in Africa [38].

The specific questions related to asthma and its symptoms were:

1. *Have you ever been diagnosed with asthma, asthmatic bronchitis or allergic bronchitis by a doctor or other health professional? If yes,~~-~~ what age were you when ~~you~~ first diagnosed?*
2. *Are you on regular medication for asthma? If yes, name of medication* ____________
3. *Have you had wheezing or whistling in your chest at any time in the past year?* `
4. *In the last year, have you ever had an attack of wheezing or whistling that has made you feel short of breath?*

The study questionnaire was developed in English, translated into Chitumbuka (for the rural area) and Chichewa (for the urban area). It was then ~~,~~independently back translated and tested to ensure understanding and acceptability before implementation. Questionnaires were administered by trained interviewers using electronic tablet computers.

Questions on potential risk factors for asthma such as age, area of residence (urban or rural), indicators of socio-economic position including education level, occupation, household income and ownership of certain possessions, physical activity levels, NCD comorbidities and environmental exposures such as cigarette smoke, firewood smoke exposure and water source were included.

For these analyses, we categorised education based on Malawi educational levels ranging from no formal education, primary school (Standard 1-5 and Standard 6-8~~), secondary~~), secondary school and tertiary education. Physical activity was based on minutes spent on physical activity according to the

World Health Organisation (WHO)'s guidelines [39] and three categories were generated (low, moderate and high physical activity). We generated a wealth score based on possessions (electricity, lamp, mobile phone etc.) and an income score based on ~~reported income~~reported income. The NCD comorbidities of interest were ~~all~~ previously diagnosed diabetes mellitus, heart disease, high blood pressure and stroke as reported by study participants. For smoke exposures, 'smoking status' was based on individual smoking history while 'smoker in household' gauged second-hand smoke exposure at home. We combined information on firewood use for cooking and whether cooking areas were ventilated or non-ventilated to generate a firewood exposure variable.

Anthropometric measurements

For each study ~~participant, we~~participant, we determined weight and height measurements using a calibrated scale and portable stadiometer respectively. Body Mass Index (BMI) was calculated by dividing weight (kg) by height (m) squared and was categorised according to the WHO's BMI classification for adults: underweight (less than 18.5kg/m^2), normal weight (18.5-24.9kg/m^2), overweight (25.0-29.9kg/m^2) and obese (>30.0kg/m^2) [40].

Data management and quality assurance

Data was predominantly captured electronically via protected tablet computers using forms pre-programmed in Open Data Kit (https://getodk.org/) which were uploaded each day to a database housed in secure central server at each research site. Data quality was assured through rigorous training of Interviewers and the use of an interviewer's guide to ensure uniformity of data collection. Pre-programmed data checks were performed in the MEIRU data offices to identify any discrepancies which were addressed immediately. Uploaded data were also regularly extracted and checked for scientific validity. Data management processes were strictly controlled through adherence to set protocols and a central data dictionary [41].

Formatted: Justified, Line spacing: Double

Formatted: Normal

Informed consent and ethical approval

We provided a full explanation of the study objectives and procedures to potential participants prior to seeking individual written consent. Ethical approval for the study was granted by the Malawi National Health Sciences Research Committee (protocol number #1072) and the London School of Hygiene & Tropical Medicine Ethics Committee (protocol number #6303).

Statistical analysis

Our primary outcome was self-reported diagnosed asthma. A secondary outcome was reported wheeze in the past 12 months. We also considered other asthma-related outcomes for descriptive analysis. We investigated differences in baseline characteristics stratified by sex and urban-rural area using Pearson's $\chi 2$ tests (for categorical variables) and Mann-Whitney U tests (for continuous variables). In preliminary analysis, we evaluated the extent to which associations between our covariates and outcome of asthma varied by sex and urban-rural area. To this end, we generated models fitted with an interaction term between sex and each covariate as well as separate models with interaction terms for urban-rural area. Our exploration showed evidence that the association between some sociodemographic indicators (for example, age-group, BMI, education, and work status) varied by sex and urban-rural area ($p<0.05$). Therefore, our logistic regression models were generated stratified by sex and urban-rural area. In univariable logistic regression analysis, associations between risk factors of interest and study outcomes (asthma and wheeze in the past 12 months) were examined using logistic regression models. A two-sided p-value threshold of <0.100 was used to determine factors for inclusion in multivariable analysis. Final mModels were adjusted for all factors found to be significant in univariable analysis. To account for the effect of multiple testing in our investigation,, and the increased probability of detecting a significant finding by chance alone, we applied a Bonferroni correction where alpha of 5% was divided by the number variables in the final logistic regression model [42]. [34, 41]Therefore, a corrected alpha of 0.4% (p=0.004) was considered as the threshold for statistically significance considered a two-sided p-value of 1% (p=0.01) as

Formatted: Not Highlight

Formatted: Not Highlight

Formatted: Not Highlight

Formatted: Not Highlight

Formatted: Not Highlight

~~statistically significant~~ for our ~~all~~ multivariable regression analyses. All analyses were conducted using STATA version 17 software (StataCorp, College Station, Texas).

Results

Characteristics of the study population

A total of 30,483 participants (54.6% urban, 45.4% rural and 61.9% female) were included in the analyses. Table 1 shows characteristics of study participants stratified by sex and urban-rural area. According to WHO BMI classifications, 18.0 % of urban females were obese (30.0 kg/m^2) compared to 7.7% % of rural females. By contrast, 3.9% of urban males were classified as obese and only 1.0% of rural males. ~~The majority of~~Most individuals in all groups reported a physical activity rating categorized as 'high' according to the WHO physical activity standards.

Table 1. Characteristics of study population stratified by sex and urban-rural area

FACTOR	~~Females (~~Females (N=18,855)				Males (N=11,628)			
	LILONGWE – URBAN	KARONGA - RURAL	TOTAL	p-value	LILONGWE – URBAN	KARONGA - RURAL	TOTAL	p-value
**Age ~~group (~~group (years)**								
18-29	5,581 (51.4)	2,952 (36.9)	8,533 (45.3)	**<0.001**	2,944 (50.8)	2,224 (38.2)	5,168 (44.4)	**<0.001**
30-39	3,027 (27.9)	1,980 (24.8)	5,007 (26.6)		1,417 (24.4)	1,425 (24.4)	2,842 (24.4)	
40-49	1,140 (10.5)	1,232 (15.4)	2,372 (12.6)		695 (12.0)	905 (15.5)	1,600 (13.8)	
50-59	623 (5.7)	835 (10.4)	1,458 (7.7)		368 (6.4)	576 (9.9)	944 (8.1)	
60-69	317 (2.9)	522 (6.5)	839 (4.5)		242 (4.2)	316 (5.4)	558 (4.8)	
70+	171 (1.6)	474 (5.9)	645 (3.4)		132 (2.3)	384 (6.6)	516 (4.4)	
**Body Mass Index (kg/m2)**								
Underweight (<18.5)	418 (4.1)	529 (7.1)	947 (5.3)	**<0.001**	410 (7.1)	564 (9.7)	974 (8.4)	<0.001
Normal (18.5-24.9)	5298 (51.5)	4900 (65.6)	10198 (57.5)		4307 (74.3)	4729 (81.3)	9036 (77.8)	
Overweight (25.0-29.9)	2723 (26.5)	1465 (19.6)	4188 (23.6)		855 (14.8)	463 (8.0)	1318 (11.4)	
Obese(>30.0)	1846 (18.0)	572 (7.7)	2418 (13.6)		224 (3.9)	59 (1.0)	283 (2.4)	
**Physical activity rating**								
Low	178 (1.6)	148 (1.9)	326 (1.7)	**0.001**	240 (4.1)	145 (2.5)	385 (3.3)	<0.001
Moderate	284 (2.6)	278 (3.5)	562 (3.0)		775 (13.4)	624 (10.7)	1,399 (12.0)	
High	10,398 (95.8)	7,569 (94.7)	17,967 (95.3)		4,783 (82.5)	5,061 (86.8)	9,844 (84.7)	
**Household income, median (IQR)**	7 (5 - 9)	4 (2 - 5)	5 (3 - 8)	**<0.001**	7 (5 - 9)	4 (3 - 6)	6 (4 - 8)	
**Household asset index, median (IQR)**	7 (5 - 9)	4 (2 - 6)	6 (3 - 8)	**<0.001**	7 (5 - 9)	4 (3 - 7)	6 (3 - 8)	
**Education**								
No formal	505 (4.7)	496 (6.2)	1,001 (5.3)	**<0.001**	78 (1.4)	99 (1.7)	177 (1.5)	<0.001
Primary: Standard 1-5	1,059 (9.8)	1,366 (17.1)	2,425 (12.9)		272 (4.7)	634 (10.9)	906 (7.8)	
Primary: Standard 6-8	2,486 (22.9)	4,065 (50.8)	6,551 (34.7)		814 (14.0)	2,407 (41.3)	3,221 (27.7)	
Secondary	5,357 (49.3)	1,990 (24.9)	7,347 (39.0)		3,333 (57.5)	2,515 (43.1)	5,848 (50.3)	
Tertiary	1,453 (13.4)	78 (1.0)	1,531 (8.1)		1,301 (22.4)	175 (3.0)	1,476 (12.7)	
**Work Status**								
Not working	2,525 (23.3)	730 (9.1)	3,255 (17.3)	**<0.001**	2,085 (36.0)	1,012 (17.4)	3,097 (26.6)	<0.001
Housework	4,619 (42.5)	763 (9.5)	5,382 (28.5)		364 (6.3)	70 (1.2)	434 (3.7)	
Farming/fishing	57 (0.5)	5,266 (65.9)	5,323 (28.2)		30 (0.5)	3,369 (57.8)	3,399 (29.2)	
Self-employed	1,957 (18.0)	1,040 (13.0)	2,997 (15.9)		1,163 (20.1)	852 (14.6)	2,015 (17.3)	
Employed	1,702 (15.7)	196 (2.5)	1,898 (10.1)		2,156 (37.2)	527 (9.0)	2,683 (23.1)	
**Diagnosed Diabetes Mellitus**								
No	10704 (98.6)	7926 (99.1)	18630 (98.8)	**<0.001**	5706 (98.4)	5789 (99.3)	11495 (98.9)	<0.001
Yes	156 (1.4)	69 (0.9)	225 (1.2)		92 (1.6)	41 (0.7)	133 (1.1)	
**Diagnosed Heart Disease**								
No	10578 (97.4)	7825 (97.9)	18403 (97.6)	**0.037**	5739 (99.0)	5784 (99.2)	11523 (99.1)	0.193
Yes	282 (2.6)	170 (2.1)	452 (2.4)		59 (1.0)	46 (0.8)	105 (0.9)	
**Diagnosed High Blood Pressure**								
No	9448 (87.0)	7339 (91.8)	16787 (89.0)	**<0.001**	5337 (92.1)	5606 (96.2)	10943 (94.1)	<0.001
Yes	1412 (13.0)	656 (8.2)	2068 (11.0)		461 (8.0)	224 (3.8)	685 (5.9)	
**Diagnosed Stroke**								
No	10784 (99.3)	7941 (99.3)	18725 (99.3)	0.841	5750 (99.2)	5782 (99.2)	11532 (99.2)	0.978
Yes	76 (0.7)	54 (0.7)	130 (0.7)		48 (0.8)	48 (0.8)	96 (0.8)	
**Piped water at home**								
No ~~Piped water~~	6,906 (63.6)	7,404 (92.6)	14,310 (75.9)	**<0.001**	3,404 (58.7)	5,345 (91.7)	8,749 (75.2)	<0.001
Yes ~~Piped water~~	3,954 (36.4)	591 (7.4)	4,545 (24.1)		2,394 (41.3)	485 (8.3)	2,879 (24.8)	
**Smoking Status**								
Never smoked	10,790 (99.4)	7,971 (99.7)	18,761 (99.5)	**0.001**	4,850 (83.7)	4,777 (81.9)	9,627 (82.8)	0.001
Former smoker (stopped more than 6 months ago)	43 (0.4)	10 (0.1)	53 (0.3)		442 (7.6)	262 (4.5)	704 (6.1)	
Current (in last 6 months)	27 (0.3)	14 (0.2)	41 (0.2)		506 (8.7)	791 (13.6)	1,297 (11.2)	

**Smoker in household**								
No	9,722 (89.5)	6,755 (84.5)	16,477 (87.4)	**<0.001**	5,305 (91.5)	5,249 (90.0)	10,554 (90.8)	**0.006**
Yes	1,138 (10.5)	1,240 (15.5)	2,378 (12.6)		493 (8.5)	581 (10.0)	1,074 (9.2)	
**Firewood smoke exposure**								
no/former/little exposure	1003 (9.4)	266 (3.4)	1269 (6.8)	**<0.001**	1240 (21.8)	2392 (43.6)	3632 (32.5)	**<0.001**
exposed, ventilated cooking area	8474 (79.3)	7541 (96.0)	16015 (86.3)		3874 (68.1)	3073 (56.0)	6947 (62.1)	
exposed non-ventilated cooking area	1216 (11.4)	49 (0.6)	1265 (6.8)		576 (10.1)	24 (0.4)	600 (5.4)	

- P-values were calculated by using Pearson χ2 test with 1 degree of freedom or Mann Whitney U test for continuous variables.
- Values in boldface indicate P<0.05
- **Missing data for:**
  - **Age group:** 1 urban female participant
  - **Body Mass Index:** 544 rural and 577 urban participants
  - **Firewood smoke exposure:** 490 rural and 275 urban participants

Formatted Table

Formatted: List Paragraph, Add space between paragraphs of the same style, Bulleted + Level: 1 + Aligned at: 0.25" + Indent at: 0.5"

Formatted: Font: (Default) +Body (Calibri), 11 pt

Formatted: Font: (Default) +Body (Calibri), 11 pt

Formatted: List Paragraph, Add space between paragraphs of the same style, Bulleted + Level: 1 + Aligned at: 0.25" + Indent at: 0.5", Don't suppress line numbers

Formatted: Font: (Default) Calibri, 8 pt

Formatted: List Paragraph, Bulleted + Level: 1 + Aligned at: 0.25" + Indent at: 0.5", Don't suppress line numbers

Formatted: Font: (Default) Calibri, 8 pt

Formatted: List Paragraph, Add space between paragraphs of the same style, Bulleted + Level: 2 + Aligned at: 0.75" + Indent at: 1", Don't suppress line numbers

Formatted: Font: (Default) Calibri, 8 pt

Formatted: List Paragraph, Add space between paragraphs of the same style, Bulleted + Level: 2 + Aligned at: 0.75" + Indent at: 1", Don't suppress line numbers

Compared to rural participants, urban males and females had significantly higher values when it came to markers of socio-economic position (median household income, median household possessions and proportion who completed tertiary education or were self-employed / employed).

With regards to NCD comorbidities, diagnosed high blood pressure was significantly greater in urban females (13.0%) compared to rural females (8.2%). The same pattern was observed for urban versus rural males. Overall, the proportion of participants reporting NCD comorbidities was relatively low (<3%).

~~The majority of~~Over 35% of urban participants of both sexes reported having piped water as a main source of household water compared to under 10% of rural participants~~ of both sexes~~. Current smoking in our study population was low overall but higher in males (11.2%) compared to females (0.2%). More rural females (15.5%) reported being exposed to household second-hand smoke compared to urban females (10.5%). When it came to firewood exposure, more urban females (11.4%) reported firewood smoke exposure in a non-ventilated cooking area compared to rural females (0.6%). The same pattern was observed for urban males (10.1%) compared to rural males (0.4%) for this factor.

Prevalence of asthma and related symptoms

Diagnosed asthma was reported in 5.1%~~, 95%CI (4.9% - 5.6%)~~ of urban and 4.5%~~, 95% CI (4.1% - 4.8%)~~ of rural participants. As shown in Table 2, a significantly higher proportion of urban females (5.6%) reported diagnosed asthma compared to rural females (4.5%) (p=0.001) but no difference was observed for males (p=0.871). Median age of asthma diagnosis was higher in rural females (17.0 years, IQR [6.5 - 28.0]) compared to urban females (14.0 years, IQR [5.0 - 23.0] and much lower in both male groups. Only 2.1% of participants reported regular use of asthma medication (for ~~example;~~example, aminophylline, salbutamol, ~~prednisone~~prednisone, or inhalers). As shown in Fig 1, 71.9% (1067/1484)

of those with diagnosed asthma did not report having symptoms of ‘wheeze in the past 12 months’ or ‘wheeze in the past 12 months plus shortness of breath’.

Table 2. Asthma outcomes and symptoms stratified by sex and urban-rural area

Asthma Outcomes	Females (N=18,855)				Males (N=11,628)			
	LILONGWE – URBAN	KARONGA - RURAL	TOTAL	p-value	LILONGWE – URBAN	KARONGA – RURAL	TOTAL	p-value
Diagnosed asthma (Yes)	611 (5.6)	362 (4.5)	973 (5.2)	**0.001**	253 (4.4)	258 (4.4)	511 (4.4)	0.871
Age of asthma diagnosis (years), median (IQR) subset	14.0 (5.0 – 23.0)	17.0 (6.5 – 28.0)	15.0 (5.0 – 25.0)	**<0.001**	11.0 (4.0 – 19.8)	12.0 (5.0 – 25.0)	12.0 (5.0 – 22.0)	0.073
Diagnosed asthma with medication (Yes)	256 (2.4)	186 (2.3)	442 (2.3)	0.890	82 (1.4)	101 (1.7)	183 (1.6)	0.168
Wheeze in the past 12 months (Yes)	371 (3.4)	257 (3.2)	628 (3.3)	0.446	168 (2.9)	193 (3.3)	361 (3.1)	0.199
Wheeze in the past 12 months plus shortness of breath (Yes)	501 (4.6)	262 (3.3)	763 (4.1)	**<0.001**	237 (4.1)	177 (3.0)	414 (3.6)	**0.002**
Asthma plus Wheeze in the past 12 months (Yes)	135 (1.2)	101 (1.3)	236 (1.3)	0.902	46 (0.8)	66 (1.1)	112 (1.0)	0.062
Asthma diagnosed < 18 years (Yes)	340 (59.9)	147 (50.2)	487 (56.6)	**0.007**	166 (71.6)	135 (61.6)	301 (66.7)	**0.026**

- P-values were calculated by using Pearson χ2 test with 1 degree of freedom or Mann Whitney U test for continuous variables.
- -Values in boldface indicate P<0.05

Formatted Table

Formatted: List Paragraph, Bulleted + Level: 1 + Aligned at: 0.25" + Indent at: 0.5"

Fig 1. Overlap between 'Diagnosed asthma'' and related symptoms

Fig 1. Figure is a proportional Venn diagram showing shows the overlap between the outcomes 'Diagnosed asthma', 'Wheeze in the past 12 months' and 'Wheeze in the past 12 months plus shortness of breath'. The area representing each variable is proportional to the number of people reporting this outcome.

Poor overlap was also seen with the outcome 'diagnosed asthma with medication use' where 59.8% (374/625) did not report having 'wheeze in the past 12 months' or 'wheeze in the past 12 months plus shortness of breath (S1 Fig).

Factors associated with a self-reported prior asthma diagnosis

S1 Table shows the results of univariable logistic regression analysis of potential risk factors associated with a self-reported diagnosed asthma stratified by sex and urban-rural area. The factors found to be associated with asthma (p<0.100) were age-group, BMI, physical activity rating, household income, household asset index, education, work status, diagnosed heart disease, diagnosed blood pressure, piped water at home, smoking statusstatus, and firewood smoke exposure.

Demographic, anthropometricanthropometric, and socioeconomic factors

As shown in Table 3, after adjusting for other factors, and considering a final significant level of 1%Bonferroni corrected significance level of (p=0.0041), in urban females only, being obese was associated with increased odds of diagnosed asthma compared to being normal weight (OR = 1.59, 95%CI 1.26 – 2.01, p<0.001). Moderate physical activity rating compared to low rating was negatively associated with diagnosed asthma in rural males only (OR = 0.31, 95%CI 0.14 – 0.72, p=0.007). In rural females, a secondary school education (OR = 2.10, 95%CI 1.35 – 3.27, p=0.001) and completing primary standard 6-8 (OR = 1.68, 95%CI 1.14 – 2.48, p=0.009) werewas positively associated with diagnosed asthma compared to completing primary Standard 1-5, respectively. By contrast, urban females completing primary Standard 6-8 had reduced odds of reported diagnosed asthma (OR = 0.58,

95%CI 0.41 – 0.81, p=0.001) compared to those completing primary Standard 1-5. No significant associated were observed for education and asthma when it came to the male groups.

Table 3. Multivariable analysis - Factors associated with diagnosed asthma stratified by sex & urban-rural area

Factors	Females				Males			
	LILONGWE - URBAN		KARONGA - RURAL		LILONGWE - URBAN		KARONGA - RURAL	
	Adjusted Odds ratio (95%CI)	p-value	Adjusted Odds ratio (95%CI)	p-value	Adjusted Odds ratio (95%CI)	p-value	Adjusted Odds ratio (95%CI)	p-value
**Age group**								
18-29	1.00	-	1.00	-	1.00	-	1.00	-
30-39	1.14 (0.92 - 1.41)	0.225	1.43 (1.06 - 1.92)	0.020	0.99 (0.69 - 1.43)	0.958	1.14 (0.81 - 1.61)	0.439
40-49	0.94 (0.69 - 1.28)	0.685	1.34 (0.94 - 1.91)	0.102	1.03 (0.66 - 1.62)	0.883	0.63 (0.39 - 1.00)	0.051
50-59	1.42 (0.98 - 2.04)	0.063	1.05 (0.67 - 1.65)	0.843	0.75 (0.40 - 1.39)	0.364	1.02 (0.62 - 1.67)	0.945
60-69	1.17 (0.69 - 1.99)	0.556	1.92 (1.17 - 3.15)	0.010	0.59 (0.27 - 1.31)	0.199	0.85 (0.43 - 1.68)	0.637
70+	1.51 (0.74 - 3.08)	0.259	0.84 (0.39 - 1.83)	0.663	0.56 (0.19 - 1.69)	0.307	1.13 (0.58 - 2.19)	0.727
**BMI (WHO categories)**								
Underweight (<18.5)	0.93 (0.57 - 1.52)	0.775	1.26 (0.83 - 1.92)	0.276	1.08 (0.66 - 1.76)	0.750	1.52 (1.02 - 2.27)	0.040
Normal(18.5-24.9)	1.00	-	1.00	-	1.00	-	1.00	-
Overweight(25.0-29.9)	1.30 (1.05 - 1.60)	0.014	0.91 (0.68 - 1.24)	0.561	0.91 (0.62 - 1.34)	0.647	0.93 (0.57 - 1.53)	0.786
Obese(>30)	**1.59 (1.26 - 2.01)**	**<0.001**	1.20 (0.81 - 1.80)	0.367	0.71 (0.35 - 1.47)	0.359	1.05 (0.32 - 3.49)	0.933
**Physical activity rating**								
Low	1.00	-	1.00	-	1.00	-	1.00	-
Moderate	0.71 (0.33 - 1.51)	0.367	1.01 (0.29 - 3.51)	0.987	1.10 (0.56 - 2.15)	0.783	0.31 (0.14 - 0.72)	0.007
High	0.73 (0.40 - 1.36)	0.321	1.56 (0.52 - 4.73)	0.430	0.74 (0.39 - 1.41)	0.368	0.56 (0.27 - 1.12)	0.102
**Household income**	1.00 (0.97 - 1.04)	0.800	1.00 (0.95 - 1.06)	0.966	1.03 (0.97 - 1.09)	0.322	1.08 (1.01 - 1.15)	0.017
**Household asset index**	1.04 (0.99 - 1.08)	0.094	0.99 (0.94 - 1.04)	0.667	1.05 (0.98 - 1.12)	0.149	1.02 (0.96 - 1.08)	0.528
**Education**								
No formal	0.52 (0.30 - 0.91)	0.022	1.19 (0.64 - 2.22)	0.584	4.07 (0.98 - 16.82)	0.053	0.50 (0.07 - 3.88)	0.510
Primary: Standard 1-5	1.00	-	1.00	-	1.00	-	1.00	-
Primary: Standard 6-8	**0.58 (0.41 - 0.81)**	**0.001**	1.68 (1.14 - 2.48)	0.009	2.73 (0.96 - 7.82)	0.061	1.48 (0.84 - 2.60)	0.170
Secondary	0.93 (0.69 - 1.26)	0.648	**2.10 (1.35 - 3.27)**	**0.001**	2.43 (0.87 - 6.75)	0.089	1.74 (0.98 - 3.07)	0.058
Tertiary	1.44 (1.00 - 2.08)	0.048	2.69 (0.99 - 7.32)	0.053	3.39 (1.19 - 9.67)	0.023	1.86 (0.80 - 4.32)	0.150
**Work Status**								
Not working	1.00	-	1.00	-	1.00	-	1.00	-
Housework	1.09 (0.85 - 1.40)	0.486	0.76 (0.45 - 1.28)	0.294	1.08 (0.62 - 1.89)	0.790	2.41 (1.03 - 5.66)	0.043
Farming/fishing	0.75 (0.18 - 3.20)	0.703	0.77 (0.51 - 1.17)	0.225	0.86 (0.11 - 6.57)	0.885	0.93 (0.64 - 1.36)	0.703
Self-employed	1.32 (1.00 - 1.76)	0.054	0.85 (0.53 - 1.38)	0.518	1.13 (0.75 - 1.70)	0.548	0.77 (0.47 - 1.25)	0.285
Employed	1.19 (0.90 - 1.58)	0.228	1.23 (0.60 - 2.52)	0.572	0.76 (0.52 - 1.10)	0.145	1.16 (0.69 - 1.95)	0.573
**Diagnosed Heart Disease**								
No	1.00	-	1.00	-	1.00	-	1.00	-
Yes	1.76 (1.16 - 2.68)	0.008	**2.30 (1.32 - 4.02)**	**0.003**	1.17 (0.36 - 3.84)	0.792	0.96 (0.23 - 4.04)	0.952
**Diagnosed High Blood Pressure**								
No	1.00	-	1.00	-	1.00	-	1.00	-
Yes	0.93 (0.72 - 1.21)	0.611	1.22 (0.80 - 1.85)	0.357	1.16 (0.70 - 1.92)	0.567	0.98 (0.49 - 1.95)	0.951
**Piped water at home**								
No ~~Piped water~~	1.00	-	1.00	-	1.00	-	1.00	-
Yes ~~Piped water~~	1.05 (0.85 - 1.29)	0.658	1.23 (0.84 - 1.82)	0.286	0.92 (0.67 - 1.26)	0.601	1.18 (0.78 - 1.81)	0.431
**Smoking Status**								
Never smoked	1.00		1.00		1.00		1.00	
Former smoker (stopped more than 6 months ago)	4.17 (1.94 - 8.94)	<0.001	*no output*	-	1.06 (0.66 - 1.70)	0.822	1.09 (0.59 - 2.00)	0.787
Current (in last 6 months)	0.81 (0.11 - 6.09)	0.839	*no output*	-	0.85 (0.52 - 1.41)	0.534	**0.46 (0.27 - 0.79)**	**0.004**
**Firewood smoke exposure**								
No/former/little exposure	1.00	-	1.00	-	1.00	-	1.00	-
Exposed, ventilated cooking area	1.41 (1.04 - 1.90)	0.027	0.65 (0.35 - 1.23)	0.189	0.81 (0.60 - 1.11)	0.189	1.00 (0.76 - 1.32)	0.993
Exposed, non-ventilated cooking area	1.07 (0.72 - 1.60)	0.733	0.72 (0.15 - 3.43)	0.684	0.64 (0.37 - 1.11)	0.113	0.96 (0.13 - 7.27)	0.966

- **Final model adjusted for**: age-group, BMI, physical activity rating, household income, household asset index, education, work status, diagnosed heart disease, diagnosed high blood pressure, piped water at home, smoking ~~status~~status, and firewood smoke exposure
- Adjusted ORs, 95% CIs and p-values in boldface indicate model results where $p<0.004$ (significance level threshold after Bonferroni correction)
- ~~Adjusted ORs, 95% CIs and p-values in boldface indicate model results where p<0.01~~
- *no output*: models with no output for this level due to small numbers

Formatted Table

Formatted: Font: Not Bold

Formatted: Font: Not Bold

Formatted: Font: Not Bold

Formatted: Underline

Other NCD comorbidities

We observed that previously diagnosed heart disease (yes versus no) was positively associated ~~with reported diagnosed asthma in urban females (OR=1.76, 95%CI 1.16 – 2.68, p=0.008) and~~ diagnosed asthma in rural females (OR=2.30, 95%CI 1.32 – 4.02, p=0.003). A similar observation was made in urban females, but this was not significant if we considered multiple testing. No association was seen in ~~but not in either urban males or rural males~~either male group with regards to any NCD comorbidity.

Environmental factors – water source, smoke exposure

In urban females, being a former smoker compared to having never smoked, was associated with diagnosed~~an~~ asthma ~~diagnosis~~ (OR=4.17, 95%CI [1.94 – 8.94], p<0.001). However, the n~~this covariate has to be considered with caution since the n~~umber of females who were smokers/former smokers in this population was relatively small. In rural males only, being a current smoker (in the last 6 months) compared to having never smoked had an inverse association with asthma diagnosis (OR = 0.46, 95% CI [0.27 – 0.79], p=0.004).

Factors associated with wheeze in the past 12 months

S2 Table shows the results of univariable logistic regression analysis of potential ~~risk~~ factors associated with reported wheeze in the past 12 months stratified by sex and urban-rural area. All factors found to be associated with wheeze (p<0.100) were explored further in multivariable analysis. The factors included in the final multivariable model were age-group, BMI, physical activity rating, household income, household asset index, education, work status, diagnosed heart disease, diagnosed blood pressure, diagnosed stroke, piped water at home, smoking ~~status~~status, and firewood smoke exposure.

Demographic, ~~anthropometric~~anthropometric, and socioeconomic factors

~~In contrast with 'reported diagnosed asthma', no~~No association was observed between being obese (compared to normal weight) and 'wheeze in the past 12 months' in urban females (Table 4). Increasing household income as a linear variable was significantly associated with wheeze in the past

Formatted: Font: (Default) +Body (Calibri), 11 pt, Not Bold, Font color: Auto

Formatted: Justified, Level 1, Line spacing: Double

12 months among rural males (OR=1.13, 95%CI 1.06 – 1.22, p<0.001). When it came to household possessions, increasing 'household asset index' was positively associated with 'wheeze in the past 12 months' in urban females only (OR=1.09, 95%CI 1.03 – 1.15, p=0.003). Unlike with self-diagnosed asthma, educational status was not associated with -wheeze in the past 12 months.

Table 4. Multivariable analysis -Factors associated with 'Wheeze in the past 12 months' stratified by sex & urban-rural area

Factors	Female				Male			
	LILONGWE ~~-~~ – URBAN		KARONGA - RURAL		LILONGWE - URBAN		KARONGA - RURAL	
	Adjusted Odds ratio (95%CI)	p-value	Adjusted Odds ratio (95%CI)	p-value	Adjusted Odds ratio (95%CI)	p-value	Adjusted Odds ratio (95%CI)	p-value
**Age group**								
18-29	1.00	-	1.00	-	1.00	-	1.00	-
30-39	0.98 (0.74 - 1.29)	0.874	1.32 (0.90 - 1.93)	0.157	1.14 (0.72 - 1.79)	0.575	0.98 (0.65 - 1.48)	0.933
40-49	1.00 (0.68 - 1.47)	0.992	1.56 (1.03 - 2.36)	0.037	1.17 (0.67 - 2.05)	0.581	1.22 (0.77 - 1.92)	0.398
50-59	1.35 (0.87 - 2.08)	0.180	1.41 (0.87 - 2.30)	0.165	1.56 (0.82 - 2.96)	0.175	0.83 (0.44 - 1.56)	0.567
60-69	0.61 (0.30 - 1.22)	0.160	1.71 (0.98 - 3.00)	0.060	1.56 (0.71 - 3.43)	0.264	1.55 (0.79 - 3.01)	0.200
70+	1.01 (0.43 - 2.36)	0.982	0.63 (0.28 - 1.42)	0.262	1.57 (0.55 - 4.53)	0.401	1.34 (0.64 - 2.83)	0.439
**BMI (WHO categories)**								
Underweight (<18.5)	1.06 (0.60 - 1.90)	0.832	1.18 (0.71 - 1.96)	0.528	1.25 (0.72 - 2.18)	0.422	1.25 (0.77 - 2.03)	0.358
Normal(18.5-24.9)	1.00	-	1.00	-	1.00	-	1.00	-
Overweight(25.0-29.9)	1.25 (0.96 - 1.62)	0.094	1.00 (0.71 - 1.40)	0.991	0.65 (0.39 - 1.09)	0.104	1.45 (0.89 - 2.34)	0.134
Obese(>30)	1.15 (0.85 - 1.57)	0.358	1.19 (0.76 - 1.85)	0.445	0.58 (0.22 - 1.51)	0.266	2.11 (0.73 - 6.15)	0.169
**Physical activity rating**								
Low	1.00	-	1.00	-	1.00	-	1.00	-
Moderate	1.77 (0.76 - 4.12)	0.189	0.80 (0.27 - 2.34)	0.680	2.61 (0.97 - 7.01)	0.058	0.42 (0.17 - 1.07)	0.068
High	0.81 (0.37 - 1.75)	0.589	0.82 (0.31 - 2.18)	0.695	1.76 (0.66 - 4.65)	0.257	0.64 (0.29 - 1.44)	0.284
**Household income**	1.02 (0.98 - 1.07)	0.303	1.03 (0.96 - 1.09)	0.434	1.08 (1.00 - 1.16)	0.045	**1.13 (1.06 - 1.22)**	**<0.001**
**Household asset index**	**1.09 (1.03 - 1.15)**	**0.003**	1.02 (0.97 - 1.08)	0.392	1.01 (0.94 - 1.09)	0.792	0.96 (0.89 - 1.02)	0.184
**Education**								
No formal	0.69 (0.37 - 1.30)	0.256	1.14 (0.62 - 2.06)	0.677	*no output*	-	1.11 (0.25 - 5.03)	0.892
Primary: Standard 1-5	1.00	-	1.00	-	1.00	-	1.00	-
Primary: Standard 6-8	0.64 (0.43 - 0.96)	0.030	0.98 (0.66 - 1.44)	0.905	1.26 (0.57 - 2.76)	0.570	1.25 (0.68 - 2.29)	0.472
Secondary	0.73 (0.50 - 1.06)	0.101	1.07 (0.66 - 1.72)	0.782	0.85 (0.40 - 1.82)	0.680	1.59 (0.86 - 2.93)	0.138
Tertiary	0.85 (0.54 - 1.35)	0.489	1.67 (0.57 - 4.93)	0.349	0.98 (0.43 - 2.23)	0.957	0.88 (0.30 - 2.58)	0.821
**Work Status**								
Not working								
Housework	0.79 (0.58 - 1.08)	0.143	0.47 (0.24 - 0.91)	0.025	0.16 (0.04 - 0.65)	0.011	0.85 (0.20 - 3.69)	0.829
Farming/fishing	3.12 (1.23 - 7.90)	0.016	0.66 (0.41 - 1.06)	0.086	0.96 (0.12 - 7.40)	0.965	0.96 (0.61 - 1.49)	0.841
Self-employed	0.98 (0.69 - 1.39)	0.917	0.81 (0.46 - 1.41)	0.455	0.91 (0.56 - 1.47)	0.690	0.85 (0.49 - 1.47)	0.559
Employed	0.84 (0.59 - 1.20)	0.338	1.26 (0.56 - 2.82)	0.579	0.65 (0.42 - 1.01)	0.055	1.10 (0.60 - 2.01)	0.762
**Diagnosed heart disease**								
No	1.00	-	1.00	-	1.00	-	1.00	-
Yes	**4.84 (3.34 - 7.00)**	**<0.001**	**2.37 (1.32 - 4.26)**	**0.004**	**4.76 (2.15 - 10.56)**	**<0.001**	**5.59 (2.49 - 12.57)**	**<0.001**
**Diagnosed High Blood Pressure**								
No	1.00	-	1.00	-	1.00	-	1.00	-
Yes	1.30 (0.96 - 1.76)	0.096	**2.24 (1.50 - 3.33)**	**<0.001**	0.71 (0.37 - 1.36)	0.300	1.45 (0.73 - 2.90)	0.287
**Diagnosed Stroke**								
No	1.00	-	1.00	-	1.00	-	1.00	-
Yes	0.82 (0.28 - 2.39)	0.710	1.36 (0.40 - 4.66)	0.621	1.51 (0.34 - 6.68)	0.590	1.06 (0.24 - 4.72)	0.938
**Piped water at home**								
No ~~Piped water~~	1.00	-	1.00	-	1.00	-	1.00	-
Yes ~~Piped water~~	0.99 (0.77 - 1.29)	0.955	0.76 (0.45 - 1.27)	0.291	0.92 (0.63 - 1.35)	0.661	0.97 (0.58 - 1.64)	0.915
**Smoking status**								
Never smoked	1.00	-	1.00	-				
Former smoker (stopped more than 6 months ago)	1.06 (0.25 - 4.59)	0.933	*no output*	-	1.16 (0.67 - 2.01)	0.605	1.27 (0.67 - 2.43)	0.463
Current (in last 6 months)	1.43 (0.19 - 10.72)	0.731	8.00 (1.57 - 40.90)	0.012	1.23 (0.73 - 2.08)	0.428	1.21 (0.78 - 1.86)	0.401
**Firewood smoke exposure**								
No/former/little exposure	1.00	-	1.00	-	1.00	-	1.00	-

Formatted Table

Exposed, ventilated cooking area	1.02 (0.73 - 1.44)	0.900	0.90 (0.44 - 1.84)	0.772	0.67 (0.46 - 0.97)	0.033	1.55 (1.12 - 2.14)	[illegible]
Exposed, non-ventilated cooking area	0.88 (0.55 - 1.39)	0.575	*no output*	-	1.06 (0.62 - 1.82)	0.820	No output	-

- **Final model adjusted for**: age-group, BMI, physical activity rating, household income, household asset index, education, work status, diagnosed heart disease, diagnosed high blood pressure, piped water at home, smoking ~~status~~status, and firewood smoke exposure.
- Adjusted ORs, 95% CIs and p-values in boldface indicate model results where ~~where p<0.01~~
- p<0.004 (significance level threshold after Bonferroni correction)
- *no output*: models with no output for this level due to small numbers

Formatted: Font: Not Bold

Formatted: Underline

Other NCD comorbidities

In all groups, reported diagnosed heart disease was significantly associated with wheeze in the past 12 months. This was observed for rural females (OR=2.37, 95%CI 1.32 – 4.26, p=0.004), urban females (OR=4.84, 95%CI 3.34 – 7.00, p<0.001), urban males (OR=5.59, 95%CI 2.49 – 12.57, p<0.001) and rural males (OR=4.76, 95%CI 2.15 – 10.56, p<0.001). We also observed that a prior diagnosis of high blood pressure was associated with reported wheeze in the past 12 months in rural females only (OR=2.24, 95%CI 1.50 – 3.33, p<0.001).

Environmental factors – water source, smoke exposure

There was no strong evidence for an association between smoking status and wheeze outcomes. Exposure to firewood in ventilated cooking area was associated with reported wheeze in the past 12 months in rural males only but this did not reach significance after correcting for multiple testing (OR=1.55, 95%CI 1.12 – 2.14, p=0.008).

Discussion

In this large community-based survey of Malawian adults, we observed only a slightly higher prevalence of diagnosed asthma in urban compared to rural dwelling adults~~, which~~ which is in contrasts to ~~with~~ findings from elsewhere in Sub-Saharan Africa~~on the African continent~~ where asthma has been strongly linked to urbanisation and ~~a so-called~~the adoption of a so-called western lifestyle [17-19, 21]. It is important to note that differences in underlying socioeconomic structures between African countries make direct comparisons challenging. For example, in Malawi, universally constrained access to healthcare and diagnostics may contribute to the lack of distinct urban-rural differences. In addition, the socioeconomic distribution of our study population may not be comparable to other studies that may have included urban dwellers of higher socioeconomic status with greater access to health-care [43, 44].

In our analysis we stratified our findings by both sex and urban-rural study area. The rationale for this was that published findings from our population cohort observed differences in the prevalence of disease outcomes such as hypertension according to urban-rural study area as well as sex [34]. Additionally, our preliminary analysis indicated that some sociodemographic indicators varied by both urban-rural area and sex.

We observed poor overlap between diagnosed asthma and asthma symptoms (reported wheeze in the past 12 months and wheeze in the past 12 months plus shortness of breath). This could indicate that most participants who had previously ~~ith a previous~~ diagno~~si~~sed with~~of~~ asthma were asymptomatic within the year prior to the questionnaire interview. It is also possible that reported wheeze may not be related to diagnosed asthma in our population. Additionally, other studies from Malawi have found a notable burden of abnormal lung function due to other chronic respiratory conditions such as chronic obstructive pulmonary disorder (COPD) [29, 30, 32].

The older age of asthma diagnosis in rural compared to urban dwellers that ~~we noted in our study~~we noted could reflect a true higher prevalence of adult-onset asthma in the rural area. Nonetheless, it

Formatted: Not Highlight

Formatted: Not Highlight

Formatted: Not Highlight

Formatted: Not Highlight

Formatted: Not Highlight

Formatted: Not Highlight

Formatted: Not Highlight

Formatted: Not Highlight

Formatted: Not Highlight

may also reflect some differences in health-seeking behaviours and improved access to healthcare in our rural area~~s~~ over time. We also observed an association between self-reported diagnosed heart disease and asthma which is consistent with other investigations [45-47]. Similar to our own investigation, other studies report that the association is stronger among adult females compared to males [47]. Although the underlying mechanisms linking asthma and heart disease, particularly in females, remain unclear, an inflammatory cytokine milieu and oestrogen-modulated inflammation may play roles [48]. We also found an association between wheeze in the past 12 months and diagnosed heart disease in all groups. Due to lack of access to diagnostics within health centres in Malawi, an asthma and heart disease diagnoses are based mainly on clinical observations by health-care professionals and symptoms that may be similar.~~,~~ H~~h~~ence some misclassification of these conditions is inevitable [49].

We did not observe a link between diagnosed high blood pressure and reported asthma in any group although a link between these two conditions has been established [50]. Interestingly, we did observe an association between diagnosed high blood pressure and reported wheeze in the past 12 months in rural females. However, this observation may not be linked to asthma given the lack of overlap between asthma and wheeze in our study.

Our observed strong association between obesity (BMI>30kg/m^2), and diagnosed asthma in urban females has been seen in other parts of the world [51, 52]. Although obesity is thought to affect the metabolic and immunological pathways that lead to asthma, specific mechanisms involved remain unclear [53]. One possible pathway is through the action of adipokines (cytokines produced by adipose tissue in obese individuals), which may affect the inflammatory process in the lungs and therefore asthma pathogenesis [53]. Nonetheless, the association between excess adiposity and asthma was not observed in urban males. This might reflect a female predisposition to adult-onset asthma, as observed in other parts of the world [54, 55] . However, we cannot rule out a ~~although there is also~~

potential ~~l for ~~misdiagnosis of asthma in obese individuals since both conditions are characterised by breathlessness [56].

In our study population, the relationship between socioeconomic status (SES) and diagnosed asthma was not consistent. This could ~~be a reflection of~~reflect the limitations of our SES assessment tools or an indication that a clear relationship did not exist. We did however observe that higher education appeared to be associated with asthma in urban females. It is possible that educational level was a better proxy for some of the socioeconomic factors associated with increased risk of asthma and this was apparent in urban females because of the high numbers of this group in our study. It could also reflect more health-seeking behaviour in urban females compared to their rural counterparts or better access to health-care facilities in an urban setting compared to a rural environment.

When we examined physical activity, we observed a negative association between moderate physical activity (compared to low activity) on diagnosed asthma in rural males but this did not reach significance after taking into account multiple testing. ~~, reflecting either the reduced capacity or willingness to exercise in asthmatics or that reduced activity is associated with an increased risk of asthma. The fact that this observation was made in rural males and not females, perhaps reflects that women are unable to avoid activity given their domestic expectations. Notably, longitudinal studies on physical activity and asthma incidence conducted in other parts of the world have had mixed findings. For example, a 10 year follow-up in females enrolled in the Nurses' Health Study in the United States found that interventions on BMI and physical activity had a modest impact on the risk of adult-onset asthma in that population [57] while an 11 year cohort study in Norway did not observe statistically significant associations of physical activity or change in physical activity with incident asthma in adults [58].~~

Regarding smoking in our study, few females, especially in the rural area, were current smokers / former smokers which reflects~~s~~ the cultural norms in Malawi and has also been observed in other studies ~~from Malawi ~~[29, 57]. However, we did observe that being a former smoker was associated

with diagnosed asthma in urban females which may result fromindicate smoking cessation advice or personal reflection at the time of diagnosis and therefore be an example ofs. reverse causation [58]. At the same time, this finding could also reflect social desirability effects since -individuals with asthma may underreport some behaviours such as smoking due to perceived or actual social disapproval [59]. This observation would have to be interpreted with caution given the small numbers. ThisHowever, our -observations regarding smoking in female participants would have to beshould be interpreted with caution given the small numbers. Our study findings also pointed to a 'protective effect' of current smoking on diagnosed asthma in rural males which again may n, might indicate that diagnosed asthmatics were less likely to smoke or were potentially underreporting their smoking habits.

take up or continue smoking. We did not observe any links between smoking and wheeze in our study. Other studies conducted in Malawi have looked at the effect of smoking on respiratory symptoms (cough, phlegm, wheeze or dyspnoea) and have observed a positive associations between being a current or ex-smoker and cough (without a cold) in urban adults in Blantyre, Malawi [29] as well as between 'smoking ever' and cough (but not wheeze) in rural adults in Chikwawa District [60].

Indoor solid biomass (crop waste, dung, firewood, charcoal etc) fuel smoke exposure has been associated with increased risk of respiratory conditions in LMICs especially [61, 62]. Although indoor biomass fuel exposure has been linked to COPD risk [63], the relationship with asthma is unclear [64]. In our investigation we found no significant association between exposure to firewood in a non-ventilated cooking area and diagnosed asthma. We , but we did see indications of a a positive relationship between this exposure and wheeze in the past 12 months among rural males. Given the poor overlap between asthma and wheeze in our investigation, this observation may not be related to asthma.

The strengths of our study include the use of a standard well-validated questionnaire and having a large sample size that enabled multivariable analysis as well as urban versus rural comparisons. Our study had some limitations including our reliance on self-reported diagnosis of asthma, wheeze and

disease onset which may have resulted in some misclassification and potential information bias. Additionally, clinical assessment of asthma using spirometry was not available and we were not able to differentiate between asthma, COPD or even heart failure [49]. Therefore, there will be some attenuation of results due to misclassification. Moreover, there are likely to be some unmeasured confounders which may explain our findings. Lastly, the cross-sectional nature of the study means that we cannot mitigate the effects of reverse-causality. Hence, observed associations should be interpreted with caution.

Conclusions

Despite limitations, our findings highlight relationships between excess body weight as well as comorbidities such as reported heart disease and diagnosed asthma in Malawi among females. Future investigations using longitudinally collected data and clinical measures for asthma diagnosis are needed to better understand these observations and to inform healthcare delivery services for asthma in -urban and rural Malawi.

Acknowledgements

We are very grateful to study participants and community leaders for taking part in our investigation. We are also thankful to Malawi Epidemiology and Intervention Research Unit (previously known as Karonga Prevention Study) research clinicians, interviewers, research assistants and all other staff who were part of gathering data for the study.

References

1. Beasley R, Semprini A, Mitchell EA. Risk factors for asthma: is prevention possible? Lancet (London, England). 2015;386(9998):1075-85. Epub 2015/09/19. doi: 10.1016/s0140-6736(15)00156-7. PubMed PMID: 26382999.

2. WHO. Asthma Geneva: World Health Organisation; 2023 [updated 4 May 2023; cited 2023 21 June 2023]. Available from: https://www.who.int/news-room/fact-sheets/detail/asthma.

3. von Mutius E. Gene-environment interactions in asthma. The Journal of allergy and clinical immunology. 2009;123(1):3-11; quiz 2-3. Epub 2009/01/10. doi: 10.1016/j.jaci.2008.10.046. PubMed PMID: 19130922.

4. Johansson H, Mersha TB, Brandt EB, Khurana Hershey GK. Interactions between environmental pollutants and genetic susceptibility in asthma risk. Current opinion in immunology. 2019;60:156-62. Epub 2019/08/31. doi: 10.1016/j.coi.2019.07.010. PubMed PMID: 31470287.

5. Guilleminault L, Williams EJ, Scott HA, Berthon BS, Jensen M, Wood LG. Diet and Asthma: Is It Time to Adapt Our Message? Nutrients. 2017;9(11). Epub 2017/11/09. doi: 10.3390/nu9111227. PubMed PMID: 29117118; PubMed Central PMCID: PMCPMC5707699.

6. Peters U, Dixon AE, Forno E. Obesity and asthma. The Journal of allergy and clinical immunology. 2018;141(4):1169-79. Epub 2018/04/09. doi: 10.1016/j.jaci.2018.02.004. PubMed PMID: 29627041; PubMed Central PMCID: PMCPMC5973542.

7. Collaborators GRF. Global, regional, and national comparative risk assessment of 84 behavioural, environmental and occupational, and metabolic risks or clusters of risks, 1990-2016: a systematic analysis for the Global Burden of Disease Study 2016. Lancet (London, England). 2017;390(10100):1345-422. Epub 2017/09/19. doi: 10.1016/s0140-6736(17)32366-8. PubMed PMID: 28919119; PubMed Central PMCID: PMCPMC5614451.

8. Sheehan WJ, Phipatanakul W. Indoor allergen exposure and asthma outcomes. Current opinion in pediatrics. 2016;28(6):772-7. Epub 2016/11/03. doi: 10.1097/mop.0000000000000421. PubMed PMID: 27653703; PubMed Central PMCID: PMCPMC5127595.

9. Asher MI, Keil U, Anderson HR, Beasley R, Crane J, Martinez F, et al. International Study of Asthma and Allergies in Childhood (ISAAC): rationale and methods. The European respiratory journal. 1995;8(3):483-91. Epub 1995/03/01. PubMed PMID: 7789502.

10. Weiland SK, Bjorksten B, Brunekreef B, Cookson WO, von Mutius E, Strachan DP. Phase II of the International Study of Asthma and Allergies in Childhood (ISAAC II): rationale and methods. The European respiratory journal. 2004;24(3):406-12. Epub 2004/09/11. doi: 10.1183/09031936.04.00090303. PubMed PMID: 15358699.

11. Ellwood P, Asher MI, Beasley R, Clayton TO, Stewart AW. The international study of asthma and allergies in childhood (ISAAC): phase three rationale and methods. The international journal of tuberculosis and lung disease : the official journal of the International Union against Tuberculosis and Lung Disease. 2005;9(1):10-6. Epub 2005/01/29. PubMed PMID: 15675544.

12. de Nijs SB, Venekamp LN, Bel EH. Adult-onset asthma: is it really different? European Respiratory Review. 2013;22(127):44-52. doi: 10.1183/09059180.00007112.

13. Chowdhury NU, Guntur VP, Newcomb DC, Wechsler ME. Sex and gender in asthma. Eur Respir Rev. 2021;30(162). Epub 20211117. doi: 10.1183/16000617.0067-2021. PubMed PMID: 34789462; PubMed Central PMCID: PMCPMC8783601.

14. Rasmussen F, Hancox RJ. Mechanisms of obesity in asthma. Current opinion in allergy and clinical immunology. 2014;14(1):35-43. Epub 2013/12/05. doi: 10.1097/aci.0000000000000024. PubMed PMID: 24300417.

15. Wang L, Wang K, Gao X, Paul TK, Cai J, Wang Y. Sex difference in the association between obesity and asthma in U.S. adults: Findings from a national study. Respiratory Medicine. 2015;109(8):955-62. doi: https://doi.org/10.1016/j.rmed.2015.06.001.

16. Brumpton B, Langhammer A, Romundstad P, Chen Y, Mai X-M. General and abdominal obesity and incident asthma in adults: the HUNT study. European Respiratory Journal. 2013;41(2):323-9. doi: 10.1183/09031936.00012112.

17. Morgan BW, Siddharthan T, Grigsby MR, Pollard SL, Kalyesubula R, Wise RA, et al. Asthma and Allergic Disorders in Uganda: A Population-Based Study Across Urban and Rural Settings. J Allergy Clin Immunol Pract. 2018;6(5):1580-7.e2. Epub 20180201. doi: 10.1016/j.jaip.2017.11.032. PubMed PMID: 29361510; PubMed Central PMCID: PMCPMC6050146.

18. Siddharthan T, Grigsby M, Morgan B, Kalyesubula R, Wise RA, Kirenga B, et al. Prevalence of chronic respiratory disease in urban and rural Uganda. Bull World Health Organ. 2019;97(5):318-27. Epub 20190326. doi: 10.2471/blt.18.216523. PubMed PMID: 31551628; PubMed Central PMCID: PMCPMC6747035.

19. Kirenga BJ, de Jong C, Katagira W, Kasozi S, Mugenyi L, Boezen M, et al. Prevalence and factors associated with asthma among adolescents and adults in Uganda: a general population based survey. BMC Public Health. 2019;19(1):227. doi: 10.1186/s12889-019-6562-2.

20. Miranda JJ, Bernabe-Ortiz A, Gilman RH, Smeeth L, Malaga G, Wise RA, et al. Multimorbidity at sea level and high-altitude urban and rural settings: The CRONICAS Cohort Study. J Comorb. 2019;9:2235042x19875297. Epub 20191106. doi: 10.1177/2235042x19875297. PubMed PMID: 34249770; PubMed Central PMCID: PMCPMC8240099.

21. Desalu OO, Adeoti AO, Ojuawo OB, Aladesanmi AO, Oguntoye MS, Afolayan OJ, et al. Urban-Rural Differences in the Epidemiology of Asthma and Allergies in Nigeria: A Population-Based Study. J Asthma Allergy. 2021;14:1389-97. Epub 20211110. doi: 10.2147/jaa.S333133. PubMed PMID: 34866916; PubMed Central PMCID: PMCPMC8637762.

22. Buist AS, Vollmer WM, Sullivan SD, Weiss KB, Lee TA, Menezes AM, et al. The Burden of Obstructive Lung Disease Initiative (BOLD): rationale and design. Copd. 2005;2(2):277-83. Epub 2006/12/02. PubMed PMID: 17136954.

23. Buist AS, McBurnie MA, Vollmer WM, Gillespie S, Burney P, Mannino DM, et al. International variation in the prevalence of COPD (the BOLD Study): a population-based prevalence study. Lancet (London, England). 2007;370(9589):741-50. Epub 2007/09/04. doi: 10.1016/s0140-6736(07)61377-4. PubMed PMID: 17765523.

24. Mortimer K, Reddel HK, Pitrez PM, Bateman ED. Asthma management in low- and middle-income countries: case for change. European Respiratory Journal. 2022:2103179. doi: 10.1183/13993003.03179-2021.

25. Coleman M. Teaching corner: Adult asthma in Malawi. Malawi medical journal : the journal of Medical Association of Malawi. 2011;23(1):22-4. Epub 2011/03/01. PubMed PMID: 23638253; PubMed Central PMCID: PMCPmc3615331.

26. Cook J, Jefferis O, Matchere P, Mbale E, Rylance J. Sickle-cell disease in Malawian children is associated with restrictive spirometry: a cross-sectional survey. The international journal of tuberculosis and lung disease : the official journal of the International Union against Tuberculosis and Lung Disease. 2013;17(9):1235-8. Epub 2013/07/06. doi: 10.5588/ijtld.12.0965. PubMed PMID: 23827770; PubMed Central PMCID: PMCPmc3826120.

27. Wroe EB, Kalanga N, Mailosi B, Mwalwanda S, Kachimanga C, Nyangulu K, et al. Leveraging HIV platforms to work toward comprehensive primary care in rural Malawi: the Integrated Chronic Care Clinic. Healthcare (Amsterdam, Netherlands). 2015;3(4):270-6. Epub 2015/12/25. doi: 10.1016/j.hjdsi.2015.08.002. PubMed PMID: 26699356.

28. Manjomo RC, Mwagomba B, Ade S, Ali E, Ben-Smith A, Khomani P, et al. Managing and monitoring chronic non-communicable diseases in a primary health care clinic, Lilongwe, Malawi. Public health action. 2016;6(2):60-5. Epub 2016/07/01. doi: 10.5588/pha.16.0003. PubMed PMID: 27358797; PubMed Central PMCID: PMCPmc4913686.

29. Meghji J, Nadeau G, Davis KJ, Wang D, Nyirenda MJ, Gordon SB, et al. Noncommunicable Lung Disease in Sub-Saharan Africa. A Community-based Cross-Sectional Study of Adults in Urban Malawi. American journal of respiratory and critical care medicine. 2016;194(1):67-76. Epub 2016/01/21. doi: 10.1164/rccm.201509-1807OC. PubMed PMID: 26788760; PubMed Central PMCID: PMCPMC4960629.

30. Nightingale R, Lesosky M, Flitz G, Rylance SJ, Meghji J, Burney P, et al. Noncommunicable Respiratory Disease and Air Pollution Exposure in Malawi (CAPS). A Cross-Sectional Study. American journal of respiratory and critical care medicine. 2019;199(5):613-21. Epub 2018/08/25. doi: 10.1164/rccm.201805-0936OC. PubMed PMID: 30141966; PubMed Central PMCID: PMCPMC6396863.

31. Rylance S, Jewell C, Naunje A, Mbalume F, Chetwood JD, Nightingale R, et al. Non-communicable respiratory disease and air pollution exposure in Malawi: a prospective cohort study. Thorax. 2020;75(3):220-6. Epub 2020/02/23. doi: 10.1136/thoraxjnl-2019-213941. PubMed PMID: 32079666; PubMed Central PMCID: PMCPMC7063402.

32. Njoroge MW, Rylance S, Nightingale R, Gordon S, Mortimer K, Burney P, et al. Cohort profile: The Chikwawa lung health cohort; a population-based observational non-communicable respiratory disease study of adults in Malawi. PLoS One. 2020;15(11):e0242226. Epub 2020/11/13. doi: 10.1371/journal.pone.0242226. PubMed PMID: 33180873; PubMed Central PMCID: PMCPMC7660567.

33. Crampin AC, Kayuni N, Amberbir A, Musicha C, Koole O, Tafatatha T, et al. Hypertension and diabetes in Africa: design and implementation of a large population-based study of burden and risk factors in rural and urban Malawi. Emerging themes in epidemiology. 2016;13:3. Epub 2016/02/04. doi: 10.1186/s12982-015-0039-2. PubMed PMID: 26839575; PubMed Central PMCID: PMCPmc4736489.

34. Price AJ, Crampin AC, Amberbir A, Kayuni-Chihana N, Musicha C, Tafatatha T, et al. Prevalence of obesity, hypertension, and diabetes, and cascade of care in sub-Saharan Africa: a cross-sectional, population-based study in rural and urban Malawi. The lancet Diabetes & endocrinology. 2018;6(3):208-22. Epub 2018/01/27. doi: 10.1016/s2213-8587(17)30432-1. PubMed PMID: 29371076; PubMed Central PMCID: PMCPMC5835666.

35. Crampin AC, Dube A, Mboma S, Price A, Chihana M, Jahn A, et al. Profile: the Karonga Health and Demographic Surveillance System. International journal of epidemiology. 2012;41(3):676-85. Epub 2012/06/26. doi: 10.1093/ije/dys088. PubMed PMID: 22729235; PubMed Central PMCID: PMCPmc3396313.

36. National Statistical Office. 2018 Malawi Population and Housing: Main Report. Lilongwe: National Statistical Office, 2019.

37. Burney PG, Laitinen LA, Perdrizet S, Huckauf H, Tattersfield AE, Chinn S, et al. Validity and repeatability of the IUATLD (1984) Bronchial Symptoms Questionnaire: an international comparison. The European respiratory journal. 1989;2(10):940-5. Epub 1989/11/01. PubMed PMID: 2606194.

38. Amberbir A, Medhin G, Hanlon C, Britton J, Venn A, Davey G. Frequent use of paracetamol and risk of allergic disease among women in an Ethiopian population. PLoS One. 2011;6(7):e22551. Epub 2011/08/04. doi: 10.1371/journal.pone.0022551. PubMed PMID: 21811632; PubMed Central PMCID: PMCPMC3141069.

39. WHO. Physical activity Geneva, Switzerland World Health Organisation 2022 [updated 5 October 2022; cited 2022 12 November 2022]. Available from: https://www.who.int/news-room/fact-sheets/detail/physical-activity.

40. WHO. Global Database on Body Mass Index: BMI classification Geneva: World Health Organisation; 2006 [cited 2016]. Available from: http://apps.who.int/bmi/index.jsp?introPage=intro_3.html.

41. McLean E, Dube A, Saul J, Branson K, Luhanga M, Mwiba O, et al. Implementing electronic data capture at a well-established health and demographic surveillance site in rural northern Malawi. Glob Health Action. 2017;10(1):1367162. doi: 10.1080/16549716.2017.1367162. PubMed PMID: 28922071; PubMed Central PMCID: PMCPMC5645702.

42. Katz MH. Multivariable Analysis: A Practical Guide for Clinicians Cambridge: Cambridge University Press; 2007.

43. Addo-Yobo EO, Woodcock A, Allotey A, Baffoe-Bonnie B, Strachan D, Custovic A. Exercise-induced bronchospasm and atopy in Ghana: two surveys ten years apart. PLoS medicine. 2007;4(2):e70. Epub 2007/03/01. doi: 10.1371/journal.pmed.0040070. PubMed PMID: 17326711; PubMed Central PMCID: PMCPMC1808098.

44. Poyser MA, Nelson H, Ehrlich RI, Bateman ED, Parnell S, Puterman A, et al. Socioeconomic deprivation and asthma prevalence and severity in young adolescents. European Respiratory Journal. 2002;19(5):892-8. doi: 10.1183/09031936.02.00238402.

45. Iribarren C, Tolstykh IV, Eisner MD. Are patients with asthma at increased risk of coronary heart disease? International journal of epidemiology. 2004;33(4):743-8. doi: 10.1093/ije/dyh081.

46. Iribarren C, Tolstykh IV, Miller MK, Sobel E, Eisner MD. Adult Asthma and Risk of Coronary Heart Disease, Cerebrovascular Disease, and Heart Failure: A Prospective Study of 2 Matched Cohorts. American Journal of Epidemiology. 2012;176(11):1014-24. doi: 10.1093/aje/kws181.

47. Wang L, Gao S, Yu M, Sheng Z, Tan W. Association of asthma with coronary heart disease: A meta analysis of 11 trials. PLoS One. 2017;12(6):e0179335. Epub 20170613. doi: 10.1371/journal.pone.0179335. PubMed PMID: 28609456; PubMed Central PMCID: PMCPMC5469478.

48. Gurgone D, McShane L, McSharry C, Guzik TJ, Maffia P. Cytokines at the Interplay Between Asthma and Atherosclerosis? Front Pharmacol. 2020;11:166. Epub 20200304. doi: 10.3389/fphar.2020.00166. PubMed PMID: 32194407; PubMed Central PMCID: PMCPMC7064545.

49. Wong CW, Tafuro J, Azam Z, Satchithananda D, Duckett S, Barker D, et al. Misdiagnosis of Heart Failure: A Systematic Review of the Literature. Journal of Cardiac Failure. 2021;27(9):925-33. doi: https://doi.org/10.1016/j.cardfail.2021.05.014.

50. Zolotareva O, Saik OV, Königs C, Bragina EY, Goncharova IA, Freidin MB, et al. Comorbidity of asthma and hypertension may be mediated by shared genetic dysregulation and drug side effects. Sci Rep. 2019;9(1):16302. Epub 20191108. doi: 10.1038/s41598-019-52762-w. PubMed PMID: 31705029; PubMed Central PMCID: PMCPMC6841742.

51. Koebnick C, Fischer H, Daley MF, Ferrara A, Horberg MA, Waitzfelder B, et al. Interacting effects of obesity, race, ethnicity and sex on the incidence and control of adult-onset asthma. Allergy, asthma, and clinical immunology : official journal of the Canadian Society of Allergy and Clinical Immunology. 2016;12:50. Epub 2016/10/26. doi: 10.1186/s13223-016-0155-8. PubMed PMID: 27777591; PubMed Central PMCID: PMCPMC5069790.

52. Sood A, Qualls C, Schuyler M, Arynchyn A, Alvarado JH, Smith LJ, et al. Adult-onset asthma becomes the dominant phenotype among women by age 40 years. the longitudinal CARDIA study. Annals of the American Thoracic Society. 2013;10(3):188-97. Epub 2013/06/28. doi: 10.1513/AnnalsATS.201212-115OC. PubMed PMID: 23802814; PubMed Central PMCID: PMCPMC3960903.

53. Umetsu DT. Mechanisms by which obesity impacts asthma. Thorax. 2016. Epub 2016/09/28. doi: 10.1136/thoraxjnl-2016-209130. PubMed PMID: 27672120.

54. Leynaert B, Sunyer J, Garcia-Esteban R, Svanes C, Jarvis D, Cerveri I, et al. Gender differences in prevalence, diagnosis and incidence of allergic and non-allergic asthma: a population-based cohort. Thorax. 2012;67(7):625-31. doi: 10.1136/thoraxjnl-2011-201249.

55. Hansen S, Probst-Hensch N, Keidel D, Dratva J, Bettschart R, Pons M, et al. Gender differences in adult-onset asthma: results from the Swiss SAPALDIA cohort study. European Respiratory Journal. 2015;46(4):1011-20. doi: 10.1183/13993003.02278-2014.

56. Currow DC, Dal Grande E, Sidhu C, Ekström M, Johnson MJ. The independent association of overweight and obesity with breathlessness in adults: a cross-sectional, population-based study. The European respiratory journal. 2017;50(3). Epub 20170927. doi: 10.1183/13993003.00558-2017. PubMed PMID: 28954771.

57. Banda HT, Thomson R, Mortimer K, Bello GAF, Mbera GB, Malmborg R, et al. Community prevalence of chronic respiratory symptoms in rural Malawi: Implications for policy. PLoS One. 2017;12(12):e0188437. Epub 2017/12/08. doi: 10.1371/journal.pone.0188437. PubMed PMID: 29216193; PubMed Central PMCID: PMCPMC5720679.

58. Verlato G, Nguyen G, Marchetti P, Accordini S, Marcon A, Marconcini R, et al. Smoking and New-Onset Asthma in a Prospective Study on Italian Adults. Int Arch Allergy Immunol. 2016;170(3):149-57. Epub 20160818. doi: 10.1159/000446509. PubMed PMID: 27532893.

59. Messeri P, Cantrell J, Mowery P, Bennett M, Hair E, Vallone D. Examining differences in cigarette smoking prevalence among young adults across national surveillance surveys. PLOS ONE. 2019;14(12):e0225312. doi: 10.1371/journal.pone.0225312.

60. Nightingale R, Lesosky M, Flitz G, Rylance SJ, Meghji J, Burney P, et al. Non-Communicable Respiratory Disease and Air Pollution Exposure in Malawi (CAPS): A Cross-Sectional Study. American journal of respiratory and critical care medicine. 2018. Epub 2018/08/25. doi: 10.1164/rccm.201805-0936OC. PubMed PMID: 30141966.

61. Gordon SB, Bruce NG, Grigg J, Hibberd PL, Kurmi OP, Lam KB, et al. Respiratory risks from household air pollution in low and middle income countries. Lancet Respir Med. 2014;2(10):823-60. Epub 20140902. doi: 10.1016/s2213-2600(14)70168-7. PubMed PMID: 25193349; PubMed Central PMCID: PMCPMC5068561.

62. Po JYT, FitzGerald JM, Carlsten C. Respiratory disease associated with solid biomass fuel exposure in rural women and children: systematic review and meta-analysis. Thorax. 2011;66(3):232-9. doi: 10.1136/thx.2010.147884.

63. Holtjer JCS, Bloemsma LD, Beijers R, Cornelissen MEB, Hilvering B, Houweling L, et al. Identifying risk factors for COPD and adult-onset asthma: an umbrella review. Eur Respir Rev. 2023;32(168). Epub 20230503. doi: 10.1183/16000617.0009-2023. PubMed PMID: 37137510; PubMed Central PMCID: PMCPMC10155046.

64. Rylance S, Masekela R, Banda NPK, Mortimer K. Determinants of lung health across the life course in sub-Saharan Africa. The international journal of tuberculosis and lung disease : the official journal of the International Union against Tuberculosis and Lung Disease. 2020;24(9):892-901. doi: 10.5588/ijtld.20.0083. PubMed PMID: 33156755.

Supporting Information

- **S1 Fig. Overlap between 'Diagnosed asthma with medication use' and related symptoms**
  Figure is a proportional Venn diagram showings the overlap between the outcomes 'Diagnosed asthma with medication use', 'Wheeze in the past 12 months' and 'Wheeze in the past 12 months plus shortness of breath'. - The area representing each variable is proportional to the number of people reporting this outcome.

Formatted: Font: Italic

Formatted: Font: Italic

- **S1 Table - Univariable Analysis - Factors associated with diagnosed asthma stratified by sex & urban-rural area**

- **S2 Table - Univariable Analysis - Factors associated with wheeze in the past 12 months stratified by sex & urban-rural area**

29 January 2024

Dear Dr. Amugsi,

Please find below a rebuttal letter addressing comments raised by reviewers in relation to our manuscript entitled *Factors associated with self-reported diagnosed asthma in urban and rural Malawi: observations from a population-based study of non-communicable diseases.*

Sincerely,

Abena S. Amoah

On behalf of co-authors

Rebuttal Letter

PGPH-D-23-01533
Factors associated with self-reported diagnosed asthma in urban and rural Malawi: observations from a population-based study of non-communicable diseases
PLOS Global Public Health

Comments to the Author
1. Does this manuscript meet PLOS Global Public Health's publication criteria? Is the manuscript technically sound, and do the data support the conclusions? The manuscript must describe methodologically and ethically rigorous research with conclusions that are appropriately drawn based on the data presented.

Reviewer #1: Partly

Reviewer #2: Yes

Reviewer #3: Yes

Response: Prior to our first submission of the manuscript, we reviewed PLOS Global Public Health publication criteria and verified that our draft manuscript met the outlined publication criteria.

2. Has the statistical analysis been performed appropriately and rigorously?

Reviewer #1: No

Reviewer #2: Yes

Reviewer #3: Yes

Response: We seek guidance and further elucidation from Reviewer #1 on how to improve our statistical analysis approach to ensure it is performed appropriately and rigorously.

3. Have the authors made all data underlying the findings in their manuscript fully available (please refer to the Data Availability Statement at the start of the manuscript PDF file)?

The PLOS [Data policy](#) requires authors to make all data underlying the findings described in their manuscript fully available without restriction, with rare exception. The data should be provided as part of the manuscript or its supporting information, or deposited to a public repository. For example, in addition to summary statistics, the data points behind *means, medians and variance measures should be available. If there are restrictions on publicly sharing data—e.g. participant privacy or use of data from a third party—those must be specified.*

Reviewer #1: No

Reviewer #2: Yes

Reviewer #3: Yes

Response: In the Data Availability Statement completed as part of our original submission, we provided a link to the data repository where the dataset analyzed can be download used in our analysis can be downloaded. By providing this source, we have made the data underlying the findings fully available.

4. Is the manuscript presented in an intelligible fashion and written in standard English?

PLOS Global Public Health does not copyedit accepted manuscripts, so the language in submitted articles must be clear, correct, and unambiguous. Any typographical or grammatical errors should be corrected at revision, so please note any specific errors here.

Reviewer #1: No

Reviewer #2: Yes

Reviewer #3: Yes

Response: We have corrected some minor grammatical errors but are somewhat perplexed that our submission was deemed not to be presented in an intelligible fashion nor written in standard English.

5. Review Comments to the Author

Please use the space provided to explain your answers to the questions above. You may also include additional comments for the author, including concerns about dual publication, research ethics, or publication ethics. (Please upload your review as an attachment if it exceeds 20,000 characters)

Reviewer #1: Review Reports
Title: Factors associated with self-reported diagnosed asthma in urban and rural Malawi: observations from a population-based study of non-communicable diseases.
Manuscript Number: PGPH-D-23-01533.

Review Comments

Response: We thank the reviewer for reading our manuscript and providing feedback as well as suggestions as to how to improve our manuscript.

Ø Is that published? E.g., the socio-demographic character is described in detail, however, it is extracted from the main survey.

Response: We thank the reviewer for the question. Descriptions of the study area, population and study design have been published in the following protocol paper:

- Crampin AC, Kayuni N, Amberbir A, Musicha C, Koole O, Tafatatha T, et al. Hypertension and diabetes in Africa: design and implementation of a large population-based study of burden and risk factors in rural and urban Malawi. Emerging themes in epidemiology. 2016;13:3. Epub 2016/02/04. doi: 10.1186/s12982-015-0039-2. PubMed PMID: 26839575; PubMed Central PMCID: PMCPmc4736489.

More extensive details about the sociodemograpic characteristics of our study population can be found in the following publication that focused on obesity, hypertension and diabetes:

- Price AJ, Crampin AC, Amberbir A, Kayuni-Chihana N, Musicha C, Tafatatha T, et al. Prevalence of obesity, hypertension, and diabetes, and cascade of care in sub-Saharan Africa: a cross-sectional, population-based study in rural and urban Malawi. The lancet Diabetes & endocrinology. 2018;6(3):208-22. Epub 2018/01/27. doi: 10.1016/s2213-8587(17)30432-1. PubMed PMID: 29371076; PubMed Central PMCID: PMCPMC5835666.

Both these publications were referenced in our initial submission as well as our revised manuscript.

Ø The three triads of symptoms of Bronchial asthma were not asked in the survey/ but you have included in the figure not in the methods section/. How you supported and synthesized (a) the respondent's response (b) the diagnosis in the survey (Who, How and Where and with what apparatus?

Response: We thank the reviewer for the question. In the methods section we did indicate that we did indeed ask about symptoms of asthma in our questionnaire. Symptoms asked in our questionnaire were "Wheeze in the past 12 months" as well as "Wheeze + shortness of breath" .The specific questions asked were:

1. “Have you had wheezing or whistling in your chest at any time in the past year?”
➔ Responses to this question were used for our variable “Wheeze in the past 12 months”
2. “In the last year, have you ever had an attack of wheezing or whistling that has made you feel short of breath?”
➔ Responses to this question were used for our variable “Wheeze in the past 12 months + Shortness of breath”

These two variables were used to generate the Venn diagrams that showed the overlap between our main variable “Diagnosed asthma” and “Diagnosed asthma with medication use”.

As stated in our discussion, one of the limitations of our survey is that we relied solely on self-reported diagnosis of asthma and we did have a clinical assessment of asthma through lung function testing by spirometry.

Line 367 – 371
Our study had some limitations including our reliance on self-reported diagnosis of asthma, wheeze and disease onset which may have resulted in some misclassification and potential information bias. Additionally, clinical assessment of asthma using spirometry was not available and we were not able to differentiate between asthma, COPD or even heart failure [49]

Ø *If it is assessment of risks, is that Descriptive or analytic? If analytic, what is the appropriate statistical approach and why you have used yours?*
Response: Given the cross-sectional nature of our study, our assessment of factors associated with asthma were only descriptive.

Ø *Lacks consistency across the document e.g., how you wrote the confidence interval, e.g., Asthma or Bronchial Asthma.*

Response: We thank the reviewer for pointing this out. We have adjusted our manuscript to remove the confidence intervals provided for asthma to ensure consistency as suggested.

Ø *Differentiate among those associations.*

Response: We thank the reviewer for this suggestion. We would however need clarification from the reviewer which associations they are asking for us to differentiate among. Further clarification would assist us in addressing the reviewer’s concern.

Ø *Avoid redundancies.*

Response: We thank the reviewer for this suggestion. We would however need examples of redundancies in our text. Further clarification will assist us in addressing this concern.

Ø *Data quality assurance and measures taken were not addressed.*

Response: We thank the reviewer for raising this point. We have added some information on the quality assurance measures taken to the text. We have also included a reference for an article that extensively outlines the Malawi Epidemiology and Intervention Research Unit data management protocols.

- McLean E, Dube A, Saul J, Branson K, Luhanga M, Mwiba O, Kalobekamo F, Geis S, Crampin AC. Implementing electronic data capture at a well-established health and demographic surveillance site in rural northern Malawi. Glob Health Action. 2017;10(1):1367162. doi: 10.1080/16549716.2017.1367162.

Our text has been amended as follows:

Line 144 -152
Data management and quality assurance
Data was predominantly captured electronically via protected tablet computers using forms pre-programmed in Open Data Kit (https://getodk.org/) which were uploaded each day to a database housed in secure central server at each research site. Data quality was assured through rigorous training of Interviewers and the use of an interviewer's guide to ensure uniformity of data collection. Pre-programmed data checks were performed in the MEIRU data offices to identify any discrepancies which were addressed immediately. Uploaded data were also regularly extracted and checked for scientific validity. Data management processes were strictly controlled through adherence to set protocols and a central data dictionary [41].

Ø *What was done for those diagnosed with 'Asthma'?*

Response: As part of the NCD survey, all study participants found to have a chronic condition or an abnormal laboratory result were referred to the government health institution. At the same time, the Malawi Epidemiology and Intervention Research Unit (MEIRU) established noncommunicable disease (NCD) care clinics in the main primary health care centres in both the urban and rural sites.

Specific details of the referral system are outlined in:

- Crampin AC, Kayuni N, Amberbir A, Musicha C, Koole O, Tafatatha T, et al. Hypertension and diabetes in Africa: design and implementation of a large population-based study of burden and risk factors in rural and urban Malawi. Emerging themes in epidemiology. 2016;13:3. Epub 2016/02/04. doi: 10.1186/s12982-015-0039-2. PubMed PMID: 26839575; PubMed Central PMCID: PMCPmc4736489.

All participants with asthma were invited to attend NCD clinics supported by the Malawi Epidemiology and Intervention Research Unit (MEIRU) clinical staff. To this day, study participants are still provided with free clinical care and medication in both the urban and rural study sites. These NCD clinic days are run daily at Area 25 Health Centre in the capital city of Lilongwe for urban study participants and weekly for rural participants at Chilumba Rural Health in Chilumba, Karonga District.

Ø Tense, grammar, space and hyphens and units need improvement

Response: We have reviewed the manuscript to improve the grammar.

Ø Lacks key words.

Response: We have added keywords to the body of the manuscript text after the abstract.

Ø The tables are not self-explanatory

Response: We have modified our tables to improve clarity.

Ø The result needs further clarity and attractive ness to your reader. Try to assebmble the fragmented sub sections

Response: We humbly request specific guidance from the reviewer on how our results can be improved to provide "further clarity and attractiveness" to the reader. We purposely divided the results into subsections to provide some structure and organization in the delivery of the results.

Ø The discussion is incomplete for its contents and strength. In addition, it occupied wide large space.

Response: We humbly request specifics from the reviewer as to how our discussion is "incomplete for its contents and strength" and also "occupies large space". Further clarification and specifics from the reviewer are needed to address the concerns raised.

Reviewer #2: An applaudable article which meets the criteria for PLOS Global public health journal publication. The sample size justifies the attempt to answer the question posed with reported limitations. The concluded prevalence of self reported asthma in rural and urban Malawi challenges the myth/hypothesis that urbanization contributes to the rising asthma prevalence in low to middle income countries and this magnifies the relevance of the article. Although caution should be wielded before concluding such arguments, the conclusions drawn in this article are supported by data presented in the results section and analyzed with a robust statistical strategy. The cross-sectional design for the primary research question posed projects an ethically and methodologically sound design with mentioned limitations/weaknesses.

An marvellous attempt at statistical analysis of the data is seen through the sub-categorization of data captured from the questionnaires and successfully shows associations and correlations between the data. The summarized version of the presentation appears in very busy and congested tables but this is understandable particularly when considering the volume of data that was processed present the statistical analysis. Additionally, the manuscript is presented in good palatable english.

Response: We thank the reviewer for carefully reviewing our manuscript and providing critical and invaluable feedback. We also thank the reviewer for noting our effort to apply a

robust statistical strategy in our analyses. We do note that the tables are indeed busy, and we have tried to improve the presentation of our tables.

Reviewer #3: The paper emerges from the analysis of the database produced in the setting of a large study with appropriately described methodology and previously published results on other subjects. The study of noncommunicable diseases in Malawi, as in LMIC in general, and the focus on differences between rural and urban populations is very pertinent and important form the public health point of view.
Table 2 is very clear-cut and provides a simple and strong message.
The paragraph of Conclusions is also good.

Response: We thank the reviewer for meticulously reviewing our manuscript and providing critical and invaluable feedback. We also appreciate the suggestions made to improve our manuscript.

However, I have some suggestions that might help the authors improve the message of their analysis and results, which I share for consideration:

Is the inverse association between smoking and self-reported asthma the result of reverse causality? Or information bias due to social desirability effects? Or a mixture of both? What about moderate versus low physical activity in rural men (lines 222).

Response: The reviewer has raised an important point. Aside from reverse causality being an explanation for the inverse association between smoking and self-reported asthma, there is also the possibility of information bias due to social desirability effects. In other words, some study participants with asthma may have underreported their smoking habits due to social disapproval. Therefore, our observations could be a mixture of reverse causality and social desirability effects. We have added these points to our discussion:

Line 84 – the words "for which the objectives, design, populations and methodology are described elsewhere" in my opinion belong to the methods and not the background section.

Response: the reviewer is indeed correct. That line and that section have been moved to the start of the Methods section.

In the objectives (lines 89-90) I don't see the relevance of defining adult age as >=18. This should be clear in the methods but it does not pertain to the meaning of the objective, once the authors refer to "adults".

Response: We thank the reviewer for the suggestion. We have removed that wording from the introduction.

Lines 112-112 – was the question nr 1 exactly as stated in the article? The grammar sounds strange (for the verb diagnosed).

Response: This was an oversight. We have corrected the language in the text.

Line 111 -113
The specific questions related to asthma and its symptoms were:

1. *Have you ever been diagnosed with asthma, asthmatic bronchitis or allergic bronchitis by a doctor or other health professional? If yes, what age were you when first diagnosed?*

The rationale to look at interactions between several variables and sex/ urban-rural on asthma outcomes is not sufficiently justified. Not in the discussion but rather in the background of statistical analysis sections.

Response: We thank the reviewer for pointing this out. We have added some description of the interactions into the Discussion.

Line 294 – Line 298
In our analysis we stratified our findings by both sex and urban-rural study area. The rationale for this was that published findings from our population cohort observed differences in the prevalence of disease outcomes such as hypertension according to urban-rural study area as well as sex [34]. Additionally, our preliminary analysis indicated that some sociodemographic indicators varied by both urban-rural area and sex.

What is the rule for the choice of 1% as significance level when attempting to account for multiple testing? The authors did much more than 5 tests, so this significance level is still high when compared to the standard 5%.

Response: We have revised our analysis to apply a more stringent rule. We have used a Bonferroni Correction to take into account multiple testing as outlined by Katz:

Katz MH. Multivariable Analysis: A Practical Guide for Clinicians Cambridge: Cambridge University Press; 2007.

Our text has been adjusted accordingly:

Line 175 - 178
To account for the effect of multiple testing, we applied a Bonferroni correction where alpha of 5% was divided by the number variables in the final logistic regression model [42]. Therefore, a corrected alpha of 0.4% (p=0.004) was considered as the threshold for statistically significance for our multivariable regression analyses.

Lines 184-185 – the concept "majority of" means more than half. It is an absolute value, not a comparative one. So the idea of "compared to rural" in the final part of the sentence does not make sense.

Response: We thank the reviewer for raising this point. We have revised the text:

Line 202 - 203
Over 35% of urban participants of both sexes reported having piped water as a main source of household water compared to under 8% of rural participants of both sexes.

Figure 1 would be much better and more useful with a true Venn diagram, that is, with the areas of each state and overlaps proportional to the number of people in them.

Response: We thank the reviewer for the suggestion. The figures have been adjusted and redrawn as proportional Venn diagrams. We have also added the fact that these are proportional Venn diagrams to the text.

MINOR POINTS:
In the background, in the abstract and main text, the authors refer to available data in adults AS COMPARED TO CHILDREN. I argue that this point should not be seen from a relative but rather an absolute point of view. If it is insufficiently characterized in adults, it is worth the study. If it is less studied than in children but still there were enough data to deeply understand the subject, attention should be redirected to interventions.
Response: We thank the reviewer for point this out. We have tried to adjust the text to reflect the suggestion.

Line 132 – the word "all" in the beginning of line 132 seems unnecessary and strange. A mistake? This was indeed an error. This has been corrected. We thank the reviewer for pointing this out.

6. PLOS authors have the option to publish the peer review history of their article ([what does this mean?]). If published, this will include your full peer review and any attached files.

Do you want your identity to be public for this peer review? *If you choose "no", your identity will remain anonymous but your review may still be made public.*

For information about this choice, including consent withdrawal, please see our [Privacy Policy].

Reviewer #1: No

Reviewer #2: ***Yes:*** Benson Gombe

Reviewer #3: ***Yes:*** *Ana Azevedo*
