# Supplementary material for: Factors associated with self-reported diagnosed asthma in urban and rural Malawi: Observations from a population-based study of non-communicable diseases
Source: PLOS Glob Public Health. 2024 Jul 11;4(7):e0002952. doi: 10.1371/journal.pgph.0002952 (PMC11239063; doi:10.1371/journal.pgph.0002952)
Supplement: Attachment — Submitted filename: PGPH-D-23-01533 Rebuttal Letter.docx [file pgph.0002952.s004.docx]

29 January 2024

Dear Dr. Amugsi,

Please find below a rebuttal letter addressing comments raised by reviewers in relation to our manuscript entitled *Factors associated with self-reported diagnosed asthma in urban and rural Malawi: observations from a population-based study of non-communicable diseases.*

Sincerely,

Abena S. Amoah

*On behalf of co-authors*

_________________________________________________________________________

Rebuttal Letter

PGPH-D-23-01533
Factors associated with self-reported diagnosed asthma in urban and rural Malawi: observations from a population-based study of non-communicable diseases
PLOS Global Public Health

***Comments to the Author*** *1. Does this manuscript meet PLOS Global Public Health’s* [*publication criteria*](https://eur03.safelinks.protection.outlook.com/?url=https%3A%2F%2Fjournals.plos.org%2Fglobalpublichealth%2Fs%2Fcriteria-for-publication&data=05%7C02%7Ca.s.amoah%40lumc.nl%7Cb4ec428e3a2544227c4c08dc06bc5d64%7Cc4048c4fdd544cbd80495457aacd2fb8%7C0%7C0%7C638392653294735487%7CUnknown%7CTWFpbGZsb3d8eyJWIjoiMC4wLjAwMDAiLCJQIjoiV2luMzIiLCJBTiI6Ik1haWwiLCJXVCI6Mn0%3D%7C3000%7C%7C%7C&sdata=e0qZ2aSJD1eGq%2F8Xm8vCchTJqZj0JvbICAGFxBgrcvE%3D&reserved=0)*? Is the manuscript technically sound, and do the data support the conclusions? The manuscript must describe methodologically and ethically rigorous research with conclusions that are appropriately drawn based on the data presented.*

*Reviewer #1: Partly*

*Reviewer #2: Yes*

*Reviewer #3: Yes*

Response: Prior to our first submission of the manuscript, we reviewed PLOS Global Public Health publication criteria and verified that our draft manuscript met the outlined publication criteria.

*2. Has the statistical analysis been performed appropriately and rigorously?*

*Reviewer #1: No*

*Reviewer #2: Yes*

*Reviewer #3: Yes*

Response: We seek guidance and further elucidation from Reviewer #1 on how to improve our statistical analysis approach to ensure it is performed appropriately and rigorously.

*3. Have the authors made all data underlying the findings in their manuscript fully available (please refer to the Data Availability Statement at the start of the manuscript PDF file)?

The* [*PLOS Data policy*](https://eur03.safelinks.protection.outlook.com/?url=https%3A%2F%2Fjournals.plos.org%2Fglobalpublichealth%2Fs%2Fdata-availability&data=05%7C02%7Ca.s.amoah%40lumc.nl%7Cb4ec428e3a2544227c4c08dc06bc5d64%7Cc4048c4fdd544cbd80495457aacd2fb8%7C0%7C0%7C638392653294735487%7CUnknown%7CTWFpbGZsb3d8eyJWIjoiMC4wLjAwMDAiLCJQIjoiV2luMzIiLCJBTiI6Ik1haWwiLCJXVCI6Mn0%3D%7C3000%7C%7C%7C&sdata=dFuw%2Fa9NAoyO2OfhRajhBF67%2BafKVXVlNOrATzYGOEk%3D&reserved=0) *requires authors to make all data underlying the findings described in their manuscript fully available without restriction, with rare exception. The data should be provided as part of the manuscript or its supporting information, or* deposited to a public repository. For example, in addition to summary statistics, the data points behind *means, medians and variance measures should be available. If there are restrictions on publicly sharing data—e.g. participant privacy or use of data from a third party—those must be specified.*

*Reviewer #1: No*

*Reviewer #2: Yes*

*Reviewer #3: Yes*

Response: In the Data Availability Statement completed as part of our original submission, we provided a link to the data repository where the dataset analyzed can be download used in our analysis can be downloaded. By providing this source, we have made the data underlying the findings fully available.

*4. Is the manuscript presented in an intelligible fashion and written in standard English?

PLOS Global Public Health does not copyedit accepted manuscripts, so the language in submitted articles must be clear, correct, and unambiguous. Any typographical or grammatical errors should be corrected at revision, so please note any specific errors here.*

*Reviewer #1: No*

*Reviewer #2: Yes*

*Reviewer #3: Yes*

Response: We have corrected some minor grammatical errors but are somewhat perplexed that our submission was deemed not to be presented in an intelligible fashion nor written in standard English.

*5. Review Comments to the Author

Please use the space provided to explain your answers to the questions above. You may also include additional comments for the author, including concerns about dual publication, research ethics, or publication ethics. (Please upload your review as an attachment if it exceeds 20,000 characters)*

*Reviewer #1: Review Reports
Title: Factors associated with self-reported diagnosed asthma in urban and rural Malawi: observations from a population-based study of non-communicable diseases.
Manuscript Number: PGPH-D-23-01533.*

*Review Comments*

Response: We thank the reviewer for reading our manuscript and providing feedback as well as suggestions as to how to improve our manuscript.

*Ø Is that published? E.g., the socio-demographic character is described in detail, however, it is extracted from the main survey.*

Response: We thank the reviewer for the question. Descriptions of the study area, population and study design have been published in the following protocol paper:

- Crampin AC, Kayuni N, Amberbir A, Musicha C, Koole O, Tafatatha T, et al. Hypertension and diabetes in Africa: design and implementation of a large population-based study of burden and risk factors in rural and urban Malawi. Emerging themes in epidemiology. 2016;13:3. Epub 2016/02/04. doi: 10.1186/s12982-015-0039-2. PubMed PMID: 26839575; PubMed Central PMCID: PMCPmc4736489.

More extensive details about the sociodemograpic characteristics of our study population can be found in the following publication that focused on obesity, hypertension and diabetes:

- Price AJ, Crampin AC, Amberbir A, Kayuni-Chihana N, Musicha C, Tafatatha T, et al. Prevalence of obesity, hypertension, and diabetes, and cascade of care in sub-Saharan Africa: a cross-sectional, population-based study in rural and urban Malawi. The lancet Diabetes & endocrinology. 2018;6(3):208-22. Epub 2018/01/27. doi: 10.1016/s2213-8587(17)30432-1. PubMed PMID: 29371076; PubMed Central

PMCID: PMCPMC5835666*.*

Both these publications were referenced in our initial submission as well as our revised manuscript.

*Ø The three triads of symptoms of Bronchial asthma were not asked in the survey/ but you have included in the figure not in the methods section/. How you supported and synthesized (a) the respondent’s response (b) the diagnosis in the survey (Who, How and Where and with what apparatus?*

Response: We thank the reviewer for the question. In the methods section we did indicate that we did indeed ask about symptoms of asthma in our questionnaire. Symptoms asked in our questionnaire were “Wheeze in the past 12 months” as well as “Wheeze + shortness of breath” .The specific questions asked were:

1. “Have you had wheezing or whistling in your chest at any time in the past year?”

- Responses to this question were used for our variable “Wheeze in the past 12 months”

2. “In the last year, have you ever had an attack of wheezing or whistling that has made you feel short of breath?”

- Responses to this question were used for our variable “Wheeze in the past 12 months + Shortness of breath”

These two variables were used to generate the Venn diagrams that showed the overlap between our main variable “Diagnosed asthma” and “Diagnosed asthma with medication use”.

As stated in our discussion, one of the limitations of our survey is that we relied solely on self-reported diagnosis of asthma and we did have a clinical assessment of asthma through lung function testing by spirometry.

Line 367 – 371

Our study had some limitations including our reliance on self-reported diagnosis of asthma, wheeze and disease onset which may have resulted in some misclassification and potential information bias. Additionally, clinical assessment of asthma using spirometry was not available and we were not able to differentiate between asthma, COPD or even heart failure [49]

*Ø If it is assessment of risks, is that Descriptive or analytic? If analytic, what is the appropriate statistical approach and why you have used yours?*
Response: Given the cross-sectional nature of our study, our assessment of factors associated with asthma were only descriptive.

*Ø Lacks consistency across the document e.g., how you wrote the confidence interval, e.g., Asthma or Bronchial Asthma.*

Response: We thank the reviewer for pointing this out. We have adjusted our manuscript to remove the confidence intervals provided for asthma to ensure consistency as suggested.

*Ø Differentiate among those associations.*

Response: We thank the reviewer for this suggestion. We would however need clarification from the reviewer which associations they are asking for us to differentiate among. Further clarification would assist us in addressing the reviewer’s concern.

*Ø Avoid redundancies.*

Response: We thank the reviewer for this suggestion. We would however need examples of redundancies in our text. Further clarification will assist us in addressing this concern.

*Ø Data quality assurance and measures taken were not addressed.*

Response: We thank the reviewer for raising this point. We have added some information on the quality assurance measures taken to the text. We have also included a reference for an article that extensively outlines the Malawi Epidemiology and Intervention Research Unit data management protocols.

- McLean E, Dube A, Saul J, Branson K, Luhanga M, Mwiba O, Kalobekamo F, Geis S, Crampin AC. Implementing electronic data capture at a well-established health and demographic surveillance site in rural northern Malawi. Glob Health Action. 2017;10(1):1367162. doi: 10.1080/16549716.2017.1367162.

Our text has been amended as follows:

Line 144 -152

Data management and quality assurance

Data was predominantly captured electronically via protected tablet computers using forms pre-programmed in Open Data Kit (<https://getodk.org/>) which were uploaded each day to a database housed in secure central server at each research site. Data quality was assured through rigorous training of Interviewers and the use of an interviewer’s guide to ensure uniformity of data collection. Pre-programmed data checks were performed in the MEIRU data offices to identify any discrepancies which were addressed immediately. Uploaded data were also regularly extracted and checked for scientific validity. Data management processes were strictly controlled through adherence to set protocols and a central data dictionary [41].

*Ø What was done for those diagnosed with ‘Asthma’?*

Response: As part of the NCD survey, all study participants found to have a chronic condition or an abnormal laboratory result were referred to the government health institution. At the same time, the Malawi Epidemiology and Intervention Research Unit (MEIRU) established noncommunicable disease (NCD) care clinics in the main primary health care centres in both the urban and rural sites.

Specific details of the referral system are outlined in:

- Crampin AC, Kayuni N, Amberbir A, Musicha C, Koole O, Tafatatha T, et al. Hypertension and diabetes in Africa: design and implementation of a large population-based study of burden and risk factors in rural and urban Malawi. Emerging themes in epidemiology. 2016;13:3. Epub 2016/02/04. doi: 10.1186/s12982-015-0039-2. PubMed PMID: 26839575; PubMed Central PMCID: PMCPmc4736489.

All participants with asthma were invited to attend NCD clinics supported by the Malawi Epidemiology and Intervention Research Unit (MEIRU) clinical staff. To this day, study participants are still provided with free clinical care and medication in both the urban and rural study sites. These NCD clinic days are run daily at Area 25 Health Centre in the capital city of Lilongwe for urban study participants and weekly for rural participants at Chilumba Rural Health in Chilumba, Karonga District.

*Ø Tense, grammar, space and hyphens and units need improvement*

Response: We have reviewed the manuscript to improve the grammar.

*Ø Lacks key words.*

Response: We have added keywords to the body of the manuscript text after the abstract.

*Ø The tables are not self-explanatory*

Response: We have modified our tables to improve clarity.

*Ø The result needs further clarity and attractive ness to your reader. Try to assebmble the fragmented sub sections*

Response: We humbly request specific guidance from the reviewer on how our results can be improved to provide “further clarity and attractiveness” to the reader. We purposely divided the results into subsections to provide some structure and organization in the delivery of the results.

*Ø The discussion is incomplete for its contents and strength. In addition, it occupied wide large space.*

Response: We humbly request specifics from the reviewer as to how our discussion is “incomplete for its contents and strength” and also “occupies large space”. Further clarification and specifics from the reviewer are needed to address the concerns raised.

*Reviewer #2: An applaudable article which meets the criteria for PLOS Global public health journal publication. The sample size justifies the attempt to answer the question posed with reported limitations. The concluded prevalence of self reported asthma in rural and urban Malawi challenges the myth/hypothesis that urbanization contributes to the rising asthma prevalence in low to middle income countries and this magnifies the relevance of the article. Although caution should be wielded before concluding such arguments, the conclusions drawn in this article are supported by data presented in the results section and analyzed with a robust statistical strategy. The cross-sectional design for the primary research question posed projects an ethically and methodologically sound design with mentioned limitations/weaknesses.

An marvellous attempt at statistical analysis of the data is seen through the sub-categorization of data captured from the questionnaires and successfully shows associations and correlations between the data. The summarized version of the presentation appears in very busy and congested tables but this is understandable particularly when considering the volume of data that was processed present the statistical analysis. Additionally, the manuscript is presented in good palatable english.*

Response: We thank the reviewer for carefully reviewing our manuscript and providing critical and invaluable feedback. We also thank the reviewer for noting our effort to apply a robust statistical strategy in our analyses. We do note that the tables are indeed busy, and we have tried to improve the presentation of our tables.

*Reviewer #3: The paper emerges from the analysis of the database produced in the setting of a large study with appropriately described methodology and previously published results on other subjects. The study of noncommunicable diseases in Malawi, as in LMIC in general, and the focus on differences between rural and urban populations is very pertinent and important form the public health point of view.
Table 2 is very clear-cut and provides a simple and strong message.
The paragraph of Conclusions is also good.*

Response: We thank the reviewer for meticulously reviewing our manuscript and providing critical and invaluable feedback. We also appreciate the suggestions made to improve our manuscript.

*However, I have some suggestions that might help the authors improve the message of their analysis and results, which I share for consideration:

Is the inverse association between smoking and self-reported asthma the result of reverse causality? Or information bias due to social desirability effects? Or a mixture of both? What about moderate versus low physical activity in rural men (lines 222).*

Response: The reviewer has raised an important point. Aside from reverse causality being an explanation for the inverse association between smoking and self-reported asthma, there is also the possibility of information bias due to social desirability effects. In other words, some study participants with asthma may have underreported their smoking habits due to social disapproval. Therefore, our observations could be a mixture of reverse causality and social desirability effects. We have added these points to our discussion:

*Line 84 – the words “for which the objectives, design, populations and methodology are described elsewhere” in my opinion belong to the methods and not the background section.*

Response: the reviewer is indeed correct. That line and that section have been moved to the start of the Methods section.

*In the objectives (lines 89-90) I don’t see the relevance of defining adult age as >=18. This should be clear in the methods but it does not pertain to the meaning of the objective, once the authors refer to “adults”.*

Response: We thank the reviewer for the suggestion. We have removed that wording from the introduction.

*Lines 112-112 – was the question nr 1 exactly as stated in the article? The grammar sounds strange (for the verb diagnosed).*

Response: This was an oversight. We have corrected the language in the text.

Line 111 -113

The specific questions related to asthma and its symptoms were:

1. *Have you ever been diagnosed with asthma, asthmatic bronchitis or allergic bronchitis by a* *doctor or other health professional? If yes, what age were you when first diagnosed?*

*The rationale to look at interactions between several variables and sex/ urban-rural on asthma outcomes is not sufficiently justified. Not in the discussion but rather in the background of statistical analysis sections.*

Response: We thank the reviewer for pointing this out. We have added some description of the interactions into the Discussion.

Line 294 – Line 298

In our analysis we stratified our findings by both sex and urban-rural study area. The rationale for this was that published findings from our population cohort observed differences in the prevalence of disease outcomes such as hypertension according to urban-rural study area as well as sex [34]. Additionally, our preliminary analysis indicated that some sociodemographic indicators varied by both urban-rural area and sex.

*What is the rule for the choice of 1% as significance level when attempting to account for multiple testing? The authors did much more than 5 tests, so this significance level is still high when compared to the standard 5%.*

Response: We have revised our analysis to apply a more stringent rule. We have used a Bonferroni Correction to take into account multiple testing as outlined by Katz:

Katz MH. Multivariable Analysis: A Practical Guide for Clinicians Cambridge: Cambridge University Press; 2007.

Our text has been adjusted accordingly:

Line 175 - 178

To account for the effect of multiple testing, we applied a Bonferroni correction where alpha of 5% was divided by the number variables in the final logistic regression model [42]. Therefore, a corrected alpha of 0.4% (p=0.004) was considered as the threshold for statistically significance for our multivariable regression analyses.

*Lines 184-185 – the concept “majority of” means more than half. It is an absolute value, not a comparative one. So the idea of “compared to rural” in the final part of the sentence does not make sense.*

Response: We thank the reviewer for raising this point. We have revised the text:

Line 202 - 203

Over 35% of urban participants of both sexes reported having piped water as a main source of household water compared to under 8% of rural participants of both sexes.

*Figure 1 would be much better and more useful with a true Venn diagram, that is, with the areas of each state and overlaps proportional to the number of people in them.*
Response: We thank the reviewer for the suggestion. The figures have been adjusted and redrawn as proportional Venn diagrams. We have also added the fact that these are proportional Venn diagrams to the text.  *MINOR POINTS:
In the background, in the abstract and main text, the authors refer to available data in adults AS COMPARED TO CHILDREN. I argue that this point should not be seen from a relative but rather an absolute point of view. If it is insufficiently characterized in adults, it is worth the study. If it is less studied than in children but still there were enough data to deeply understand the subject, attention should be redirected to interventions.*
Response: We thank the reviewer for point this out. We have tried to adjust the text to reflect the suggestion.

*Line 132 – the word “all” in the beginning of line 132 seems unnecessary and strange. A mistake?* This was indeed an error. This has been corrected. We thank the reviewer for pointing this out.

*6. PLOS authors have the option to publish the peer review history of their article (*[*what does this mean?*](https://eur03.safelinks.protection.outlook.com/?url=https%3A%2F%2Fjournals.plos.org%2Fglobalpublichealth%2Fs%2Feditorial-and-peer-review-process%23loc-peer-review-history&data=05%7C02%7Ca.s.amoah%40lumc.nl%7Cb4ec428e3a2544227c4c08dc06bc5d64%7Cc4048c4fdd544cbd80495457aacd2fb8%7C0%7C0%7C638392653294735487%7CUnknown%7CTWFpbGZsb3d8eyJWIjoiMC4wLjAwMDAiLCJQIjoiV2luMzIiLCJBTiI6Ik1haWwiLCJXVCI6Mn0%3D%7C3000%7C%7C%7C&sdata=R4CYb1SgUz5%2BQjihtegp25xJ8Vg367L752ZPd4rvtzY%3D&reserved=0)*). If published, this will include your full peer review and any attached files.*


***Do you want your identity to be public for this peer review?*** *If you choose “no”, your identity will remain anonymous but your review may still be made public.


For information about this choice, including consent withdrawal, please see our* [*Privacy Policy*](https://eur03.safelinks.protection.outlook.com/?url=https%3A%2F%2Fwww.plos.org%2Fprivacy-policy&data=05%7C02%7Ca.s.amoah%40lumc.nl%7Cb4ec428e3a2544227c4c08dc06bc5d64%7Cc4048c4fdd544cbd80495457aacd2fb8%7C0%7C0%7C638392653294735487%7CUnknown%7CTWFpbGZsb3d8eyJWIjoiMC4wLjAwMDAiLCJQIjoiV2luMzIiLCJBTiI6Ik1haWwiLCJXVCI6Mn0%3D%7C3000%7C%7C%7C&sdata=KK8GmASSBL%2FXCQOCGmLmrSW96MsnHf7BASKrLo7Qmvg%3D&reserved=0)*.*

*Reviewer #1: No*

*Reviewer #2:* ***Yes:*** *Benson Gombe*

*Reviewer #3:* ***Yes:*** *Ana Azevedo*
